# Causes of Death After Colorectal Cancer Diagnosis: A Population-Based Study

**DOI:** 10.3389/fonc.2021.647179

**Published:** 2021-03-30

**Authors:** Yuqian Feng, Huimin Jin, Kaibo Guo, Harpreet S. Wasan, Shanming Ruan, Cihui Chen

**Affiliations:** ^1^The First Clinical College of Zhejiang Chinese Medical University, Hangzhou, China; ^2^Department of Cancer Medicine, Hammersmith Hospital, Imperial College Healthcare National Health Service Trust, London, United Kingdom; ^3^Department of Medical Oncology, The First Affiliated Hospital of Zhejiang Chinese Medical University, Hangzhou, China

**Keywords:** colorectal cancer, non-cancer deaths, surveillance, epidemiology, and End Results database, standardized mortality ratios, cardiovascular disease

## Abstract

**Background:** Non-cancer causes of death in patients with colorectal cancer (CRC) have not received much attention until now. The purpose of the current study is to investigate the non-cancer causes of death in patients with CRC at different periods of latency.

**Methods:** Eligible patients with CRC were included from the Surveillance, Epidemiology, and End Results (SEER) database, and standardized mortality ratios (SMRs) were calculated using the SEER*Stat software 8.3.8.

**Results:** A total of 475,771 patients with CRC were included, of whom 230,841 patients died during the follow-up period. Within 5 years, CRC was the leading cause of death. Over time, non-cancer causes of death account for an increasing proportion. When followed up for more than 10 years, non-cancer deaths accounted for 71.9% of all deaths worldwide. Cardiovascular diseases were the most common causes of non-cancer deaths, accounting for 15.4% of the total mortality. Patients had a significantly higher risk of death from septicemia within the first year after diagnosis compared with the general population (SMR, 3.39; 95% CI, 3.11–3.69). Within 5–10 years after CRC diagnosis, patients had a significantly higher risk of death from diabetes mellitus (SMR, 1.27; 95% CI, 1.19–1.36). During the course of more than 10 years, patients with CRC had a significantly higher risk of death from atherosclerosis (SMR 1.47; 95% CI, 1.11–1.9).

**Conclusions:** Although CRC has always been the leading cause of death in patients with CRC, non-cancer causes of death should not be ignored. For patients with cancer, we should not only focus on anti-tumor therapies but also pay attention to the occurrence of other risks to prevent and manage them in advance.

## Introduction

Colorectal cancer (CRC) is the third leading primary malignancy in both men and women (1). The incidence and mortality rates of CRC varied widely, with distinct gradients across human development index (HDI) levels. In countries with the highest HDI level, including the United States (US), both incidence and mortality rates decreased in recent years (2). In 2020, 147,950 Americans are estimated to be diagnosed with CRC, and 53,200 of them will die from it (1).

With the development of anti-tumor therapies, there has been a decline in mortality rates related to cancer in recent years (3, 4). Hence, we have been increasingly concerned about the non-cancer causes of death as the survival time goes on. Previous studies have shown that heart diseases, emergencies, and chronic diseases are among the leading causes of death in patients with cancer. In the general population, the mortality rate from heart diseases even surpassed that of cancer (1, 5, 6).

Few existing studies have concentrated on the causes of death from CRC, especially for non-cancer factors. However, with the pursuit of survival time and quality, the deaths caused by non-cancer factors should be taken seriously. The study of non-cancer mortality factors can avoid underestimating the risk of death of patients with cancer. Therefore, early intervention and active treatment can be carried out. The present study focused on the standardized mortality ratios (SMRs) for each cause of death and on the SMRs in each subgroup with different lengths of survival times.

## Materials and Methods

### Data Source

We gained data from the Surveillance, Epidemiology, and End Results (SEER) program, which includes ~28% of the general US population, and used the SEER*Stat software 8.3.8 to access the database SEER 18 registries, Nov 2018 submission (2000–2016), for SMRs. The database consists of public-use data, and this study did not require approval or a declaration of local ethics.

### Study Population

We included patients who were diagnosed with CRC between 2000 and 2016 in the US. Only malignant behavior and the first primary malignancies were selected. The cases diagnosed only through death certificate and autopsy were excluded. Based on the calculation of the SMR, patients with unknown survival status, follow-up time, and reasons of death were excluded. We also excluded patients of unknown age based on diagnosis, sex, and race.

### SMR

For patients with CRC from the SEER database, we measured the number of deaths in different variables. This study focused on the non-cancer causes of death in patients with CRC. Under diseases of the respiratory system, we included pneumonia, influenza, chronic obstructive pulmonary disease, and its allied conditions. Under diseases of the digestive system, we included stomach and duodenal ulcers, chronic liver disease, and cirrhosis. Under diseases of the arteries, we included heart diseases; hypertension; cerebrovascular diseases; atherosclerosis; aortic aneurysm and dissection; and other diseases of the arteries, arterioles, or capillaries. Under infectious diseases, we included tuberculosis, syphilis, septicemia, and other infectious diseases. We also included other non-cancer diseases and cancer-related diseases.

First, reported the percentages of patients with CRC dying from different causes in a specific subgroup at each follow-up stage. Then, we calculated SMRs with 95% confidence intervals for each cause of death after CRC diagnosis. SMRs are defined as the observed-to-expected ratios; the observed population included patients with CRC who were diagnosed between 2000 and 2016 in the US, while the expected population included the general population who were diagnosed between 1975 and 2016 in the US, which was collected from the SEER database. Because of the high mortality rate from cardiovascular diseases (heart diseases; hypertension; cerebrovascular diseases; atherosclerosis; aortic aneurysm/dissection; and other diseases of the arteries, arterioles, or capillaries), we have studied the causes of death in this area alone.

### Statistical Analysis

By using the SEER*Stat software 8.3.8 (https://seer.cancer.gov/seerstat/software/), we calculated SMRs with 95% confidence intervals. A significantly increased risk is defined as a higher number of deaths observed after CRC diagnosis than the expected number in the general population. *P*-value < 0.05 (two sided) was considered to be statistically significant.

## Results

### Baseline Characteristics

We included a total of 475,771 patients diagnosed with CRC from the SEER database, with more than half of the patients (54.6%) aging >64 years, 33.1% aging 50–64 years, and 12.3% aging 0–49 years. The proportion of men (51.6%) and women (48.4%) was similar. Most patients were white (79.4%). Of the included patients, 39.8% were diagnosed with localized and 33.7% were regional CRC. Meanwhile, 230,841 (48.5%) died during the follow-up period, with the mean age at death of 74.08 years. The largest number of deaths occurred during the 1- to 5-year follow-up period (47.8%), followed by <1 year (27.4%) after follow-up. Most patients performed cancer-directed surgery, due to which there was a reduction in mortality compared to non-surgery patients (44.2% vs. 77.5%). Table 1 lists the basic characteristics of the included patients, the number of deaths at different follow-up periods, and the mean age at death.

**Table 1:** Baseline characteristics of all patients with CRC and patients who died according to the time of death after diagnosis.

**Timing of deaths after diagnosis**											
		**All Deaths**		**<1year**		**1-5years**		**5-10years**		**>10years**	
**Characteristic**	**Total No. of Patients**	**No. of Patients (%)**	**Mean Age at Death, y**	**No. of Patients (%)**	**Mean Age at Death, y**	**No. of Patients (%)**	**Mean Age at Death, y**	**No. of Patients (%)**	**Mean Age at Death, y**	**No. of Patients (%)**	**Mean Age at Death, y**
Overall	475,771	230,841 (100)	74.08	63,354 (27.4)	73.21	110,351 (47.8)	71.59	42,266 (18.31)	78.54	14,870 (6.44)	83.55
**Age at diagnosis, y**											
0–49	58,604	19,159 (100)	45.86	4,315 (22.52)	43.17	11,600 (60.55)	45.3	2,674 (13.96)	50.37	570 (2.98)	56.34
50–64	157,352	55,609 (100)	61.54	13,372 (24.05)	58.93	30,144 (54.21)	60.59	9,113 (16.39)	65.29	2,980 (5.36)	71.28
>64	259,815	156,073 (100)	82.01	45,667 (29.26)	80.23	68,607 (43.96)	80.86	30,479 (19.53)	84.96	11,320 (7.25)	88.15
**Sex**											
Male	245,572	119,232 (100)	72.07	31,316 (26.26)	70.73	58,268 (48.87)	69.87	22,201 (18.62)	76.56	7,447 (6.25)	81.53
Female	230,199	111,609 (100)	76.22	32,038 (28.71)	75.64	52,083 (46.67)	73.51	20,065 (17.98)	80.72	7,423 (6.65)	85.57
**Race**											
White	377,946	185,982 (100)	75	50,520 (27.16)	74.03	87,524 (47.06)	72.54	35,340 (19.00)	79.24	12,598 (6.77)	84.02
Black	56,966	28,895 (100)	69.41	8,622 (29.84)	69.22	14,597 (50.52)	67.21	4,270 (14.78)	73.84	1,406 (4.87)	79.85
Other	40,859	15,964 (100)	71.83	4,212 (26.38)	71.58	8,230 (51.55)	69.24	2,656 (16.64)	76.65	866 (5.42)	82.79
**Disease stage**											
Localized	189,149	67,198 (100)	79.72	9,614 (14.31)	78.5	28,539 (42.47)	77.94	20,605 (30.66)	80.91	8,440 (12.56)	84.25
Regional	160,446	77,902 (100)	75.03	14,169 (18.19)	75.69	40,669 (52.21)	72.62	17,448 (22.40)	77.49	5,616 (7.21)	83.12
Distant	84,724	71,078 (100)	66.76	31,981 (44.99)	69.07	35,717 (50.25)	64.39	2,961 (4.17)	68.78	419 (0.59)	77.95
**Tumor grade**											
Well differentiated: Grade 1	44,955	16,186 (100)	76.49	2,987 (18.45)	74.85	7,424 (45.87)	74.03	4,126 (25.49)	79.22	1,649 (10.19)	83.64
Moderately differentiated: Grade 2	283,628	13,1970 (100)	74.69	28,154 (21.33)	73.9	66,692 (50.54)	72.12	27,594 (20.91)	78.59	9,530 (7.22)	83.76
Poorly differentiated: Grade 3	69,922	42,357 (100)	72.21	14,558 (34.37)	71.07	20,093 (47.44)	69.82	5,764 (13.61)	79.32	1,942 (4.58)	84.41
Undifferentiated, anaplastic: Grade 4	7,962	4,224 (100)	70.76	1,867 (44.20)	70.89	1,907 (45.15)	68.31	383 (9.07)	79.68	67 (1.59)	85.68
**Cancer-directed surgery**											
Yes	411,833	182,053 (100)	74.63	36,380 (19.98)	73.33	90,843 (49.9)	71.94	40,413 (22.20)	78.63	14,417 (7.92)	83.62
No	11,126	8,627 (100)	77.43	4,393 (50.92)	77.84	3,519 (40.79)	76.52	530 (6.14)	78.49	185 (2.14)	81.76
**Chemotherapy**											
Yes	181,364	91,445 (100)	66.47	21,405 (23.41)	65.59	52,768 (57.7)	64.68	13,439 (14.70)	71.2	3,833 (4.19)	79.39
**Radiotherapy**											
Yes	67,109	31,525 (100)	68.05	6,835 (21.68)	67.87	17,230 (54.66)	66.02	5,793 (18.38)	71.27	1,667 (5.29)	78.53

### Non-cancer Causes of Death Within 1 Year After CRC Diagnosis

**Figure 1:**
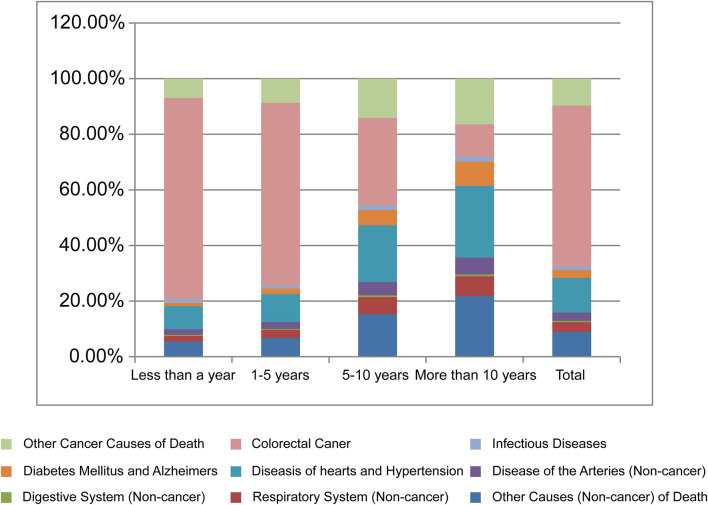
Causes of death in each latency period following CRC diagnosis.

A total of 63,354 patients with CRC died within 1 year after diagnosis, 50,111 (79.1%) of whom died from malignant tumors, 45,894 (72.4%) of whom died from CRC, and 13,087 (20.7%) of whom died from non-cancer causes. The most common non-cancer cause of death in this period was diseases of the heart (5,074; 8.0%), followed by cerebrovascular diseases and chronic obstructive pulmonary disease (COPD) (Table 2, Figure 1). Patients with CRC were more likely to die from sepsis and other infections during the 1-year follow-up period, with SMRs of 3.39 (95% CI, 3.11–3.69) and 3.10 (95% CI, 2.74–3.50), respectively (Table 2).

**Table 2:** Standardized mortality ratios for each cause of death after CRC diagnosis.

**Timing of deaths after diagnosis**										
**Cause of death**	**<1 years**		**1-5 years**		**5-10 years**		**>10 years**		**Total**	
**Selected events**	**No. observed**	**SMR (95%CI)**	**No. observedved**	**SMR (95%CI)**	**No. observedved**	**SMR (95%CI)**	**No. observedved**	**SMR (95%CI)**	**No. observedved**	**SMR (95%CI)**
All cause of death	63,354	5.79^#^ (5.74–5.83)	110,351	2.99^#^ (2.97–3)	42,266	1.58^#^ (1.57–1.6)	14,870	1.29^#^ (1.27–1.31)	230,841	2.68^#^ (2.67–2.69)
All malignant cancers	50,111	20.86^#^ (20.68–21.04)	81,892	10.30^#^ (10.23–10.37)	19,084	3.51^#^ (3.47–3.57)	4,090	1.85^#^ (1.8–1.91)	155,177	8.62^#^ (8.58–8.67)
Colon and rectum	45,894	195.05^#^ (193.27–196.85)	72,659	94.85^#^ (94.16–95.54)	13,312	26.25^#^ (25.8–26.7)	1,737	8.68^#^ (8.28–9.1)	133,602	78.20^#^ (77.78–78.62)
*In situ*, benign, or unknown behavior neoplasms	156	2.30^#^ (1.96–2.69)	327	1.40^#^ (1.25–1.56)	206	1.18^#^ (1.03–1.36)	84	1.1 (0.88–1.36)	773	1.40^#^ (1.31–1.5)
Tuberculosis	5	1.7 (0.55–3.96)	10	1.08 (0.52–1.99)	6	1.03 (0.38–2.25)	6	2.72 (1–5.92)	27	1.34 (0.88–1.94)
Syphilis	0	0 (0–21.95)	0	0 (0–6.82)	1	2.69 (0.07–14.98)	0	0 (0–23.48)	1	0.81 (0.02–4.5)
Septicemia	542	3.39^#^ (3.11–3.69)	752	1.40^#^ (1.3–1.5)	456	1.17^#^ (1.07–1.29)	180	1.08 (0.93–1.25)	1,930	1.54^#^ (1.47–1.61)
Other infectious diseases	267	3.10^#^ (2.74–3.5)	320	1.1 (0.98–1.23)	200	0.98 (0.85–1.12)	77	0.95 (0.75–1.18)	864	1.30^#^ (1.22–1.39)
Diabetes mellitus	491	1.51^#^ (1.38–1.65)	1,215	1.13^#^ (1.07–1.19)	947	1.27^#^ (1.19–1.36)	437	1.42^#^ (1.29–1.56)	3,090	1.26^#^ (1.22–1.3)
Alzheimer's	248	0.59^#^ (0.52–0.67)	1,029	0.67^#^ (0.63–0.71)	1396	1.08^#^ (1.02–1.14)	857	1.34^#^ (1.25–1.43)	3530	0.91^#^ (0.88–0.94)
Diseases of hearts	5074	1.63^#^ (1.58–1.67)	10579	1.02^#^ (1–1.04)	8182	1.13^#^ (1.1–1.15)	3635	1.18^#^ (1.14–1.22)	27470	1.15^#^ (1.14–1.17)
Hypertension without heart disease	196	1.56^#^ (1.35–1.8)	455	1.03 (0.94–1.13)	431	1.24^#^ (1.12–1.36)	204	1.28^#^ (1.11–1.47)	1286	1.20^#^ (1.13–1.26)
Cerebrovascular diseases	1004	1.39^#^ (1.3–1.48)	2210	0.93^#^ (0.89–0.97)	1677	1.01 (0.96–1.06)	739	1.04 (0.97–1.12)	5630	1.03^#^ (1–1.06)
Atherosclerosis	85	1.64^#^ (1.31–2.03)	176	1.09 (0.93–1.26)	96	0.95 (0.77–1.17)	56	1.47^#^ (1.11–1.9)	413	1.17^#^ (1.06–1.29)
Aortic aneurysm and dissection	79	1.34^#^ (1.06–1.67)	157	0.84^#^ (0.71–0.98)	112	0.96 (0.79–1.15)	48	1.09 (0.8–1.44)	396	0.97 (0.88–1.07)
Other diseases of arteries, arterioles, capillaries	78	1.68^#^ (1.33–2.1)	142	0.92 (0.78–1.09)	129	1.19 (0.99–1.41)	56	1.21 (0.92–1.58)	405	1.14^#^ (1.03–1.26)
Pneumonia and influenza	428	1.44^#^ (1.3–1.58)	888	0.90^#^ (0.84–0.96)	802	1.15^#^ (1.07–1.23)	334	1.14^#^ (1.02–1.27)	2452	1.08^#^ (1.03–1.12)
Chronic obstructive pulmonary disease and allied condition	882	1.36^#^ (1.28–1.46)	2267	1.02 (0.98–1.07)	1770	1.09^#^ (1.04–1.14)	726	1.04 (0.96–1.11)	5645	1.09^#^ (1.06–1.12)
Stomach and duodenal ulcers	37	2.28^#^ (1.6–3.14)	52	1.01 (0.75–1.32)	43	1.29 (0.93–1.73)	11	0.81 (0.41–1.46)	143	1.25^#^ (1.05–1.47)
Chronic liver disease and cirrhosis	227	2.53^#^ (2.21–2.88)	351	1.22^#^ (1.1–1.35)	243	1.33^#^ (1.17–1.51)	90	1.27^#^ (1.02–1.56)	911	1.44^#^ (1.35–1.54)
Nephritis, nephrotic syndrome and nephrosis	386	1.74^#^ (1.57–1.93)	830	1.09^#^ (1.02–1.16)	701	1.24^#^ (1.15–1.33)	282	1.16^#^ (1.03–1.3)	2199	1.23^#^ (1.17–1.28)
Complications of pregnancy, childbirth, puerperium	7	34.78^#^ (13.98–71.67)	15	27.43^#^ (15.35–45.23)	2	8.45^#^ (1.02–30.52)	2	31.38^#^ (3.8–113.34)	26	24.79^#^ (16.2–36.33)
Congenital anomalies	13	1.47 (0.79–2.52)	19	0.68 (0.41–1.06)	15	0.85 (0.47–1.4)	3	0.43 (0.09–1.26)	50	0.81 (0.6–1.07)
Certain conditions originating in perinatal period	0	0 (0–110.05)	1	9.48 (0.24–52.8)	1	15.67 (0.4–87.33)	0	0 (0–154.93)	2	8.82^#^ (1.07–31.87)
Symptoms, signs and ill-defined conditions	223	1.70^#^ (1.49–1.94)	488	1.04 (0.95–1.14)	387	1.04 (0.94–1.14)	152	0.96 (0.81–1.12)	1250	1.11^#^ (1.04–1.17)
Accidents and adverse effects	310	1.1 (0.98–1.23)	869	0.91^#^ (0.85–0.97)	666	0.96 (0.88–1.03)	328	1.07 (0.96–1.19)	2173	0.97 (0.93–1.01)
Suicide and self-inflicted injury	116	1.96^#^ (1.62–2.35)	266	1.41^#^ (1.24–1.59)	142	1.20^#^ (1.01–1.41)	49	1.07 (0.79–1.41)	573	1.39^#^ (1.28–1.51)
Homicide and legal intervention	12	0.99 (0.51–1.73)	39	1.12 (0.79–1.52)	22	1.19 (0.75–1.8)	8	1.3 (0.56–2.56)	81	1.13 (0.9–1.4)
Other cause of death	2377	1.49^#^ (1.43–1.55)	5002	0.88^#^ (0.86–0.91)	4549	1 (0.97–1.03)	2416	1.14^#^ (1.1–1.19)	14344	1.03^#^ (1.01–1.05)

# *P < 0.05*.

In general, the causes of death in the first year after CRC diagnosis for a specific subgroup generally follow similar trends to the total population of CRC, with the majority of deaths due to cardiac diseases (Supplementary Tables 2–15, 20).

Patients after CRC diagnosis had a higher risk of infectious diseases, such as septicemia within the first year, regardless of age. Interestingly, we found that deaths from suicide and self-inflicted injury increased significantly over the age of 50 compared to the general population (Supplementary Tables 1–3). Severe complications of pregnancy and childbirth were more common in female patients with CRC (Supplementary Table 4). White people diagnosed with CRC had a significantly higher rate of death from digestive complications than the general population, while black people did not (Supplementary Tables 6, 7).

In patients with distant-stage, undifferentiated, and adenocarcinoma CRC, the rate of death due to non-cancer causes was higher than that of the general population. Non-cancer causes of death were mainly stomach and duodenal ulcers and septicemia (see Supplementary Tables 9–15, 20). In comparison with non-surgical patients, the risk of death from non-cancer causes was significantly reduced in patients who underwent surgery (Supplementary Tables 18, 19).

### Non-cancer Causes of Death Within 1 to 5 Years After CRC Diagnosis

A total of 110,351 patients died within 1–5 years after CRC diagnosis, of whom 72,659 (65.8%) died from CRC, and 28,132 (25.5%) died from non-cancer causes. The most common non-cancer causes of death in this period were diseases of the heart (10,579; 9.6%), followed by COPD (2,267; 2.1%) and cerebrovascular diseases (2,210; 2.0%) (Table 2, Figure 1). In comparison with the general US population, there was no significant increase in the number of non-cancer deaths in this time period after cancer diagnosis (Table 2).

Within 1–5 years after CRC diagnosis in specific subgroups, cardiac diseases remained the most common non-cancer causes of death. Additionally, the mortality from cardiac diseases has increased when it was compared within 1 year after CRC diagnosis (Supplementary Tables 2–15).

In demographic-related subgroups, patients with CRC had a significantly higher risk of death from septicemia, especially in patients aging 0–49 years (Supplementary Table 1). Similarly, deaths from nephritis and the nephrotic syndrome were higher among patients aging 0–64 years and in black patients compared with the general population (Supplementary Tables 1, 2, 7). Male patients over the age of 65 have a higher risk of death from suicide and self-inflicted injury than the general population (Supplementary Tables 3, 5), while white female patients have a higher risk of death from suicide and self-inflicted injury (Supplementary Tables 4, 6).

In patients with distant-stage CRC and who received chemotherapy and radiotherapy, the number of death from septicemia was more than the general US population (Supplementary Tables 9, 16, 17). Suicide deaths were also more common in patients with distant metastases and in patients who had surgery (Supplementary Tables 9, 18).

### Non-cancer Causes of Death Within 5 to 10 Years After CRC Diagnosis

A total of 42,266 patients with CRC died during this follow-up period, of whom 13,312 (31.5%) died from CRC, and 22,976 (54.4%) died from non-cancer causes. The most common non-cancer causes of death in this period were diseases of the heart (8,182; 19.6%), followed by COPD (1,770; 4.2%) and cerebrovascular diseases (1,677; 4.0%) (Table 2, Figure 1). Patients with CRC were more likely to die from diabetes mellitus and hypertension during this time period, with SMRs of 1.27 (95% CI, 1.19–1.36) and 1.24 (95% CI, 1.12–1.36), respectively (Table 2).

In demographic-related subgroups, SMRs were mainly higher for several non-cancer causes of death, including nephritis, chronic liver disease, diabetes mellitus, and septicemia within 5–10 years. Patients with CRC were more likely to die from nephritis, especially in black patients aging 0–64 years (Supplementary Tables 1, 2, 7, 12, 19). Similarly, deaths from the chronic liver disease were higher among white female patients compared with the general population (Supplementary Tables 4, 6). We found that the risk of death due to diabetes mellitus was generally higher at this period, regardless of age and gender (Supplementary Tables 1–5). Caucasians between the ages of 50 and 64 have a higher risk of death from septicemia compared with the general US population (Supplementary Tables 2, 6).

In tumor-related subgroups, the risk of death from stomach and duodenal ulcers in regional-stage CRC was significantly higher than that of the general population, with an SMR of 2.19 (95% CI, 1.45–3.19) (Supplementary Table 10). In patients with CRC who had no surgery, the cause of death from nephritis was significantly higher, with an SMR of 3.30 (95% CI, 1.98–5.15) (Supplementary Table 19).

### Non-cancer Causes of Death >10 Years After CRC Diagnosis

A total of 14,870 patients with CRC died >10 years after cancer diagnosis, of whom 4,090 (27.5%) died from malignant tumors, 1,737 (11.7%) died from CRC, and 10,696 (71.9%) died from non-cancer causes. The most common non-cancer causes of death in this period were diseases of the heart (3,635; 24.4%), followed by Alzheimer's disease (857; 5.8%) and cerebrovascular diseases (739; 5.0%) (Table 2, Figure 1). Patients with CRC had a significantly higher risk of death from atherosclerosis and diabetes mellitus during the time period after cancer diagnosis, with SMRs of 1.47 (95% CI, 1.11–1.9) and 1.42 (95% CI, 1.29–1.56), respectively (Table 2).

In demographic-related subgroups, the risk of death from Alzheimer's disease was significantly higher during the time period, especially among patients who were diagnosed between ages >49 years and female blacks (Supplementary Tables 2–4). In most subgroups, we found that diabetes mellitus had a significantly higher risk in patients with CRC than the general US population, except for patients with CRC aging 0–49 years (Supplementary Tables 1–8). In tumor-related subgroups, the risks of death from diabetes mellitus, Alzheimer's disease, and cardiac diseases were higher than that of the general US population (Supplementary Tables 9–20).

### Risk of Death From Cardiovascular Disease After CRC Diagnosis

**Figure 2:**
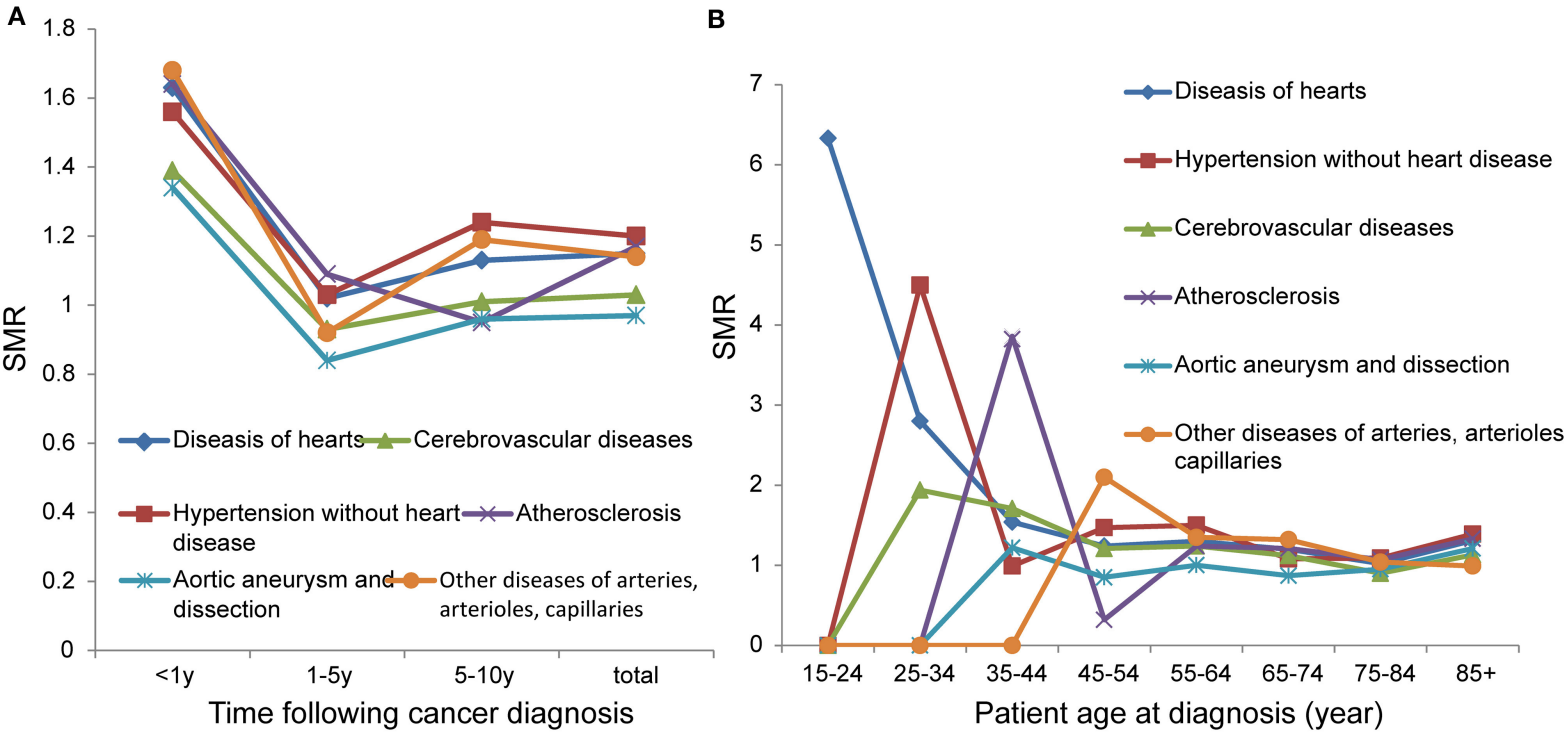
SMRs of cardiovascular disease at different latency periods **(A)**; SMRs of cardiovascular disease at different age **(B)**.

In the total population we studied, the number of deaths due to cardiovascular disease was 35,600, accounting for 15.4% of total deaths (Table 2). Overall, patients with CRC had a higher risk of death from cardiovascular diseases (except cerebrovascular diseases, aortic aneurysm, and dissection) than the general population. During the 1–5-year follow-up period, the risk of death from cardiovascular diseases was not higher than that of the general population, and some were even lower. In addition, patients with CRC had the highest risk of death from cardiovascular diseases within 1 year of diagnosis, with an SMR reaching more than 1.3 (Figure 2A). In young adults (0–54 years), the risk of death from diseases of the heart decreases with age (15–24: SMR 6.33; 25–34: SMR 2.8; 35–44: SMR 1.54; 45–54: SMR 1.24). In those older than 54 years, the SMRs of cardiovascular diseases were maintained at a level >1 (Figure 2B).

## Discussion

Colorectal cancer is one of the most common malignant tumors in the world. As the main cause of death in patients with CRC is their cancer, there have been a lot of reports on the study of therapeutic drugs and surgical methods in recent years (7–9). The survival time of patients with CRC is longer due to improved anti-tumor treatments (10). Hence, the increasing number of deaths is not caused by cancer but by other non-cancer factors. However, this issue has not been of general concern to researchers, so this study focuses on non-cancer causes of death in patients with CRC.

In this study, the majority of patients with CRC died from CRC within the 5-year follow-up period. However, when the follow-up period was over 5 years, we found that the proportion of deaths due to tumors decreased, while the proportion of deaths due to non-cancer causes increased. Deaths from non-cancer causes accounted for 71.9% of all deaths over the 10-year follow-up period. Non-cancer factors are mainly cardiovascular diseases, diabetes mellitus, infectious diseases, and diseases of the respiratory system (Figure 1).

Oxaliplatin and fluorouracil are commonly used to treat advanced and resected CRC. The main adverse effect of oxaliplatin is neurotoxicity, but its cardiotoxicity should also be taken into account, especially in conjunction with capecitabine (11, 12). Within the first 2 years since CRC diagnosis, exposure to capecitabine and 5-fluorouracil increased the risk of cardiovascular diseases, including stroke and myocardial infarction (13). The cardiotoxicity caused by capecitabine includes angina-like chest pain, cardiac arrhythmias, hypertension, hypotension, heart failure, cardiac ischemia, myocardial infarction, cardiogenic shock, or sudden death (14, 15). The majority of events are observed during the first treatment cycle, which usually results in discontinuation of the treatment (16).

Bevacizumab, a monoclonal antibody, is also often used in the treatment of CRC. It is sometimes involved in thromboembolic complications, in ischemic disease of the heart, and in hypertension (17, 18). Worryingly, the use of bevacizumab increases the risk of fluorouracil causing cardiac ischemia (19). Similarly, panitumumab, a commonly targeted drug for CRC, has been reported to increase the risk of arrhythmia in patients (19).

In recent years, immune checkpoint inhibitors (ICIs) have also begun to be used in the treatment of CRC, greatly improving the survival rates. Meanwhile, the cardiac toxicities caused by it should not be ignored (20). Cardiac toxicities from ICIs include myocarditis, pericarditis, arrhythmias and heart block, and new-onset heart failure, although their incidence is >1% (21).

Because of the application of these anti-tumor drugs, patients with CRC have a higher death rate from cardiovascular diseases than the general population, especially in the first few years of treatment. Interestingly, this study found that the risk of death from cardiovascular diseases was not significantly higher among patients with CRC who underwent chemotherapy than in the general US population. This may be explained by the limited accuracy of recording in the SEER database of the type of chemotherapy delivered.

Cancer and diabetes are also inextricably linked. Because of common factors such as diet and obesity, patients with diabetes are more likely to develop CRC, and the incidence of diabetes among patients with CRC is higher than that of the general US population (22, 23). A large cohort study showed a significantly increased risk of developing diabetes in patients with CRC without metastasis, regardless of whether they received chemotherapy or not (24). Conversely, another study in China showed that chemotherapy regimens based on 5-fluorouracil induced diabetes in patients with CRC (23). This study found that death from diabetes increased with follow-up time, accounting for 28.9% of deaths over 10 years. Over a follow-up period of more than 5 years, patients with CRC did have a significant increase in the risk of dying from diabetes compared to the general US population.

In clinical practice, hemocytopenia caused by anti-tumor therapies is a very common phenomenon in patients (17, 25–27). The reduction of neutrophils is the major independent risk factor for septicemia (28). A retrospective study suggested that cancer patients with sepsis and bacteremia had a higher risk of death. Patients with cancer had 2.320 (95% CI, 1.225–4.395, *p* = 0.010) odds of dying compared with patients without cancer in the setting of septicemia (29). Simultaneously, we found that the metastatic processes may also impair the immune system through invasion and mechanical obstruction, leading to an increased risk of infection in patients with cancer (30, 31). In this study, patients with CRC had a significantly higher risk of death from septicemia than the general population, especially among patients dying within 1 year. In patients with CRC who underwent chemoradiotherapy, the risk of death from sepsis was indeed increased compared to the total number of patients.

The incidence of diseases of the respiratory system has been noted to be higher among patients with cancer (32). Lung infections caused by neutropenia are the most common. A meta-analysis showed that there was also a significant increase in high-grade pneumonia caused by ICIs compared to chemotherapy (33). Moreover, in comparison with non-cancer patients, the prevalence of COPD in patients with cancer was significantly higher (OR 1.21, 95% CI, 1.06–1.37) (30). The same result was shown in this study, but there was no significant increase in the risk of death from disease of the respiratory system among patients with CRC receiving chemotherapy.

In the present study, we found that, during the early follow-up period, patients with CRC who underwent surgery had lower SMRs than patients who did not undergo surgery. Extensive surgery, especially in the gastrointestinal tract (such as Hartmann's operation), increases the risk of infection in patients with cancer (34). Similarly, other serious complications after the surgery, such as massive bleeding and venous thrombosis, also increase the risk of non-cancer death in patients with cancer (35). However, the data do show the opposite result. Therefore, based on our clinical experience, we hypothesize that patients with potentially operable tumors have relatively few underlying diseases and better underlying health than patients who did not undergo surgery, resulting in lower non-cancer deaths.

To our knowledge, the purposes of the study are, first, to investigate non-cancer causes of death in CRC and, second, to divide them into different follow-up periods and subgroups. The main limitation of this study is that it is a retrospective study. And we did our best to reduce the bias. First, to reduce the selection bias, we designed strict screening criteria: only the first primary CRC proved by pathology was included to reduce the bias caused by other metastases or subsequent CRC. Second, to reduce the confounding bias, we used SMRs to control the differences in age, sex, and race rather than direct mortality rates. Data on treatment were missing in most patients, and there is no way to know the relevant information, such as gene mutations in patients with CRC and differences in lifestyle between the two groups, which prohibited us from adjusting these features.

## Conclusions

In conclusion, the proportion of deaths from non-cancer causes increased with the extension of survival time. Cardiovascular disease accounts for the vast majority of deaths. In addition, the causes of death from diabetes, diseases of the respiratory system, and infectious diseases are common in patients with CRC. Therefore, for patients with cancer, we should not only focus on anti-tumor therapies but also pay attention to the occurrence of other risks to prevent and manage them in advance.

## Data Availability Statement

The original contributions presented in the study are included in the article/Supplementary Material, further inquiries can be directed to the corresponding author/s.

## Author Contributions

YF conceived and designed the study, performed the study, analyzed the data, prepared figures and/or tables, and authored or reviewed drafts of the paper. HJ and KG conceived and designed the study, performed the study, and analyzed the data. HW and SR performed the study and authored or reviewed drafts of the paper. CC conceived and designed the study, performed the study, authored or reviewed drafts of the paper, and approved the final draft. All authors contributed to the article and approved the submitted version.

## Conflict of Interest

The authors declare that the research was conducted in the absence of any commercial or financial relationships that could be construed as a potential conflict of interest.
