# Supplementary material for: Causes of Death After Colorectal Cancer Diagnosis: A Population-Based Study
Source: Front Oncol. 2021 Mar 30;11:647179. doi: 10.3389/fonc.2021.647179 (PMC8042257; doi:10.3389/fonc.2021.647179)
Supplement: Supplementary file 1 [file Data_Sheet_1.docx]

Supplementary Table 1. Standardized-mortality ratios (SMRs) for each cause of death following CRC diagnosis in patients aged younger than 49 years.

| Timing of Deaths After Diagnosis | | | | | | | | | | |  |  |  |  |
| --- | --- | --- | --- | --- | --- | --- | --- | --- | --- | --- | --- | --- | --- | --- |
| Cause of death | <1year | | 1-5years | | 5-10years | | >10years | | Total | |  |  |  |  |
| Selected Events | No. Observed | SMR(95%CI) | No. Observed | SMR(95%CI) | No. Observed | SMR(95%CI) | No. Observed | SMR(95%CI) | No. Observed | SMR(95%CI) |  |  |  |  |
| All cause of death | 4315 | 35.56#(34.51-36.64) | 11600 | 24.80#(24.35-25.25) | 2674 | 6.33#(6.1-6.58) | 570 | 2.16#(1.98-2.34) | 19159 | 15.02#(14.81-15.23) |  |  |  |  |
| All malignant cancers | 4018 | 153.96#(149.24-158.8) | 10922 | 96.99#(95.18-98.83) | 2244 | 19.08#(18.3-19.89) | 339 | 4.16#(3.73-4.63) | 17523 | 51.88#(51.12-52.66) |  |  |  |  |
| Colon and Rectum | 3703 | 1405.60#(1360.69-1451.62) | 10303 | 914.36#(896.79-932.19) | 1998 | 176.04#(168.4-183.93) | 222 | 29.96#(26.15-34.17)17) | 16226 | 496.77#(489.16-504.48) |  |  |  |  |
| In situ, benign, or unknown behavior neoplasms | 11 | 24.14#(12.05-43.2) | 24 | 13.58#(8.7-20.2) | 10 | 6.06#(2.91-11.15) | 4 | 3.58(0.97-9.16) | 49 | 9.82#(7.26-12.98) |  |  |  |  |
| Tuberculosis | 0 | 0(0-68.1) | 0 | 0(0-18.77) | 0 | 0(0-24.1) | 1 | 11.83(0.3-65.91) | 1 | 2.05(0.05-11.41) |  |  |  |  |
| syphilis | 0 | 0(0-1307.44) | 0 | 0(0-336.42) | 0 | 0(0-389.67) | 0 | 0(0-671.12) | 0 | 0(0-128.31) |  |  |  |  |
| Septicemia | 13 | 8.97#(4.78-15.34) | 33 | 5.59#(3.84-7.84) | 9 | 1.54(0.7-2.93) | 8 | 2(0.86-3.93) | 63 | 3.66#(2.81-4.69) |  |  |  |  |
| Other infectious diseases | 23 | 4.38#(2.78-6.58) | 42 | 2.39#(1.72-3.23) | 13 | 1.05(0.56-1.8) | 14 | 2.38#(1.3-3.99) | 92 | 2.24#(1.81-2.75) |  |  |  |  |
| Diabetes Mellitus | 13 | 3.70#(1.97-6.32) | 23 | 1.59#(1.01-2.38) | 25 | 1.72#(1.12-2.54) | 11 | 1.1(0.55-1.97) | 72 | 1.69#(1.33-2.13) |  |  |  |  |
| Alzheimers | 0 | 0(0-113.88) | 0 | 0(0-19.35) | 1 | 2.72(0.07-15.14) | 0 | 0(0-6.87) | 1 | 0.89(0.02-4.94) |  |  |  |  |
| Diseasis of hearts | 64 | 2.90#(2.24-3.71) | 134 | 1.48#(1.24-1.76) | 102 | 1.18(0.96-1.43) | 63 | 1.12(0.86-1.43) | 363 | 1.42#(1.28-1.58) |  |  |  |  |
| Hypertension without heart disease | 1 | 1.28(0.03-7.13) | 6 | 1.81(0.66-3.93) | 4 | 1.16(0.32-2.98) | 6 | 2.43(0.89-5.29) | 17 | 1.7(0.99-2.72) |  |  |  |  |
| Cerebrovascular diseases | 8 | 2.14(0.92-4.22) | 26 | 1.76#(1.15-2.57) | 17 | 1.25(0.73-2.01) | 10 | 1.14(0.55-2.1) | 61 | 1.49#(1.14-1.92) |  |  |  |  |
| Atherosclerosis | 1 | 13.72(0.35-76.47) | 0 | 0(0-11.48) | 0 | 0(0-10.58) | 0 | 0(0-14.53) | 1 | 1(0.03-5.59) |  |  |  |  |
| Aortic aneurysm and dissection | 2 | 4.04(0.49-14.58) | 0 | 0(0-2) | 2 | 1.25(0.15-4.5) | 1 | 0.99(0.03-5.52) | 5 | 1.01(0.33-2.35) |  |  |  |  |
| Other diseases of arteries, arterioles, capillaries | 1 | 4.7(0.12-26.2) | 2 | 2.3(0.28-8.32) | 0 | 0(0-4.09) | 3 | 4.3(0.89-12.56) | 6 | 2.24(0.82-4.87) |  |  |  |  |
| Pneumonia and influenza | 4 | 2.82(0.77-7.22) | 6 | 1.08(0.4-2.35) | 3 | 0.58(0.12-1.69) | 3 | 0.88(0.18-2.56) | 16 | 1.03(0.59-1.67) |  |  |  |  |
| Chronic obstructive pulmonary disease and allied cond | 7 | 3.66#(1.47-7.55) | 16 | 1.72(0.98-2.79) | 19 | 1.56(0.94-2.43) | 9 | 0.84(0.38-1.59) | 51 | 1.49#(1.11-1.96) |  |  |  |  |
| Stomach and duodenal ulcers | 1 | 6.3(0.16-35.09) | 0 | 0(0-5.86) | 2 | 3.45(0.42-12.46) | 0 | 0(0-10) | 3 | 1.73(0.36-5.05) |  |  |  |  |
| Chronic liver disease and cirrhosis | 15 | 2.96#(1.66-4.88) | 29 | 1.42(0.95-2.04) | 31 | 1.67#(1.13-2.37) | 12 | 1.15(0.59-2.01) | 87 | 1.60#(1.28-1.97) |  |  |  |  |
| Nephritis, Nephrotic Syndrome and Nephrosis | 5 | 3.82#(1.24-8.92) | 18 | 3.38#(2-5.34) | 20 | 3.78#(2.31-5.83) | 8 | 2.13(0.92-4.19) | 51 | 3.25#(2.42-4.27) |  |  |  |  |
| Complications of pregnancy, childbirth, puerperium | 5 | 37.15#(12.06-86.69) | 11 | 26.54#(13.25-47.49) | 2 | 8.85#(1.07-31.97) | 2 | 32.24#(3.9-116.45) | 20 | 23.89#(14.59-36.9) |  |  |  |  |
| Congenital anomalies | 1 | 1.73(0.04-9.62) | 2 | 0.99(0.12-3.59) | 1 | 0.63(0.02-3.52) | 0 | 0(0-4.34) | 4 | 0.8(0.22-2.04) |  |  |  |  |
| Certain conditions originating in perinatal period | 0 | 0(0-643.39) | 1 | 55.96#(1.42-311.79) | 0 | 0(0-386.25) | 0 | 0(0-964.9) | 1 | 27.04(0.68-150.68) |  |  |  |  |
| Symptoms, Signs and Ill-Defined Conditions | 13 | 6.10#(3.25-10.43) | 34 | 4.82#(3.34-6.74) | 11 | 2.23#(1.11-3.99) | 4 | 1.59(0.43-4.06) | 62 | 3.73#(2.86-4.78) |  |  |  |  |
| Accidents and Adverse Effects | 23 | 1.22(0.77-1.83) | 64 | 1.03(0.79-1.32) | 42 | 0.99(0.71-1.34) | 21 | 1.11(0.69-1.7) | 150 | 1.05(0.89-1.24) |  |  |  |  |
| Suicide and Self-Inflicted Injury | 6 | 0.8(0.29-1.73) | 37 | 1.47#(1.03-2.03) | 24 | 1.37(0.87-2.03) | 10 | 1.3(0.62-2.39) | 77 | 1.33#(1.05-1.66) |  |  |  |  |
| Homicide and legal intervention | 1 | 0.32(0.01-1.79) | 12 | 1.37(0.71-2.38) | 6 | 1.29(0.47-2.82) | 1 | 0.6(0.02-3.34) | 20 | 1.1(0.67-1.7) |  |  |  |  |
| Other Cause of Death | 79 | 5.28#(4.18-6.58) | 158 | 2.78#(2.36-3.25) | 86 | 1.71#(1.37-2.11) | 40 | 1.27(0.91-1.73) | 363 | 2.37#(2.13-2.62) |  |  |  |  |

# P value less than .05

Supplementary Table 2. Standardized-mortality ratios (SMRs) for each cause of death following CRC diagnosis in patients aged 50-64 years.

| Timing of Deaths After Diagnosis | | | | | | | | | | |  |  |  |  |
| --- | --- | --- | --- | --- | --- | --- | --- | --- | --- | --- | --- | --- | --- | --- |
| Cause of death | <1years | | 1-5years | | 5-10years | | >10years | | Total | |  |  |  |  |
| Selected Events | No. Observed | SMR(95%CI) | No. Observed | SMR(95%CI) | No. Observed | SMR(95%CI) | No. Observed | SMR(95%CI) | No. Observed | SMR(95%CI) |  |  |  |  |
| All cause of death | 13372 | 12.83#(12.61-13.05) | 30144 | 7.56#(7.48-7.65) | 9113 | 2.59#(2.53-2.64) | 2980 | 1.41#(1.36-1.46) | 55609 | 5.21#(5.17-5.26) |  |  |  |  |
| All malignant cancers | 11731 | 33.70#(33.1-34.32) | 26476 | 19.43#(19.2-19.67) | 6319 | 5.23#(5.11-5.36) | 1351 | 2.01#(1.9-2.12) | 45877 | 12.78#(12.66-12.89) |  |  |  |  |
| Colon and Rectum | 10735 | 346.88#(340.35-353.51) | 24217 | 205.44#(202.86-208.04) | 4834 | 48.81#(47.44-50.2) | 624 | 11.81#(10.9-12.77) | 40410 | 134.38#(133.07-135.7) |  |  |  |  |
| In situ, benign, or unknown behavior neoplasms | 21 | 4.42#(2.74-6.76) | 60 | 3.10#(2.36-3.99) | 40 | 2.04#(1.46-2.78) | 19 | 1.38(0.83-2.15) | 140 | 2.43#(2.05-2.87) |  |  |  |  |
| Tuberculosis | 2 | 4.87(0.59-17.58) | 2 | 1.38(0.17-5) | 2 | 1.88(0.23-6.78) | 1 | 1.92(0.05-10.71) | 7 | 2.03(0.82-4.19) |  |  |  |  |
| syphilis | 0 | 0(0-155.46) | 0 | 0(0-41.51) | 0 | 0(0-45.82) | 0 | 0(0-78.21) | 0 | 0(0-15.35) |  |  |  |  |
| Septicemia | 92 | 6.04#(4.87-7.41) | 157 | 2.60#(2.21-3.04) | 85 | 1.50#(1.2-1.85) | 42 | 1.19(0.86-1.61) | 376 | 2.24#(2.02-2.48) |  |  |  |  |
| Other infectious diseases | 72 | 3.31#(2.59-4.16) | 86 | 1.22(0.98-1.51) | 49 | 1.05(0.78-1.39) | 20 | 0.96(0.59-1.49) | 227 | 1.42#(1.24-1.62) |  |  |  |  |
| Diabetes Mellitus | 78 | 1.91#(1.51-2.38) | 242 | 1.52#(1.34-1.73) | 212 | 1.51#(1.32-1.73) | 128 | 1.60#(1.33-1.9) | 660 | 1.57#(1.45-1.7) |  |  |  |  |
| Alzheimers | 1 | 0.43(0.01-2.37) | 9 | 0.68(0.31-1.3) | 31 | 1.28(0.87-1.81) | 50 | 1.46#(1.09-1.93) | 91 | 1.23(0.99-1.51) |  |  |  |  |
| Diseasis of hearts | 517 | 2.10#(1.93-2.29) | 1158 | 1.25#(1.17-1.32) | 949 | 1.19#(1.11-1.26) | 531 | 1.11#(1.02-1.21) | 3155 | 1.29#(1.24-1.33) |  |  |  |  |
| Hypertension without heart disease | 21 | 2.37#(1.46-3.62) | 57 | 1.63#(1.23-2.11) | 33 | 1(0.69-1.4) | 33 | 1.54#(1.06-2.16) | 144 | 1.46#(1.23-1.72) |  |  |  |  |
| Cerebrovascular diseases | 87 | 2.25#(1.8-2.77) | 185 | 1.23#(1.06-1.42) | 156 | 1.11(0.94-1.29) | 94 | 0.98(0.79-1.2) | 522 | 1.22#(1.12-1.33) |  |  |  |  |
| Atherosclerosis | 4 | 2.81(0.77-7.2) | 8 | 1.4(0.6-2.76) | 2 | 0.37(0.05-1.35) | 4 | 1.11(0.3-2.85) | 18 | 1.12(0.66-1.77) |  |  |  |  |
| Aortic aneurysm and dissection | 8 | 1.61(0.7-3.18) | 18 | 0.93(0.55-1.47) | 14 | 0.84(0.46-1.41) | 9 | 0.94(0.43-1.79) | 49 | 0.97(0.72-1.28) |  |  |  |  |
| Other diseases of arteries, arterioles, capillaries | 9 | 3.00#(1.37-5.7) | 14 | 1.13(0.62-1.9) | 16 | 1.32(0.75-2.14) | 11 | 1.39(0.69-2.48) | 50 | 1.41#(1.05-1.86) |  |  |  |  |
| Pneumonia and influenza | 26 | 1.98#(1.29-2.9) | 90 | 1.71#(1.38-2.11) | 70 | 1.36#(1.06-1.71) | 40 | 1.12(0.8-1.53) | 226 | 1.48#(1.29-1.68) |  |  |  |  |
| Chronic obstructive pulmonary disease and allied cond | 71 | 1.60#(1.25-2.02) | 215 | 1.08(0.94-1.24) | 211 | 0.95(0.83-1.09) | 142 | 0.91(0.76-1.07) | 639 | 1.03(0.95-1.11) |  |  |  |  |
| Stomach and duodenal ulcers | 4 | 2.65(0.72-6.78) | 7 | 1.27(0.51-2.62) | 5 | 1.11(0.36-2.58) | 1 | 0.39(0.01-2.16) | 17 | 1.2(0.7-1.93) |  |  |  |  |
| Chronic liver disease and cirrhosis | 87 | 2.68#(2.15-3.31) | 156 | 1.42#(1.21-1.67) | 94 | 1.25#(1.01-1.53) | 41 | 1.29(0.92-1.74) | 378 | 1.52#(1.37-1.68) |  |  |  |  |
| Nephritis, Nephrotic Syndrome and Nephrosis | 45 | 2.81#(2.05-3.76) | 130 | 1.99#(1.67-2.37) | 99 | 1.55#(1.26-1.88) | 49 | 1.16(0.86-1.54) | 323 | 1.72#(1.54-1.92) |  |  |  |  |
| Complications of pregnancy, childbirth, puerperium | 2 | 30.67#(3.71-110.78) | 4 | 31.16#(8.49-79.79) | 0 | 0(0-422.46) | 0 | 0(0-2613.67) | 6 | 29.45#(10.81-64.11) |  |  |  |  |
| Congenital anomalies | 6 | 2.34(0.86-5.1) | 7 | 0.83(0.34-1.72) | 8 | 1.5(0.65-2.95) | 0 | 0(0-1.74) | 21 | 1.14(0.71-1.74) |  |  |  |  |
| Certain conditions originating in perinatal period | 0 | 0(0-382.64) | 0 | 0(0-115.86) | 0 | 0(0-165.38) | 0 | 0(0-449.19) | 0 | 0(0-51.24) |  |  |  |  |
| Symptoms, Signs and Ill-Defined Conditions | 40 | 4.26#(3.04-5.8) | 113 | 3.43#(2.83-4.13) | 43 | 1.60#(1.16-2.16) | 22 | 1.38(0.86-2.08) | 218 | 2.56#(2.23-2.93) |  |  |  |  |
| Accidents and Adverse Effects | 55 | 1.08(0.81-1.4) | 161 | 0.97(0.82-1.13) | 104 | 0.92(0.75-1.11) | 50 | 0.89(0.66-1.18) | 370 | 0.96(0.86-1.06) |  |  |  |  |
| Suicide and Self-Inflicted Injury | 47 | 2.27#(1.67-3.02) | 79 | 1.19(0.94-1.48) | 56 | 1.32#(1-1.72) | 14 | 0.8(0.44-1.34) | 196 | 1.33#(1.15-1.53) |  |  |  |  |
| Homicide and legal intervention | 3 | 0.64(0.13-1.86) | 15 | 1.09(0.61-1.8) | 5 | 0.7(0.23-1.62) | 1 | 0.41(0.01-2.3) | 24 | 0.86(0.55-1.27) |  |  |  |  |
| Other Cause of Death | 343 | 3.11#(2.79-3.46) | 695 | 1.62#(1.51-1.75) | 510 | 1.25#(1.15-1.37) | 327 | 1.16#(1.04-1.3) | 1875 | 1.53#(1.46-1.6) |  |  |  |  |

# P value less than .05

Supplementary Table 3. Standardized-mortality ratios (SMRs) for each cause of death following CRC diagnosis in patients aged order than 64 years.

| Timing of Deaths After Diagnosis | | | | | | | | | | |  |  |  |  |
| --- | --- | --- | --- | --- | --- | --- | --- | --- | --- | --- | --- | --- | --- | --- |
| Cause of death | <1years | | 1-5years | | 5-10years | | >10years | | Total | |  |  |  |  |
| Selected Events | No. Observed | SMR(95%CI) | No. Observed | SMR(95%CI) | No. Observed | SMR(95%CI) | No. Observed | SMR(95%CI) | No. Observed | SMR(95%CI) |  |  |  |  |
| All cause of death | 45667 | 4.67#(4.63-4.71) | 68607 | 2.11#(2.09-2.13) | 30479 | 1.34#(1.32-1.35) | 11320 | 1.24#(1.22-1.26) | 156073 | 2.10#(2.09-2.11) |  |  |  |  |
| All malignant cancers | 34362 | 16.94#(16.76-17.12) | 44494 | 6.87#(6.8-6.93) | 10521 | 2.56#(2.51-2.61) | 2400 | 1.65#(1.59-1.72) | 91777 | 6.53#(6.48-6.57) |  |  |  |  |
| Colon and Rectum | 31456 | 155.95#(154.23-157.68) | 38139 | 59.88#(59.28-60.49) | 6480 | 16.33#(15.94-16.73) | 891 | 6.37#(5.96-6.81) | 76966 | 55.97#(55.57-56.36) |  |  |  |  |
| In situ, benign, or unknown behavior neoplasms | 124 | 1.98#(1.65-2.36) | 243 | 1.15#(1.01-1.3) | 156 | 1.02(0.87-1.2) | 61 | 0.99(0.76-1.27) | 584 | 1.19#(1.1-1.3) |  |  |  |  |
| Tuberculosis | 3 | 1.21(0.25-3.54) | 8 | 1.05(0.45-2.07) | 4 | 0.87(0.24-2.23) | 4 | 2.5(0.68-6.39) | 19 | 1.17(0.7-1.82) |  |  |  |  |
| syphilis | 0 | 0(0-26.07) | 0 | 0(0-8.37) | 1 | 3.55(0.09-19.76) | 0 | 0(0-35.32) | 1 | 1.03(0.03-5.75) |  |  |  |  |
| Septicemia | 437 | 3.05#(2.78-3.36) | 562 | 1.19#(1.09-1.29) | 362 | 1.11(1-1.23) | 130 | 1.02(0.85-1.21) | 1491 | 1.39#(1.32-1.47) |  |  |  |  |
| Other infectious diseases | 172 | 2.91#(2.49-3.38) | 192 | 0.95(0.82-1.09) | 138 | 0.95(0.79-1.12) | 43 | 0.78(0.57-1.06) | 545 | 1.18#(1.08-1.28) |  |  |  |  |
| Diabetes Mellitus | 400 | 1.42#(1.29-1.57) | 950 | 1.05(0.99-1.12) | 710 | 1.21#(1.12-1.3) | 298 | 1.37#(1.22-1.54) | 2358 | 1.18#(1.14-1.23) |  |  |  |  |
| Alzheimers | 247 | 0.59#(0.52-0.67) | 1020 | 0.67#(0.63-0.71) | 1364 | 1.07#(1.02-1.13) | 807 | 1.33#(1.24-1.43) | 3438 | 0.90#(0.87-0.93) |  |  |  |  |
| Diseasis of hearts | 4493 | 1.58#(1.53-1.62) | 9287 | 1(0.98-1.02) | 7131 | 1.12#(1.09-1.14) | 3041 | 1.19#(1.15-1.24) | 23952 | 1.13#(1.12-1.15) |  |  |  |  |
| Hypertension without heart disease | 174 | 1.50#(1.29-1.74) | 392 | 0.97(0.88-1.07) | 394 | 1.26#(1.14-1.39) | 165 | 1.22#(1.04-1.42) | 1125 | 1.16#(1.1-1.23) |  |  |  |  |
| Cerebrovascular diseases | 909 | 1.33#(1.25-1.42) | 1999 | 0.90#(0.86-0.94) | 1504 | 1(0.95-1.05) | 635 | 1.05(0.97-1.13) | 5047 | 1.01(0.98-1.04) |  |  |  |  |
| Atherosclerosis | 80 | 1.59#(1.26-1.98) | 168 | 1.08(0.92-1.25) | 94 | 0.99(0.8-1.21) | 52 | 1.51#(1.13-1.99) | 394 | 1.17#(1.06-1.3) |  |  |  |  |
| Aortic aneurysm and dissection | 69 | 1.29#(1-1.63) | 139 | 0.84#(0.7-0.99) | 96 | 0.97(0.79-1.19) | 38 | 1.13(0.8-1.55) | 342 | 0.97(0.87-1.08) |  |  |  |  |
| Other diseases of arteries, arterioles, capillaries | 68 | 1.58#(1.23-2) | 126 | 0.89(0.75-1.07) | 113 | 1.18(0.97-1.42) | 42 | 1.12(0.81-1.51) | 349 | 1.1(0.99-1.22) |  |  |  |  |
| Pneumonia and influenza | 398 | 1.41#(1.27-1.55) | 792 | 0.85#(0.79-0.91) | 729 | 1.14#(1.06-1.22) | 291 | 1.15#(1.02-1.29) | 2210 | 1.05#(1-1.09) |  |  |  |  |
| Chronic obstructive pulmonary disease and allied cond | 804 | 1.34#(1.25-1.43) | 2036 | 1.01(0.97-1.06) | 1540 | 1.11#(1.06-1.17) | 575 | 1.08(0.99-1.17) | 4955 | 1.09#(1.06-1.13) |  |  |  |  |
| Stomach and duodenal ulcers | 32 | 2.20#(1.5-3.1) | 45 | 0.99(0.72-1.32) | 36 | 1.27(0.89-1.76) | 10 | 0.95(0.45-1.74) | 123 | 1.24#(1.03-1.48) |  |  |  |  |
| Chronic liver disease and cirrhosis | 125 | 2.39#(1.99-2.84) | 166 | 1.05(0.9-1.22) | 118 | 1.33#(1.1-1.59) | 37 | 1.3(0.92-1.79) | 446 | 1.36#(1.24-1.49) |  |  |  |  |
| Nephritis, Nephrotic Syndrome and Nephrosis | 336 | 1.65#(1.48-1.83) | 682 | 0.99(0.91-1.06) | 582 | 1.17#(1.08-1.27) | 225 | 1.14(0.99-1.3) | 1825 | 1.15#(1.09-1.2) |  |  |  |  |
| Complications of pregnancy, childbirth, puerperium | 0 | 0(0-2589.48) | 0 | 0(0-898.5) | 0 | 0(0-1893.04) | 0 | 0(0-12722.88) | 0 | 0(0-474.84) |  |  |  |  |
| Congenital anomalies | 6 | 1.06(0.39-2.3) | 10 | 0.57(0.27-1.05) | 6 | 0.56(0.2-1.21) | 3 | 0.75(0.15-2.18) | 25 | 0.66#(0.43-0.97) |  |  |  |  |
| Certain conditions originating in perinatal period | 0 | 0(0-203.29) | 0 | 0(0-66.08) | 1 | 31.3(0.79-174.41) | 0 | 0(0-313.28) | 1 | 8.5(0.22-47.34) |  |  |  |  |
| Symptoms, Signs and Ill-Defined Conditions | 170 | 1.42#(1.22-1.65) | 341 | 0.80#(0.71-0.89) | 333 | 0.97(0.87-1.08) | 126 | 0.9(0.75-1.07) | 970 | 0.94(0.88-1) |  |  |  |  |
| Accidents and Adverse Effects | 232 | 1.09(0.96-1.24) | 644 | 0.89#(0.82-0.96) | 520 | 0.96(0.88-1.05) | 257 | 1.11(0.98-1.25) | 1653 | 0.97(0.92-1.01) |  |  |  |  |
| Suicide and Self-Inflicted Injury | 63 | 2.04#(1.56-2.6) | 150 | 1.55#(1.31-1.81) | 62 | 1.05(0.81-1.35) | 25 | 1.21(0.78-1.78) | 300 | 1.45#(1.29-1.62) |  |  |  |  |
| Homicide and legal intervention | 8 | 1.86(0.8-3.67) | 12 | 0.96(0.5-1.68) | 11 | 1.65(0.83-2.96) | 6 | 2.92#(1.07-6.35) | 37 | 1.45#(1.02-2) |  |  |  |  |
| Other Cause of Death | 1955 | 1.33#(1.27-1.39) | 4149 | 0.80#(0.78-0.83) | 3953 | 0.96#(0.93-0.99) | 2049 | 1.14#(1.09-1.19) | 12106 | 0.96#(0.95-0.98) |  |  |  |  |

# P value less than .05

Supplementary Table 4. Standardized-mortality ratios (SMRs) for each cause of death following CRC diagnosis in female patients.

| Timing of Deaths After Diagnosis | | | | | | | | | | |  |  |  |  |
| --- | --- | --- | --- | --- | --- | --- | --- | --- | --- | --- | --- | --- | --- | --- |
| Cause of death | <1years | | 1-5years | | 5-10years | | >10years | | Total | |  |  |  |  |
| Selected Events | No. Observed | SMR(95%CI) | No. Observed | SMR(95%CI) | No. Observed | SMR(95%CI) | No. Observed | SMR(95%CI) | No. Observed | SMR(95%CI) |  |  |  |  |
| All cause of death | 32038 | 5.92#(5.85-5.98) | 52083 | 2.85#(2.83-2.88) | 20065 | 1.51#(1.49-1.53) | 7423 | 1.32#(1.29-1.35) | 111609 | 2.62#(2.6-2.63) |  |  |  |  |
| All malignant cancers | 25686 | 25.18#(24.88-25.49) | 38144 | 11.40#(11.29-11.52) | 8332 | 3.67#(3.59-3.74) | 1816 | 2.01#(1.92-2.1) | 73978 | 9.81#(9.74-9.88) |  |  |  |  |
| Colon and Rectum | 23609 | 218.67#(215.89-221.48) | 34033 | 97.37#(96.34-98.41) | 5881 | 25.29#(24.65-25.94) | 804 | 8.90#(8.3-9.54) | 64327 | 82.43#(81.8-83.07) |  |  |  |  |
| In situ, benign, or unknown behavior neoplasms | 82 | 2.64#(2.1-3.28) | 155 | 1.47#(1.25-1.72) | 93 | 1.21(0.97-1.48) | 41 | 1.26(0.9-1.71) | 371 | 1.51#(1.36-1.67) |  |  |  |  |
| Tuberculosis | 0 | 0(0-3.41) | 4 | 1.2(0.33-3.06) | 2 | 0.96(0.12-3.48) | 1 | 1.31(0.03-7.32) | 7 | 0.96(0.39-1.99) |  |  |  |  |
| syphilis | 0 | 0(0-56.19) | 0 | 0(0-18.2) | 0 | 0(0-27.01) | 0 | 0(0-56.12) | 0 | 0(0-7.84) |  |  |  |  |
| Septicemia | 262 | 3.18#(2.81-3.59) | 352 | 1.28#(1.15-1.42) | 222 | 1.13(0.99-1.29) | 92 | 1.13(0.91-1.38) | 928 | 1.46#(1.37-1.56) |  |  |  |  |
| Other infectious diseases | 117 | 3.18#(2.63-3.81) | 128 | 1(0.83-1.18) | 86 | 0.9(0.72-1.11) | 35 | 0.92(0.64-1.28) | 366 | 1.22#(1.1-1.36) |  |  |  |  |
| Diabetes Mellitus | 219 | 1.40#(1.22-1.6) | 562 | 1.11#(1.02-1.2) | 447 | 1.30#(1.18-1.42) | 194 | 1.41#(1.22-1.63) | 1422 | 1.24#(1.18-1.31) |  |  |  |  |
| Alzheimers | 159 | 0.56#(0.48-0.65) | 655 | 0.63#(0.58-0.68) | 913 | 1.05(0.98-1.12) | 564 | 1.35#(1.24-1.47) | 2291 | 0.88#(0.84-0.92) |  |  |  |  |
| Diseasis of hearts | 2332 | 1.53#(1.47-1.59) | 5008 | 0.99(0.96-1.02) | 3865 | 1.09#(1.05-1.12) | 1768 | 1.21#(1.15-1.27) | 12973 | 1.12#(1.1-1.14) |  |  |  |  |
| Hypertension without heart disease | 111 | 1.49#(1.23-1.8) | 270 | 1.03(0.91-1.16) | 254 | 1.23#(1.08-1.39) | 113 | 1.23#(1.01-1.48) | 748 | 1.18#(1.1-1.27) |  |  |  |  |
| Cerebrovascular diseases | 589 | 1.40#(1.29-1.51) | 1248 | 0.90#(0.85-0.95) | 952 | 0.99(0.92-1.05) | 435 | 1.07(0.97-1.18) | 3224 | 1.02(0.98-1.05) |  |  |  |  |
| Atherosclerosis | 44 | 1.42#(1.03-1.91) | 103 | 1.08(0.88-1.31) | 57 | 0.97(0.73-1.26) | 36 | 1.68#(1.17-2.32) | 240 | 1.16#(1.02-1.32) |  |  |  |  |
| Aortic aneurysm and dissection | 25 | 1.08(0.7-1.59) | 71 | 0.95(0.75-1.2) | 58 | 1.2(0.91-1.55) | 23 | 1.26(0.8-1.89) | 177 | 1.08(0.93-1.25) |  |  |  |  |
| Other diseases of arteries, arterioles, capillaries | 44 | 1.78#(1.29-2.39) | 73 | 0.89(0.7-1.12) | 63 | 1.09(0.84-1.4) | 27 | 1.13(0.74-1.64) | 207 | 1.1(0.96-1.26) |  |  |  |  |
| Pneumonia and influenza | 199 | 1.28#(1.11-1.48) | 462 | 0.91#(0.83-0.99) | 385 | 1.09(0.98-1.2) | 165 | 1.15(0.98-1.34) | 1211 | 1.04(0.98-1.1) |  |  |  |  |
| Chronic obstructive pulmonary disease and allied cond | 407 | 1.32#(1.19-1.45) | 1131 | 1.07#(1.01-1.14) | 894 | 1.15#(1.08-1.23) | 357 | 1.08(0.97-1.2) | 2789 | 1.13#(1.09-1.17) |  |  |  |  |
| Stomach and duodenal ulcers | 21 | 2.59#(1.6-3.96) | 27 | 1.05(0.7-1.53) | 22 | 1.33(0.83-2.01) | 8 | 1.22(0.53-2.4) | 78 | 1.37#(1.08-1.71) |  |  |  |  |
| Chronic liver disease and cirrhosis | 74 | 2.51#(1.97-3.15) | 117 | 1.23#(1.02-1.48) | 81 | 1.31#(1.04-1.63) | 28 | 1.15(0.77-1.67) | 300 | 1.43#(1.27-1.6) |  |  |  |  |
| Nephritis, Nephrotic Syndrome and Nephrosis | 182 | 1.71#(1.47-1.98) | 385 | 1.06(0.96-1.17) | 309 | 1.16#(1.03-1.29) | 145 | 1.31#(1.11-1.55) | 1021 | 1.21#(1.13-1.28) |  |  |  |  |
| Complications of pregnancy, childbirth, puerperium | 7 | 34.78#(13.98-71.67) | 15 | 27.43#(15.35-45.23) | 2 | 8.45#(1.02-30.52) | 2 | 31.38#(3.8-113.34) | 26 | 24.79#(16.2-36.33) |  |  |  |  |
| Congenital anomalies | 6 | 1.43(0.53-3.12) | 9 | 0.68(0.31-1.29) | 6 | 0.71(0.26-1.54) | 0 | 0(0-1.12) | 21 | 0.72(0.45-1.1) |  |  |  |  |
| Certain conditions originating in perinatal period | 0 | 0(0-354.6) | 0 | 0(0-114.73) | 1 | 51.20#(1.3-285.25) | 0 | 0(0-468.7) | 1 | 14.29(0.36-79.64) |  |  |  |  |
| Symptoms, Signs and Ill-Defined Conditions | 125 | 1.59#(1.33-1.9) | 236 | 0.84#(0.73-0.95) | 236 | 1.03(0.91-1.17) | 93 | 0.99(0.8-1.21) | 690 | 1.01(0.94-1.09) |  |  |  |  |
| Accidents and Adverse Effects | 123 | 1.05(0.87-1.25) | 361 | 0.89#(0.8-0.99) | 293 | 0.95(0.85-1.07) | 149 | 1.1(0.93-1.29) | 926 | 0.96(0.9-1.02) |  |  |  |  |
| Suicide and Self-Inflicted Injury | 16 | 1.72(0.98-2.79) | 31 | 1.06(0.72-1.51) | 22 | 1.25(0.78-1.89) | 10 | 1.56(0.75-2.87) | 79 | 1.26(1-1.57) |  |  |  |  |
| Homicide and legal intervention | 3 | 0.94(0.19-2.74) | 7 | 0.73(0.29-1.5) | 14 | 2.50#(1.37-4.2) | 3 | 1.5(0.31-4.4) | 27 | 1.32(0.87-1.92) |  |  |  |  |
| Other Cause of Death | 1205 | 1.37#(1.29-1.45) | 2529 | 0.81#(0.78-0.84) | 2456 | 0.97(0.93-1.01) | 1318 | 1.15#(1.09-1.22) | 7508 | 0.98#(0.96-1) |  |  |  |  |

# P value less than .05

Supplementary Table 5. Standardized-mortality ratios (SMRs) for each cause of death following CRC diagnosis in male patients.

| Timing of Deaths After Diagnosis | | | | | | | | | | |  |  |  |  |
| --- | --- | --- | --- | --- | --- | --- | --- | --- | --- | --- | --- | --- | --- | --- |
| Cause of death | <1years | | 1-5years | | 5-10years | | >10years | | Total | |  |  |  |  |
| Selected Events | No. Observed | SMR(95%CI) | No. Observed | SMR(95%CI) | No. Observed | SMR(95%CI) | No. Observed | SMR(95%CI) | No. Observed | SMR(95%CI) |  |  |  |  |
| All cause of death | 31316 | 5.66#(5.6-5.73) | 58268 | 3.12#(3.09-3.14) | 22201 | 1.66#(1.63-1.68) | 7447 | 1.26#(1.24-1.29) | 119232 | 2.74#(2.72-2.75) |  |  |  |  |
| All malignant cancers | 24425 | 17.67#(17.45-17.89) | 43748 | 9.49#(9.4-9.58) | 10752 | 3.41#(3.34-3.47) | 2274 | 1.74#(1.67-1.82) | 81199 | 7.77#(7.72-7.82) |  |  |  |  |
| Colon and Rectum | 22285 | 175.03#(172.74-177.34) | 38626 | 92.73#(91.81-93.66) | 7431 | 27.06#(26.45-27.68) | 933 | 8.50#(7.97-9.07) | 69275 | 74.63#(74.08-75.19) |  |  |  |  |
| In situ, benign, or unknown behavior neoplasms | 74 | 2.02#(1.58-2.53) | 172 | 1.35#(1.15-1.56) | 113 | 1.17(0.96-1.4) | 43 | 0.98(0.71-1.32) | 402 | 1.32#(1.19-1.45) |  |  |  |  |
| Tuberculosis | 5 | 2.68(0.87-6.26) | 6 | 1.02(0.37-2.21) | 4 | 1.07(0.29-2.74) | 5 | 3.46#(1.12-8.07) | 20 | 1.54(0.94-2.39) |  |  |  |  |
| syphilis | 0 | 0(0-36.03) | 0 | 0(0-10.91) | 1 | 4.25(0.11-23.67) | 0 | 0(0-40.38) | 1 | 1.3(0.03-7.26) |  |  |  |  |
| Septicemia | 280 | 3.62#(3.21-4.07) | 400 | 1.52#(1.37-1.67) | 234 | 1.22#(1.07-1.39) | 88 | 1.03(0.83-1.27) | 1002 | 1.62#(1.52-1.73) |  |  |  |  |
| Other infectious diseases | 150 | 3.05#(2.58-3.58) | 192 | 1.18#(1.02-1.36) | 114 | 1.04(0.86-1.25) | 42 | 0.97(0.7-1.31) | 498 | 1.37#(1.25-1.49) |  |  |  |  |
| Diabetes Mellitus | 272 | 1.60#(1.42-1.8) | 653 | 1.15#(1.06-1.24) | 500 | 1.26#(1.15-1.37) | 243 | 1.43#(1.26-1.62) | 1668 | 1.28#(1.22-1.34) |  |  |  |  |
| Alzheimers | 89 | 0.66#(0.53-0.82) | 374 | 0.75#(0.68-0.83) | 483 | 1.13#(1.03-1.24) | 293 | 1.31#(1.17-1.47) | 1239 | 0.97(0.91-1.02) |  |  |  |  |
| Diseasis of hearts | 2742 | 1.72#(1.66-1.79) | 5571 | 1.05#(1.02-1.08) | 4317 | 1.16#(1.13-1.2) | 1867 | 1.15#(1.1-1.21) | 14497 | 1.19#(1.17-1.21) |  |  |  |  |
| Hypertension without heart disease | 85 | 1.66#(1.32-2.05) | 185 | 1.02(0.88-1.18) | 177 | 1.25#(1.07-1.44) | 91 | 1.35#(1.09-1.66) | 538 | 1.22#(1.12-1.33) |  |  |  |  |
| Cerebrovascular diseases | 415 | 1.37#(1.25-1.51) | 962 | 0.96(0.9-1.03) | 725 | 1.04(0.97-1.12) | 304 | 0.99(0.89-1.11) | 2406 | 1.05#(1-1.09) |  |  |  |  |
| Atherosclerosis | 41 | 1.96#(1.4-2.65) | 73 | 1.1(0.86-1.38) | 39 | 0.93(0.66-1.27) | 20 | 1.2(0.73-1.85) | 173 | 1.19#(1.02-1.38) |  |  |  |  |
| Aortic aneurysm and dissection | 54 | 1.51#(1.13-1.97) | 86 | 0.76#(0.61-0.94) | 54 | 0.78(0.59-1.02) | 25 | 0.97(0.62-1.42) | 219 | 0.9(0.78-1.03) |  |  |  |  |
| Other diseases of arteries, arterioles, capillaries | 34 | 1.58#(1.09-2.2) | 69 | 0.95(0.74-1.21) | 66 | 1.29#(1-1.65) | 29 | 1.31(0.87-1.88) | 198 | 1.19#(1.03-1.36) |  |  |  |  |
| Pneumonia and influenza | 229 | 1.60#(1.4-1.82) | 426 | 0.88#(0.8-0.97) | 417 | 1.21#(1.1-1.34) | 169 | 1.13(0.97-1.31) | 1241 | 1.11#(1.05-1.17) |  |  |  |  |
| Chronic obstructive pulmonary disease and allied cond | 475 | 1.41#(1.28-1.54) | 1136 | 0.98(0.92-1.04) | 876 | 1.03(0.97-1.11) | 369 | 0.99(0.89-1.1) | 2856 | 1.05#(1.01-1.09) |  |  |  |  |
| Stomach and duodenal ulcers | 16 | 1.97#(1.12-3.19) | 25 | 0.96(0.62-1.42) | 21 | 1.25(0.77-1.9) | 3 | 0.43(0.09-1.26) | 65 | 1.12(0.87-1.43) |  |  |  |  |
| Chronic liver disease and cirrhosis | 153 | 2.54#(2.15-2.97) | 234 | 1.21#(1.06-1.38) | 162 | 1.34#(1.14-1.56) | 62 | 1.33#(1.02-1.71) | 611 | 1.45#(1.34-1.57) |  |  |  |  |
| Nephritis, Nephrotic Syndrome and Nephrosis | 204 | 1.77#(1.54-2.03) | 445 | 1.11#(1.01-1.22) | 392 | 1.31#(1.18-1.44) | 137 | 1.03(0.86-1.22) | 1178 | 1.24#(1.17-1.31) |  |  |  |  |
| Complications of pregnancy, childbirth, puerperium | 0 | 000 | 0 | 0(0-0) | 0 | 0(-0) | 0 | 0(0-0) | 0 | 0(0-0) |  |  |  |  |
| Congenital anomalies | 7 | 1.51(0.61-3.12) | 10 | 0.68(0.33-1.25) | 9 | 0.97(0.44-1.85) | 3 | 0.81(0.17-2.38) | 29 | 0.9(0.6-1.29) |  |  |  |  |
| Certain conditions originating in perinatal period | 0 | 0(0-159.58) | 1 | 13.63(0.35-75.93) | 0 | 0(0-83.33) | 0 | 0(0-231.42) | 1 | 6.38(0.16-35.56) |  |  |  |  |
| Symptoms, Signs and Ill-Defined Conditions | 98 | 1.86#(1.51-2.27) | 252 | 1.36#(1.2-1.54) | 151 | 1.04(0.88-1.22) | 59 | 0.91(0.7-1.18) | 560 | 1.25#(1.15-1.36) |  |  |  |  |
| Accidents and Adverse Effects | 187 | 1.14(0.98-1.31) | 508 | 0.93(0.85-1.01) | 373 | 0.96(0.86-1.06) | 179 | 1.05(0.9-1.21) | 1247 | 0.98(0.93-1.04) |  |  |  |  |
| Suicide and Self-Inflicted Injury | 100 | 2.01#(1.63-2.44) | 235 | 1.47#(1.29-1.67) | 120 | 1.19(0.98-1.42) | 39 | 0.99(0.7-1.35) | 494 | 1.41#(1.29-1.54) |  |  |  |  |
| Homicide and legal intervention | 9 | 1.01(0.46-1.91) | 32 | 1.26(0.86-1.78) | 8 | 0.62(0.27-1.22) | 5 | 1.2(0.39-2.81) | 54 | 1.05(0.79-1.37) |  |  |  |  |
| Other Cause of Death | 1172 | 1.64#(1.55-1.74) | 2473 | 0.97(0.94-1.01) | 2093 | 1.03(0.99-1.08) | 1098 | 1.13#(1.06-1.2) | 6836 | 1.09#(1.07-1.12) |  |  |  |  |

# P value less than .05

Supplementary Table 6. Standardized-mortality ratios (SMRs) for each cause of death following CRC diagnosis in white patients.

| Timing of Deaths After Diagnosis | | | | | | | | | | |  |  |  |  |
| --- | --- | --- | --- | --- | --- | --- | --- | --- | --- | --- | --- | --- | --- | --- |
| Cause of death | <1years | | 1-5years | | 5-10years | | >10years | | Total | |  |  |  |  |
| Selected Events | No. Observed | SMR(95%CI) | No. Observed | SMR(95%CI) | No. Observed | SMR(95%CI) | No. Observed | SMR(95%CI) | No. Observed | SMR(95%CI) |  |  |  |  |
| All cause of death | 50520 | 5.44#(5.4-5.49) | 87524 | 2.77#(2.75-2.78) | 35340 | 1.53#(1.52-1.55) | 12598 | 1.26#(1.24-1.29) | 185982 | 2.51#(2.5-2.53) |  |  |  |  |
| All malignant cancers | 39719 | 19.78#(19.59-19.98) | 63777 | 9.52#(9.44-9.59) | 15402 | 3.35#(3.29-3.4) | 3366 | 1.79#(1.73-1.85) | 122264 | 8.05#(8-8.09) |  |  |  |  |
| Colon and Rectum | 36415 | 188.51#(186.58-190.46) | 56434 | 89.05#(88.32-89.79) | 10595 | 25.15#(24.67-25.63) | 1393 | 8.35#(7.92-8.8) | 104837 | 74.09#(73.64-74.54) |  |  |  |  |
| In situ, benign, or unknown behavior neoplasms | 130 | 2.19#(1.83-2.6) | 271 | 1.32#(1.16-1.48) | 173 | 1.12(0.96-1.3) | 74 | 1.09(0.85-1.37) | 648 | 1.33#(1.23-1.44) |  |  |  |  |
| Tuberculosis | 2 | 1.35(0.16-4.89) | 1 | 0.21(0.01-1.19) | 3 | 1.02(0.21-2.99) | 2 | 1.77(0.21-6.39) | 8 | 0.78(0.34-1.54) |  |  |  |  |
| syphilis | 0 | 0(0-46.47) | 0 | 0(0-13.84) | 1 | 5.16(0.13-28.74) | 0 | 0(0-42.81) | 1 | 1.6(0.04-8.9) |  |  |  |  |
| Septicemia | 420 | 3.33#(3.01-3.66) | 563 | 1.30#(1.2-1.41) | 372 | 1.18#(1.06-1.3) | 145 | 1.06(0.89-1.24) | 1500 | 1.48#(1.41-1.56) |  |  |  |  |
| Other infectious diseases | 210 | 3.25#(2.83-3.72) | 246 | 1.09(0.96-1.23) | 166 | 1(0.85-1.16) | 66 | 0.98(0.76-1.24) | 688 | 1.31#(1.22-1.41) |  |  |  |  |
| Diabetes Mellitus | 368 | 1.48#(1.33-1.64) | 918 | 1.11#(1.03-1.18) | 767 | 1.33#(1.24-1.43) | 339 | 1.42#(1.27-1.57) | 2392 | 1.26#(1.21-1.31) |  |  |  |  |
| Alzheimers | 208 | 0.55#(0.48-0.63) | 918 | 0.66#(0.62-0.7) | 1255 | 1.07#(1.01-1.13) | 771 | 1.35#(1.25-1.44) | 3152 | 0.90#(0.87-0.93) |  |  |  |  |
| Diseasis of hearts | 4159 | 1.57#(1.52-1.61) | 8893 | 1(0.98-1.02) | 7039 | 1.12#(1.09-1.14) | 3109 | 1.16#(1.12-1.2) | 23200 | 1.13#(1.12-1.14) |  |  |  |  |
| Hypertension without heart disease | 147 | 1.54#(1.3-1.81) | 347 | 1.01(0.91-1.13) | 336 | 1.22#(1.1-1.36) | 164 | 1.30#(1.11-1.51) | 994 | 1.19#(1.11-1.26) |  |  |  |  |
| Cerebrovascular diseases | 786 | 1.31#(1.22-1.4) | 1807 | 0.90#(0.86-0.95) | 1423 | 1.02(0.96-1.07) | 616 | 1.02(0.94-1.11) | 4632 | 1.01(0.98-1.04) |  |  |  |  |
| Atherosclerosis | 76 | 1.64#(1.29-2.05) | 158 | 1.08(0.92-1.27) | 81 | 0.9(0.71-1.11) | 50 | 1.47#(1.09-1.93) | 365 | 1.15#(1.04-1.28) |  |  |  |  |
| Aortic aneurysm and dissection | 65 | 1.27(0.98-1.61) | 138 | 0.84#(0.71-0.99) | 92 | 0.9(0.72-1.1) | 45 | 1.16(0.85-1.56) | 340 | 0.95(0.85-1.06) |  |  |  |  |
| Other diseases of arteries, arterioles, capillaries | 61 | 1.58#(1.21-2.03) | 122 | 0.94(0.78-1.12) | 111 | 1.2(0.99-1.45) | 43 | 1.1(0.79-1.48) | 337 | 1.12#(1.01-1.25) |  |  |  |  |
| Pneumonia and influenza | 353 | 1.40#(1.25-1.55) | 750 | 0.89#(0.82-0.95) | 682 | 1.15#(1.06-1.24) | 279 | 1.12(1-1.26) | 2064 | 1.06#(1.02-1.11) |  |  |  |  |
| Chronic obstructive pulmonary disease and allied cond | 775 | 1.32#(1.23-1.42) | 2016 | 1(0.96-1.05) | 1623 | 1.10#(1.05-1.15) | 650 | 1.02(0.94-1.1) | 5064 | 1.07#(1.04-1.1) |  |  |  |  |
| Stomach and duodenal ulcers | 30 | 2.19#(1.48-3.13) | 44 | 1.01(0.73-1.35) | 35 | 1.23(0.86-1.72) | 10 | 0.87(0.42-1.6) | 119 | 1.22#(1.01-1.46) |  |  |  |  |
| Chronic liver disease and cirrhosis | 208 | 2.72#(2.37-3.12) | 303 | 1.23#(1.09-1.37) | 218 | 1.38#(1.2-1.57) | 77 | 1.24(0.98-1.55) | 806 | 1.48#(1.38-1.59) |  |  |  |  |
| Nephritis, Nephrotic Syndrome and Nephrosis | 281 | 1.62#(1.44-1.82) | 626 | 1.03(0.95-1.12) | 552 | 1.21#(1.11-1.31) | 228 | 1.15#(1.01-1.31) | 1687 | 1.18#(1.12-1.23) |  |  |  |  |
| Complications of pregnancy, childbirth, puerperium | 5 | 41.56#(13.5-97) | 10 | 29.52#(14.16-54.29) | 1 | 6.49(0.16-36.14) | 0 | 0(0-87.82) | 16 | 24.42#(13.96-39.65) |  |  |  |  |
| Congenital anomalies | 12 | 1.58(0.82-2.76) | 16 | 0.66(0.38-1.07) | 13 | 0.84(0.45-1.44) | 3 | 0.49(0.1-1.43) | 44 | 0.82(0.6-1.11) |  |  |  |  |
| Certain conditions originating in perinatal period | 0 | 0(0-140.77) | 1 | 12.04(0.3-67.11) | 1 | 19.57(0.5-109.06) | 0 | 0(0-184.91) | 2 | 11.09#(1.34-40.08) |  |  |  |  |
| Symptoms, Signs and Ill-Defined Conditions | 174 | 1.53#(1.31-1.78) | 403 | 0.98(0.89-1.08) | 331 | 1(0.89-1.11) | 129 | 0.91(0.76-1.09) | 1037 | 1.04(0.98-1.11) |  |  |  |  |
| Accidents and Adverse Effects | 267 | 1.1(0.97-1.24) | 754 | 0.91#(0.84-0.97) | 589 | 0.96(0.88-1.04) | 289 | 1.05(0.94-1.18) | 1899 | 0.97(0.92-1.01) |  |  |  |  |
| Suicide and Self-Inflicted Injury | 109 | 2.01#(1.65-2.43) | 235 | 1.35#(1.19-1.54) | 132 | 1.20#(1-1.42) | 45 | 1.05(0.76-1.4) | 521 | 1.37#(1.25-1.49) |  |  |  |  |
| Homicide and legal intervention | 8 | 1.11(0.48-2.18) | 19 | 0.87(0.53-1.37) | 14 | 1.15(0.63-1.93) | 6 | 1.41(0.52-3.08) | 47 | 1.04(0.76-1.38) |  |  |  |  |
| Other Cause of Death | 1947 | 1.41#(1.35-1.48) | 4188 | 0.85#(0.82-0.87) | 3928 | 0.98(0.95-1.01) | 2092 | 1.12#(1.07-1.17) | 12155 | 1(0.98-1.01) |  |  |  |  |

# P value less than .05

Supplementary Table 7. Standardized-mortality ratios (SMRs) for each cause of death following CRC diagnosis in black patients.

| Timing of Deaths After Diagnosis | | | | | | | | | | |  |  |  |  |
| --- | --- | --- | --- | --- | --- | --- | --- | --- | --- | --- | --- | --- | --- | --- |
| Cause of death | <1years | | 1-5years | | 5-10years | | >10years | | Total | |  |  |  |  |
| Selected Events | No. Observed | SMR(95%CI) | No. Observed | SMR(95%CI) | No. Observed | SMR(95%CI) | No. Observed | SMR(95%CI) | No. Observed | SMR(95%CI) |  |  |  |  |
| All cause of death | 8622 | 7.48#(7.32-7.63) | 14597 | 4.07#(4-4.13) | 4270 | 1.77#(1.72-1.83) | 1406 | 1.41#(1.34-1.49) | 28895 | 3.55#(3.51-3.59) |  |  |  |  |
| All malignant cancers | 6909 | 25.09#(24.5-25.69) | 11488 | 13.44#(13.2-13.69) | 2206 | 4.01#(3.84-4.18) | 420 | 1.95#(1.77-2.15) | 21023 | 11.09#(10.94-11.24) |  |  |  |  |
| Colon and Rectum | 6258 | 209.99#(204.82-215.26) | 10247 | 112.01#(109.86-114.2) | 1626 | 28.17#(26.81-29.57) | 203 | 9.20#(7.98-10.55) | 18334 | 91.18#(89.86-92.51) |  |  |  |  |
| In situ, benign, or unknown behavior neoplasms | 14 | 2.72#(1.48-4.56) | 35 | 2.15#(1.5-2.99) | 17 | 1.5(0.87-2.39) | 7 | 1.41(0.57-2.91) | 73 | 1.93#(1.52-2.43) |  |  |  |  |
| Tuberculosis | 1 | 1.82(0.05-10.17) | 3 | 1.95(0.4-5.7) | 1 | 1.25(0.03-6.96) | 0 | 0(0-13.78) | 5 | 1.59(0.51-3.7) |  |  |  |  |
| syphilis | 0 | 0(0-45.74) | 0 | 0(0-14.76) | 0 | 0(0-22.65) | 0 | 0(0-57.36) | 0 | 0(0-6.61) |  |  |  |  |
| Septicemia | 99 | 3.70#(3.01-4.5) | 152 | 1.82#(1.54-2.14) | 63 | 1.14(0.87-1.45) | 28 | 1.25(0.83-1.81) | 342 | 1.82#(1.63-2.02) |  |  |  |  |
| Other infectious diseases | 45 | 2.73#(1.99-3.66) | 52 | 1.08(0.81-1.41) | 25 | 0.91(0.59-1.35) | 7 | 0.74(0.3-1.52) | 129 | 1.27#(1.06-1.51) |  |  |  |  |
| Diabetes Mellitus | 91 | 1.65#(1.33-2.03) | 194 | 1.14(0.98-1.31) | 121 | 1.09(0.9-1.3) | 61 | 1.37#(1.05-1.76) | 467 | 1.22#(1.12-1.34) |  |  |  |  |
| Alzheimers | 33 | 1.22(0.84-1.72) | 65 | 0.71#(0.55-0.9) | 89 | 1.14(0.91-1.4) | 55 | 1.34#(1.01-1.74) | 242 | 1.02(0.89-1.15) |  |  |  |  |
| Diseasis of hearts | 666 | 2.04#(1.89-2.2) | 1144 | 1.15#(1.08-1.21) | 760 | 1.17#(1.09-1.26) | 346 | 1.31#(1.18-1.46) | 2916 | 1.30#(1.26-1.35) |  |  |  |  |
| Hypertension without heart disease | 40 | 1.82#(1.3-2.48) | 73 | 1.04(0.82-1.31) | 58 | 1.17(0.89-1.51) | 30 | 1.4(0.94-2) | 201 | 1.23#(1.07-1.41) |  |  |  |  |
| Cerebrovascular diseases | 136 | 1.71#(1.43-2.02) | 241 | 0.99(0.87-1.13) | 144 | 0.9(0.76-1.06) | 69 | 1.03(0.8-1.3) | 590 | 1.07(0.99-1.16) |  |  |  |  |
| Atherosclerosis | 5 | 1.31(0.43-3.06) | 12 | 1.08(0.56-1.89) | 7 | 1.04(0.42-2.14) | 5 | 1.9(0.62-4.43) | 29 | 1.19(0.8-1.71) |  |  |  |  |
| Aortic aneurysm and dissection | 7 | 1.55(0.62-3.2) | 10 | 0.75(0.36-1.38) | 7 | 0.89(0.36-1.82) | 1 | 0.34(0.01-1.89) | 25 | 0.87(0.56-1.29) |  |  |  |  |
| Other diseases of arteries, arterioles, capillaries | 13 | 2.13#(1.14-3.65) | 18 | 0.95(0.57-1.51) | 16 | 1.26(0.72-2.04) | 6 | 1.12(0.41-2.43) | 53 | 1.23(0.92-1.61) |  |  |  |  |
| Pneumonia and influenza | 43 | 1.70#(1.23-2.29) | 70 | 0.9(0.7-1.14) | 58 | 1.11(0.84-1.44) | 21 | 0.97(0.6-1.49) | 192 | 1.09(0.94-1.25) |  |  |  |  |
| Chronic obstructive pulmonary disease and allied cond | 63 | 1.59#(1.22-2.04) | 153 | 1.21#(1.02-1.42) | 84 | 0.94(0.75-1.16) | 43 | 1.12(0.81-1.51) | 343 | 1.17#(1.05-1.3) |  |  |  |  |
| Stomach and duodenal ulcers | 2 | 1.45(0.18-5.25) | 6 | 1.48(0.54-3.21) | 2 | 0.83(0.1-2.98) | 0 | 0(0-4.07) | 10 | 1.14(0.55-2.1) |  |  |  |  |
| Chronic liver disease and cirrhosis | 9 | 1.08(0.5-2.06) | 24 | 0.97(0.62-1.45) | 14 | 0.99(0.54-1.66) | 8 | 1.6(0.69-3.16) | 55 | 1.06(0.79-1.37) |  |  |  |  |
| Nephritis, Nephrotic Syndrome and Nephrosis | 81 | 2.20#(1.75-2.74) | 155 | 1.33#(1.12-1.55) | 112 | 1.39#(1.15-1.67) | 43 | 1.29(0.94-1.74) | 391 | 1.46#(1.32-1.61) |  |  |  |  |
| Complications of pregnancy, childbirth, puerperium | 1 | 14.61(0.37-81.41) | 5 | 28.21#(9.16-65.82) | 1 | 13.91(0.35-77.52) | 2 | 103.30#(12.51-373.17) | 9 | 26.71#(12.21-50.71) |  |  |  |  |
| Congenital anomalies | 1 | 1.18(0.03-6.59) | 1 | 0.4(0.01-2.23) | 1 | 0.68(0.02-3.82) | 0 | 0(0-6.93) | 3 | 0.56(0.12-1.64) |  |  |  |  |
| Certain conditions originating in perinatal period | 0 | 0(0-655.22) | 0 | 0(0-204.08) | 0 | 0(0-324.32) | 0 | 0(0-958.7) | 0 | 0(0-94.76) |  |  |  |  |
| Symptoms, Signs and Ill-Defined Conditions | 37 | 2.82#(1.99-3.89) | 60 | 1.43#(1.09-1.84) | 38 | 1.25(0.89-1.72) | 18 | 1.47(0.87-2.32) | 153 | 1.56#(1.33-1.83) |  |  |  |  |
| Accidents and Adverse Effects | 28 | 1.13(0.75-1.64) | 74 | 1(0.78-1.25) | 35 | 0.77(0.54-1.07) | 22 | 1.23(0.77-1.86) | 159 | 0.98(0.83-1.14) |  |  |  |  |
| Suicide and Self-Inflicted Injury | 4 | 1.74(0.48-4.47) | 10 | 1.53(0.73-2.81) | 4 | 1.14(0.31-2.92) | 1 | 0.84(0.02-4.67) | 19 | 1.4(0.85-2.19) |  |  |  |  |
| Homicide and legal intervention | 4 | 0.97(0.27-2.5) | 15 | 1.37(0.77-2.26) | 7 | 1.38(0.56-2.85) | 1 | 0.66(0.02-3.7) | 27 | 1.25(0.82-1.82) |  |  |  |  |
| Other Cause of Death | 290 | 1.96#(1.74-2.2) | 537 | 1.12#(1.03-1.22) | 400 | 1.11#(1-1.22) | 212 | 1.30#(1.13-1.48) | 1439 | 1.25#(1.18-1.31) |  |  |  |  |

# P value less than .05

Supplementary Table 8. Standardized-mortality ratios (SMRs) for each cause of death following CRC diagnosis in patients of other races.

| Timing of Deaths After Diagnosis | | | | | | | | | | |  |  |  |  |
| --- | --- | --- | --- | --- | --- | --- | --- | --- | --- | --- | --- | --- | --- | --- |
| Cause of death | <1years | | 1-5years | | 5-10years | | >10years | | Total | |  |  |  |  |
| Selected Events | No. Observed | SMR(95%CI) | No. Observed | SMR(95%CI) | No. Observed | SMR(95%CI) | No. Observed | SMR(95%CI) | No. Observed | SMR(95%CI) |  |  |  |  |
| All cause of death | 4212 | 8.24#(8-8.5) | 8230 | 4.76#(4.66-4.87) | 2656 | 2.09#(2.01-2.17) | 866 | 1.58#(1.47-1.69) | 15964 | 3.93#(3.87-4) |  |  |  |  |
| All malignant cancers | 3483 | 29.24#(28.27-30.22) | 6627 | 16.65#(16.25-17.05) | 1476 | 5.34#(5.07-5.62) | 304 | 2.74#(2.44-3.07) | 11890 | 13.14#(12.91-13.38) |  |  |  |  |
| Colon and Rectum | 3221 | 261.60#(252.64-270.79) | 5978 | 146.24#(142.56-150) | 1091 | 38.82#(36.55-41.19) | 141 | 12.68#(10.67-14.95) | 10431 | 112.87#(110.71-115.05) |  |  |  |  |
| In situ, benign, or unknown behavior neoplasms | 12 | 3.81#(1.97-6.65) | 21 | 1.92#(1.19-2.94) | 16 | 1.93#(1.1-3.13) | 3 | 0.83(0.17-2.41) | 52 | 2.00#(1.49-2.62) |  |  |  |  |
| Tuberculosis | 2 | 2.18(0.26-7.87) | 6 | 1.98(0.73-4.3) | 2 | 0.96(0.12-3.48) | 4 | 4.95#(1.35-12.66) | 14 | 2.05#(1.12-3.44) |  |  |  |  |
| syphilis | 0 | 0(0-460.55) | 0 | 0(0-152.84) | 0 | 0(0-242.07) | 0 | 0(0-557.84) | 0 | 0(0-68.32) |  |  |  |  |
| Septicemia | 23 | 3.45#(2.19-5.18) | 37 | 1.63#(1.14-2.24) | 21 | 1.26(0.78-1.92) | 7 | 0.99(0.4-2.05) | 88 | 1.66#(1.33-2.04) |  |  |  |  |
| Other infectious diseases | 12 | 2.42#(1.25-4.23) | 22 | 1.34(0.84-2.02) | 9 | 0.8(0.36-1.51) | 4 | 0.9(0.24-2.3) | 47 | 1.26(0.93-1.68) |  |  |  |  |
| Diabetes Mellitus | 32 | 1.42(0.97-2) | 103 | 1.36#(1.11-1.64) | 59 | 1.08(0.82-1.39) | 37 | 1.62#(1.14-2.23) | 231 | 1.31#(1.15-1.49) |  |  |  |  |
| Alzheimers | 7 | 0.52(0.21-1.07) | 46 | 0.91(0.67-1.22) | 52 | 1.12(0.84-1.47) | 31 | 1.22(0.83-1.73) | 136 | 1(0.84-1.19) |  |  |  |  |
| Diseasis of hearts | 249 | 1.85#(1.63-2.1) | 542 | 1.21#(1.11-1.32) | 383 | 1.19#(1.07-1.32) | 180 | 1.32#(1.13-1.52) | 1354 | 1.30#(1.23-1.37) |  |  |  |  |
| Hypertension without heart disease | 9 | 1.08(0.5-2.06) | 35 | 1.18(0.83-1.65) | 37 | 1.52#(1.07-2.1) | 10 | 0.86(0.41-1.58) | 91 | 1.23(0.99-1.51) |  |  |  |  |
| Cerebrovascular diseases | 82 | 1.93#(1.54-2.4) | 162 | 1.16(0.98-1.35) | 110 | 1.1(0.91-1.33) | 54 | 1.26(0.94-1.64) | 408 | 1.25#(1.14-1.38) |  |  |  |  |
| Atherosclerosis | 4 | 2.48(0.68-6.35) | 6 | 1.16(0.43-2.53) | 8 | 2.3(0.99-4.53) | 1 | 0.69(0.02-3.82) | 19 | 1.62(0.98-2.53) |  |  |  |  |
| Aortic aneurysm and dissection | 7 | 2.21(0.89-4.55) | 9 | 0.87(0.4-1.66) | 13 | 1.94#(1.03-3.31) | 2 | 0.79(0.1-2.85) | 31 | 1.37(0.93-1.94) |  |  |  |  |
| Other diseases of arteries, arterioles, capillaries | 4 | 2.55(0.69-6.53) | 2 | 0.38(0.05-1.38) | 2 | 0.54(0.06-1.94) | 7 | 4.38#(1.76-9.03) | 15 | 1.24(0.69-2.04) |  |  |  |  |
| Pneumonia and influenza | 32 | 1.64#(1.12-2.32) | 68 | 1.02(0.79-1.29) | 62 | 1.21(0.93-1.55) | 34 | 1.48#(1.02-2.06) | 196 | 1.22#(1.06-1.4) |  |  |  |  |
| Chronic obstructive pulmonary disease and allied cond | 44 | 2.07#(1.5-2.77) | 98 | 1.35#(1.09-1.64) | 63 | 1.16(0.89-1.48) | 33 | 1.4(0.96-1.97) | 238 | 1.38#(1.21-1.57) |  |  |  |  |
| Stomach and duodenal ulcers | 5 | 4.21#(1.37-9.81) | 2 | 0.52(0.06-1.87) | 6 | 2.25(0.83-4.9) | 1 | 0.93(0.02-5.2) | 14 | 1.59(0.87-2.67) |  |  |  |  |
| Chronic liver disease and cirrhosis | 10 | 1.94(0.93-3.57) | 24 | 1.46(0.93-2.17) | 11 | 1.07(0.53-1.91) | 5 | 1.32(0.43-3.08) | 50 | 1.40#(1.04-1.84) |  |  |  |  |
| Nephritis, Nephrotic Syndrome and Nephrosis | 24 | 2.14#(1.37-3.18) | 49 | 1.26(0.93-1.67) | 37 | 1.26(0.88-1.73) | 11 | 0.86(0.43-1.54) | 121 | 1.31#(1.09-1.56) |  |  |  |  |
| Complications of pregnancy, childbirth, puerperium | 1 | 79.96#(2.02-445.5) | 0 | 0(0-119.29) | 0 | 0(0-346.86) | 0 | 0(0-1551.79) | 1 | 17.72(0.45-98.72) |  |  |  |  |
| Congenital anomalies | 0 | 0(0-9.86) | 2 | 1.69(0.2-6.11) | 1 | 1.31(0.03-7.27) | 0 | 0(0-12.04) | 3 | 1.14(0.24-3.33) |  |  |  |  |
| Certain conditions originating in perinatal period | 0 | 0(0-2189.12) | 0 | 0(0-833.34) | 0 | 0(0-2755.02) | 0 | 0(0-272945.32) | 0 | 0(0-494.21) |  |  |  |  |
| Symptoms, Signs and Ill-Defined Conditions | 12 | 2.78#(1.44-4.86) | 25 | 1.66#(1.07-2.45) | 18 | 1.49(0.89-2.36) | 5 | 0.94(0.31-2.2) | 60 | 1.63#(1.25-2.1) |  |  |  |  |
| Accidents and Adverse Effects | 15 | 1.03(0.57-1.69) | 41 | 0.84(0.61-1.14) | 42 | 1.21(0.87-1.64) | 17 | 1.14(0.66-1.82) | 115 | 1.02(0.84-1.22) |  |  |  |  |
| Suicide and Self-Inflicted Injury | 3 | 1.1(0.23-3.21) | 21 | 2.47#(1.53-3.78) | 6 | 1.19(0.44-2.59) | 3 | 1.67(0.34-4.88) | 33 | 1.83#(1.26-2.57) |  |  |  |  |
| Homicide and legal intervention | 0 | 0(0-4.62) | 5 | 2.17(0.7-5.05) | 1 | 0.82(0.02-4.58) | 1 | 2.51(0.06-13.97) | 7 | 1.48(0.6-3.05) |  |  |  |  |
| Other Cause of Death | 140 | 2.08#(1.75-2.45) | 277 | 1.16#(1.03-1.31) | 221 | 1.15#(1-1.31) | 112 | 1.25#(1.03-1.5) | 750 | 1.28#(1.19-1.37) |  |  |  |  |

# P value less than .05

Supplementary Table 9. Standardized-mortality ratios (SMRs) for each cause of death following CRC diagnosis in distant stage patients.

| Timing of Deaths After Diagnosis | | | | | | | | | | |  |  |  |  |
| --- | --- | --- | --- | --- | --- | --- | --- | --- | --- | --- | --- | --- | --- | --- |
| Cause of death | <1years | | 1-5years | | 5-10years | | >10years | | Total | |  |  |  |  |
| Selected Events | No. Observed | SMR(95%CI) | No. Observed | SMR(95%CI) | No. Observed | SMR(95%CI) | No. Observed | SMR(95%CI) | No. Observed | SMR(95%CI) |  |  |  |  |
| All cause of death | 31981 | 25.78#(25.49-26.06) | 35717 | 19.17#(18.97-19.37) | 2961 | 4.79#(4.62-4.96) | 419 | 1.95#(1.77-2.15) | 71078 | 18.06#(17.92-18.19) |  |  |  |  |
| All malignant cancers | 29881 | 100.75#(99.61-101.9) | 33930 | 73.77#(72.99-74.56) | 2425 | 17.44#(16.76-18.15) | 211 | 4.71#(4.09-5.39) | 66447 | 70.66#(70.12-71.2) |  |  |  |  |
| Colon and Rectum | 27422 | 971.77#(960.3-983.34) | 32036 | 755.87#(747.61-764.19) | 2143 | 170.24#(163.11-177.6) | 127 | 31.87#(26.57-37.92) | 61728 | 708.10#(702.52-713.7) |  |  |  |  |
| In situ, benign, or unknown behavior neoplasms | 57 | 7.55#(5.72-9.78) | 57 | 4.97#(3.76-6.44) | 9 | 2.29#(1.05-4.34) | 3 | 2.16(0.44-6.3) | 126 | 5.17#(4.31-6.16) |  |  |  |  |
| Tuberculosis | 0 | 0(0-10.13) | 0 | 0(0-7.06) | 0 | 0(0-25.6) | 0 | 0(0-85.01) | 0 | 0(0-3.43) |  |  |  |  |
| syphilis | 0 | 0(0-169.65) | 0 | 0(0-118.6) | 0 | 0(0-391.08) | 0 | 0(0-1112.04) | 0 | 0(0-56.24) |  |  |  |  |
| Septicemia | 104 | 5.63#(4.6-6.82) | 89 | 3.19#(2.56-3.93) | 14 | 1.52(0.83-2.55) | 4 | 1.25(0.34-3.2) | 211 | 3.59#(3.12-4.11) |  |  |  |  |
| Other infectious diseases | 51 | 4.39#(3.27-5.77) | 43 | 2.36#(1.71-3.18) | 3 | 0.56(0.12-1.64) | 5 | 3.01(0.98-7.03) | 102 | 2.77#(2.26-3.36) |  |  |  |  |
| Diabetes Mellitus | 46 | 1.17(0.86-1.56) | 52 | 0.87(0.65-1.14) | 22 | 1.2(0.75-1.81) | 3 | 0.49(0.1-1.43) | 123 | 1(0.83-1.19) |  |  |  |  |
| Alzheimers | 34 | 0.85(0.59-1.18) | 29 | 0.49#(0.33-0.7) | 26 | 1(0.65-1.46) | 15 | 1.37(0.77-2.27) | 104 | 0.76#(0.62-0.92) |  |  |  |  |
| Diseasis of hearts | 810 | 2.38#(2.22-2.55) | 610 | 1.24#(1.14-1.34) | 165 | 1.02(0.87-1.19) | 74 | 1.34#(1.05-1.68) | 1659 | 1.58#(1.5-1.66) |  |  |  |  |
| Hypertension without heart disease | 34 | 2.47#(1.71-3.45) | 22 | 1.06(0.67-1.61) | 7 | 0.9(0.36-1.86) | 1 | 0.34(0.01-1.91) | 64 | 1.42#(1.09-1.81) |  |  |  |  |
| Cerebrovascular diseases | 133 | 1.74#(1.45-2.06) | 114 | 1.05(0.87-1.26) | 36 | 0.99(0.69-1.37) | 10 | 0.78(0.37-1.43) | 293 | 1.25#(1.11-1.4) |  |  |  |  |
| Atherosclerosis | 17 | 3.36#(1.96-5.37) | 6 | 0.91(0.34-1.99) | 1 | 0.49(0.01-2.71) | 1 | 1.54(0.04-8.56) | 25 | 1.74#(1.13-2.58) |  |  |  |  |
| Aortic aneurysm and dissection | 11 | 1.64(0.82-2.94) | 5 | 0.52(0.17-1.22) | 5 | 1.84(0.6-4.29) | 1 | 1.21(0.03-6.73) | 22 | 1.11(0.69-1.68) |  |  |  |  |
| Other diseases of arteries, arterioles, capillaries | 12 | 2.37#(1.22-4.14) | 8 | 1.09(0.47-2.15) | 3 | 1.23(0.25-3.59) | 0 | 0(0-4.33) | 23 | 1.47(0.93-2.2) |  |  |  |  |
| Pneumonia and influenza | 60 | 1.95#(1.49-2.51) | 46 | 1.06(0.78-1.41) | 17 | 1.14(0.66-1.82) | 11 | 2.15#(1.07-3.85) | 134 | 1.42#(1.19-1.68) |  |  |  |  |
| Chronic obstructive pulmonary disease and allied cond | 118 | 1.62#(1.34-1.94) | 92 | 0.82(0.66-1) | 50 | 1.31(0.97-1.73) | 12 | 0.9(0.47-1.58) | 272 | 1.15#(1.01-1.29) |  |  |  |  |
| Stomach and duodenal ulcers | 9 | 5.00#(2.29-9.49) | 6 | 2.34(0.86-5.09) | 2 | 2.57(0.31-9.28) | 1 | 3.94(0.1-21.93) | 18 | 3.33#(1.98-5.27) |  |  |  |  |
| Chronic liver disease and cirrhosis | 27 | 2.07#(1.36-3.01) | 14 | 0.65(0.35-1.09) | 2 | 0.34(0.04-1.24) | 1 | 0.58(0.01-3.24) | 44 | 1.04(0.76-1.4) |  |  |  |  |
| Nephritis, Nephrotic Syndrome and Nephrosis | 49 | 1.97#(1.45-2.6) | 49 | 1.31(0.97-1.74) | 10 | 0.78(0.38-1.44) | 7 | 1.57(0.63-3.24) | 115 | 1.45#(1.19-1.74) |  |  |  |  |
| Complications of pregnancy, childbirth, puerperium | 5 | 122.58#(39.8-286.05) | 14 | 200.27#(109.49-336.02) | 2 | 143.15#(17.34-517.12) | 0 | 0(0-1183.88) | 21 | 164.34#(101.73-251.21) |  |  |  |  |
| Congenital anomalies | 1 | 0.84(0.02-4.66) | 0 | 0(0-1.96) | 0 | 0(0-7.14) | 0 | 0(0-23.69) | 1 | 0.27(0.01-1.48) |  |  |  |  |
| Certain conditions originating in perinatal period | 0 | 0(0-725.31) | 1 | 123.13#(3.12-686.05) | 0 | 0(0-1827.5) | 0 | 0(0-6671.98) | 1 | 63.38#(1.6-353.11) |  |  |  |  |
| Symptoms, Signs and Ill-Defined Conditions | 55 | 3.93#(2.96-5.11) | 54 | 2.60#(1.95-3.39) | 11 | 1.38(0.69-2.47) | 3 | 1.09(0.22-3.18) | 123 | 2.70#(2.25-3.22) |  |  |  |  |
| Accidents and Adverse Effects | 66 | 1.87#(1.44-2.37) | 71 | 1.27(0.99-1.61) | 21 | 1.21(0.75-1.84) | 9 | 1.53(0.7-2.91) | 167 | 1.46#(1.25-1.7) |  |  |  |  |
| Suicide and Self-Inflicted Injury | 35 | 3.95#(2.75-5.49) | 38 | 2.60#(1.84-3.57) | 5 | 1.33(0.43-3.1) | 1 | 0.95(0.02-5.27) | 79 | 2.79#(2.21-3.48) |  |  |  |  |
| Homicide and legal intervention | 1 | 0.48(0.01-2.7) | 2 | 0.64(0.08-2.32) | 0 | 0(0-5.76) | 0 | 0(0-23.11) | 3 | 0.5(0.1-1.47) |  |  |  |  |
| Other Cause of Death | 365 | 2.10#(1.89-2.33) | 365 | 1.37#(1.23-1.52) | 125 | 1.24#(1.03-1.48) | 46 | 1.21(0.89-1.62) | 901 | 1.56#(1.46-1.66) |  |  |  |  |

# P value less than .05

Supplementary Table 10. Standardized-mortality ratios (SMRs) for each cause of death following CRC diagnosis in regional stage patients.

| Timing of Deaths After Diagnosis | | | | | | | | | | |  |  |  |  |
| --- | --- | --- | --- | --- | --- | --- | --- | --- | --- | --- | --- | --- | --- | --- |
| Cause of death | <1years | | 1-5years | | 5-10years | | >10years | | Total | |  |  |  |  |
| Selected Events | No. Observed | SMR(95%CI) | No. Observed | SMR(95%CI) | No. Observed | SMR(95%CI) | No. Observed | SMR(95%CI) | No. Observed | SMR(95%CI) |  |  |  |  |
| All cause of death | 14169 | 3.58#(3.52-3.64) | 40669 | 2.91#(2.88-2.93) | 17448 | 1.78#(1.75-1.81) | 5616 | 1.34#(1.3-1.37) | 77902 | 2.44#(2.42-2.45) |  |  |  |  |
| All malignant cancers | 9865 | 11.35#(11.13-11.58) | 30542 | 10.17#(10.05-10.28) | 9030 | 4.54#(4.45-4.64) | 1712 | 2.13#(2.03-2.24) | 51149 | 7.68#(7.61-7.74) |  |  |  |  |
| Colon and Rectum | 9179 | 107.45#(105.26-109.67) | 27345 | 94.30#(93.19-95.43) | 6929 | 37.22#(36.35-38.11) | 817 | 11.21#(10.45-12) | 44270 | 69.78#(69.13-70.43) |  |  |  |  |
| In situ, benign, or unknown behavior neoplasms | 28 | 1.14(0.76-1.65) | 123 | 1.40#(1.16-1.66) | 85 | 1.34#(1.07-1.65) | 30 | 1.08(0.73-1.54) | 266 | 1.30#(1.15-1.47) |  |  |  |  |
| Tuberculosis | 3 | 2.76(0.57-8.06) | 2 | 0.56(0.07-2.02) | 2 | 0.92(0.11-3.31) | 4 | 4.87#(1.33-12.48) | 11 | 1.43(0.72-2.57) |  |  |  |  |
| syphilis | 0 | 0(0-61.82) | 0 | 0(0-18.15) | 1 | 7.33(0.19-40.86) | 0 | 0(0-63.46) | 1 | 2.19(0.06-12.18) |  |  |  |  |
| Septicemia | 194 | 3.37#(2.91-3.88) | 277 | 1.36#(1.2-1.53) | 159 | 1.12(0.95-1.3) | 67 | 1.1(0.85-1.4) | 697 | 1.50#(1.39-1.62) |  |  |  |  |
| Other infectious diseases | 85 | 2.76#(2.21-3.42) | 108 | 0.98(0.81-1.19) | 76 | 1.01(0.79-1.26) | 28 | 0.94(0.62-1.36) | 297 | 1.21#(1.07-1.35) |  |  |  |  |
| Diabetes Mellitus | 167 | 1.42#(1.21-1.65) | 429 | 1.05(0.95-1.16) | 324 | 1.19#(1.06-1.32) | 157 | 1.40#(1.19-1.64) | 1077 | 1.18#(1.11-1.26) |  |  |  |  |
| Alzheimers | 64 | 0.42#(0.33-0.54) | 364 | 0.63#(0.56-0.69) | 509 | 1.07(0.98-1.16) | 338 | 1.45#(1.3-1.61) | 1275 | 0.88#(0.84-0.93) |  |  |  |  |
| Diseasis of hearts | 1630 | 1.44#(1.37-1.51) | 3732 | 0.95#(0.92-0.98) | 2970 | 1.11#(1.07-1.15) | 1276 | 1.14#(1.08-1.21) | 9608 | 1.09#(1.07-1.11) |  |  |  |  |
| Hypertension without heart disease | 48 | 1.06(0.78-1.41) | 159 | 0.95(0.81-1.11) | 161 | 1.25#(1.06-1.46) | 69 | 1.18(0.92-1.49) | 437 | 1.09(0.99-1.2) |  |  |  |  |
| Cerebrovascular diseases | 360 | 1.36#(1.23-1.51) | 828 | 0.91#(0.85-0.98) | 641 | 1.05(0.97-1.13) | 269 | 1.03(0.91-1.17) | 2098 | 1.03(0.98-1.07) |  |  |  |  |
| Atherosclerosis | 30 | 1.57#(1.06-2.24) | 61 | 0.99(0.76-1.27) | 36 | 0.97(0.68-1.35) | 25 | 1.80#(1.16-2.65) | 152 | 1.15(0.98-1.35) |  |  |  |  |
| Aortic aneurysm and dissection | 27 | 1.25(0.82-1.82) | 58 | 0.82(0.62-1.05) | 35 | 0.82(0.57-1.13) | 13 | 0.81(0.43-1.39) | 133 | 0.88(0.73-1.04) |  |  |  |  |
| Other diseases of arteries, arterioles, capillaries | 21 | 1.25(0.77-1.91) | 64 | 1.1(0.85-1.4) | 52 | 1.3(0.97-1.71) | 20 | 1.19(0.73-1.84) | 157 | 1.19#(1.01-1.39) |  |  |  |  |
| Pneumonia and influenza | 154 | 1.42#(1.2-1.66) | 329 | 0.87#(0.78-0.97) | 276 | 1.08(0.95-1.21) | 127 | 1.19(0.99-1.42) | 886 | 1.04(0.98-1.11) |  |  |  |  |
| Chronic obstructive pulmonary disease and allied cond | 285 | 1.22#(1.08-1.36) | 782 | 0.93(0.87-1) | 619 | 1.04(0.96-1.13) | 242 | 0.95(0.84-1.08) | 1928 | 1.01(0.96-1.05) |  |  |  |  |
| Stomach and duodenal ulcers | 12 | 2.02#(1.04-3.53) | 19 | 0.97(0.58-1.51) | 27 | 2.19#(1.45-3.19) | 6 | 1.22(0.45-2.65) | 64 | 1.49#(1.15-1.91) |  |  |  |  |
| Chronic liver disease and cirrhosis | 56 | 1.75#(1.32-2.27) | 100 | 0.92(0.75-1.12) | 86 | 1.28#(1.02-1.58) | 36 | 1.39(0.97-1.92) | 278 | 1.19#(1.05-1.34) |  |  |  |  |
| Nephritis, Nephrotic Syndrome and Nephrosis | 120 | 1.50#(1.25-1.8) | 268 | 0.93(0.82-1.05) | 234 | 1.13(0.99-1.28) | 98 | 1.11(0.9-1.35) | 720 | 1.08#(1.01-1.17) |  |  |  |  |
| Complications of pregnancy, childbirth, puerperium | 2 | 29.73#(3.6-107.41) | 1 | 4.76(0.12-26.53) | 0 | 0(0-39.1) | 0 | 0(0-156.71) | 3 | 7.59#(1.57-22.18) |  |  |  |  |
| Congenital anomalies | 7 | 2.21(0.89-4.55) | 7 | 0.66(0.27-1.36) | 4 | 0.61(0.17-1.56) | 0 | 0(0-1.44) | 18 | 0.79(0.47-1.24) |  |  |  |  |
| Certain conditions originating in perinatal period | 0 | 0(0-302.18) | 0 | 0(0-89.47) | 1 | 42.21#(1.07-235.2) | 0 | 0(0-422.62) | 1 | 11.65(0.29-64.9) |  |  |  |  |
| Symptoms, Signs and Ill-Defined Conditions | 58 | 1.23(0.93-1.58) | 181 | 1.02(0.88-1.18) | 148 | 1.07(0.91-1.26) | 59 | 1.02(0.78-1.31) | 446 | 1.06(0.96-1.16) |  |  |  |  |
| Accidents and Adverse Effects | 111 | 1.1(0.9-1.32) | 287 | 0.79#(0.7-0.89) | 237 | 0.92(0.81-1.05) | 116 | 1.04(0.86-1.24) | 751 | 0.90#(0.84-0.97) |  |  |  |  |
| Suicide and Self-Inflicted Injury | 34 | 1.61#(1.12-2.25) | 106 | 1.48#(1.21-1.79) | 58 | 1.32#(1-1.71) | 14 | 0.84(0.46-1.41) | 212 | 1.38#(1.2-1.58) |  |  |  |  |
| Homicide and legal intervention | 5 | 1.16(0.38-2.7) | 15 | 1.12(0.63-1.85) | 4 | 0.58(0.16-1.48) | 7 | 3.05#(1.22-6.28) | 31 | 1.15(0.78-1.63) |  |  |  |  |
| Other Cause of Death | 803 | 1.40#(1.31-1.5) | 1827 | 0.85#(0.81-0.89) | 1673 | 1(0.95-1.05) | 903 | 1.17#(1.1-1.25) | 5206 | 1.01(0.98-1.04) |  |  |  |  |

# P value less than .05

Supplementary Table 11. Standardized-mortality ratios (SMRs) for each cause of death following CRC diagnosis in localsized stage patients.

| Timing of Deaths After Diagnosis | | | | | | | | | | |  |  |  |  |
| --- | --- | --- | --- | --- | --- | --- | --- | --- | --- | --- | --- | --- | --- | --- |
| Cause of death | <1years | | 1-5years | | 5-10years | | >10years | | Total | |  |  |  |  |
| Selected Events | No. Observed | SMR(95%CI) | No. Observed | SMR(95%CI) | No. Observed | SMR(95%CI) | No. Observed | SMR(95%CI) | No. Observed | SMR(95%CI) |  |  |  |  |
| All cause of death | 9614 | 1.94#(1.9-1.98) | 28539 | 1.43#(1.41-1.44) | 20605 | 1.31#(1.29-1.33) | 8440 | 1.23#(1.2-1.26) | 67198 | 1.41#(1.4-1.42) |  |  |  |  |
| All malignant cancers | 4455 | 4.10#(3.98-4.22) | 13381 | 3.14#(3.08-3.19) | 6922 | 2.17#(2.12-2.23) | 2024 | 1.55#(1.48-1.62) | 26782 | 2.72#(2.69-2.75) |  |  |  |  |
| Colon and Rectum | 3774 | 35.44#(34.32-36.59) | 9617 | 23.39#(22.92-23.86) | 3723 | 12.53#(12.13-12.94) | 719 | 6.07#(5.63-6.53) | 17833 | 19.11#(18.83-19.39) |  |  |  |  |
| In situ, benign, or unknown behavior neoplasms | 34 | 1.1(0.76-1.54) | 132 | 1.04(0.87-1.24) | 105 | 1.02(0.84-1.24) | 47 | 1.03(0.75-1.37) | 318 | 1.04(0.93-1.16) |  |  |  |  |
| Tuberculosis | 2 | 1.54(0.19-5.55) | 8 | 1.65(0.71-3.24) | 2 | 0.6(0.07-2.15) | 2 | 1.54(0.19-5.57) | 14 | 1.29(0.71-2.17) |  |  |  |  |
| syphilis | 0 | 0(0-50.01) | 0 | 0(0-12.86) | 0 | 0(0-17.03) | 0 | 0(0-40.28) | 0 | 0(0-5.52) |  |  |  |  |
| Septicemia | 183 | 2.54#(2.19-2.94) | 346 | 1.19#(1.07-1.32) | 268 | 1.18#(1.04-1.32) | 106 | 1.07(0.88-1.3) | 903 | 1.31#(1.22-1.4) |  |  |  |  |
| Other infectious diseases | 104 | 2.74#(2.24-3.32) | 156 | 1.01(0.86-1.18) | 116 | 0.97(0.8-1.16) | 41 | 0.85(0.61-1.16) | 417 | 1.16#(1.05-1.28) |  |  |  |  |
| Diabetes Mellitus | 206 | 1.41#(1.22-1.61) | 691 | 1.20#(1.11-1.29) | 579 | 1.33#(1.22-1.44) | 263 | 1.45#(1.28-1.63) | 1739 | 1.30#(1.24-1.36) |  |  |  |  |
| Alzheimers | 85 | 0.45#(0.36-0.56) | 563 | 0.67#(0.61-0.72) | 824 | 1.08#(1-1.15) | 489 | 1.28#(1.17-1.4) | 1961 | 0.90#(0.86-0.94) |  |  |  |  |
| Diseasis of hearts | 1995 | 1.41#(1.35-1.47) | 5693 | 1.01(0.99-1.04) | 4847 | 1.13#(1.1-1.16) | 2187 | 1.19#(1.14-1.24) | 14722 | 1.12#(1.1-1.14) |  |  |  |  |
| Hypertension without heart disease | 80 | 1.42#(1.13-1.77) | 245 | 1.02(0.9-1.16) | 256 | 1.25#(1.1-1.41) | 127 | 1.34#(1.12-1.6) | 708 | 1.19#(1.1-1.28) |  |  |  |  |
| Cerebrovascular diseases | 400 | 1.22#(1.1-1.35) | 1173 | 0.91#(0.86-0.96) | 965 | 0.99(0.93-1.05) | 452 | 1.07(0.97-1.17) | 2990 | 0.99(0.96-1.03) |  |  |  |  |
| Atherosclerosis | 29 | 1.22(0.82-1.76) | 98 | 1.11(0.9-1.35) | 56 | 0.94(0.71-1.22) | 29 | 1.27(0.85-1.82) | 212 | 1.09(0.95-1.25) |  |  |  |  |
| Aortic aneurysm and dissection | 32 | 1.18(0.81-1.67) | 90 | 0.89(0.71-1.09) | 71 | 1.03(0.8-1.3) | 32 | 1.22(0.83-1.72) | 225 | 1.01(0.88-1.15) |  |  |  |  |
| Other diseases of arteries, arterioles, capillaries | 37 | 1.76#(1.24-2.43) | 63 | 0.75#(0.58-0.97) | 69 | 1.08(0.84-1.36) | 35 | 1.28(0.89-1.77) | 204 | 1.04(0.9-1.19) |  |  |  |  |
| Pneumonia and influenza | 170 | 1.26#(1.08-1.46) | 461 | 0.86#(0.78-0.94) | 500 | 1.21#(1.11-1.33) | 193 | 1.1(0.95-1.27) | 1324 | 1.05(0.99-1.11) |  |  |  |  |
| Chronic obstructive pulmonary disease and allied cond | 355 | 1.20#(1.08-1.33) | 1279 | 1.06#(1.01-1.12) | 1055 | 1.10#(1.04-1.17) | 456 | 1.09(0.99-1.2) | 3145 | 1.09#(1.06-1.13) |  |  |  |  |
| Stomach and duodenal ulcers | 10 | 1.36(0.65-2.5) | 24 | 0.86(0.55-1.28) | 14 | 0.71(0.39-1.2) | 4 | 0.5(0.14-1.28) | 52 | 0.83(0.62-1.09) |  |  |  |  |
| Chronic liver disease and cirrhosis | 114 | 2.89#(2.38-3.47) | 218 | 1.45#(1.27-1.66) | 152 | 1.45#(1.23-1.69) | 51 | 1.24(0.92-1.63) | 535 | 1.59#(1.46-1.73) |  |  |  |  |
| Nephritis, Nephrotic Syndrome and Nephrosis | 173 | 1.73#(1.48-2.01) | 481 | 1.16#(1.06-1.27) | 438 | 1.31#(1.19-1.44) | 167 | 1.15(0.98-1.34) | 1259 | 1.27#(1.2-1.34) |  |  |  |  |
| Complications of pregnancy, childbirth, puerperium | 0 | 0(0-48.27) | 0 | 0(0-14.76) | 0 | 0(0-30.87) | 1 | 28.83(0.73-160.6) | 1 | 2.08(0.05-11.59) |  |  |  |  |
| Congenital anomalies | 5 | 1.29(0.42-3.01) | 12 | 0.82(0.42-1.43) | 11 | 1.08(0.54-1.92) | 3 | 0.73(0.15-2.14) | 31 | 0.94(0.64-1.34) |  |  |  |  |
| Certain conditions originating in perinatal period | 0 | 0(0-258.72) | 0 | 0(0-69.27) | 0 | 0(0-101.14) | 0 | 0(0-264.8) | 0 | 0(0-31.28) |  |  |  |  |
| Symptoms, Signs and Ill-Defined Conditions | 83 | 1.40#(1.12-1.74) | 228 | 0.9(0.78-1.02) | 219 | 0.99(0.87-1.14) | 83 | 0.88(0.7-1.09) | 613 | 0.98(0.9-1.06) |  |  |  |  |
| Accidents and Adverse Effects | 106 | 0.85(0.7-1.03) | 477 | 0.94(0.86-1.03) | 391 | 0.96(0.87-1.06) | 196 | 1.07(0.93-1.24) | 1170 | 0.96(0.9-1.01) |  |  |  |  |
| Suicide and Self-Inflicted Injury | 37 | 1.44#(1.02-1.99) | 115 | 1.18(0.97-1.42) | 73 | 1.07(0.84-1.34) | 33 | 1.22(0.84-1.71) | 258 | 1.18#(1.04-1.33) |  |  |  |  |
| Homicide and legal intervention | 5 | 1(0.32-2.33) | 21 | 1.21(0.75-1.84) | 16 | 1.54(0.88-2.51) | 1 | 0.29(0.01-1.59) | 43 | 1.19(0.86-1.6) |  |  |  |  |
| Other Cause of Death | 914 | 1.27#(1.19-1.36) | 2584 | 0.84#(0.81-0.87) | 2656 | 0.99(0.95-1.03) | 1418 | 1.12#(1.07-1.18) | 7572 | 0.98#(0.95-1) |  |  |  |  |

# P value less than .05

Supplementary Table 12. Standardized-mortality ratios (SMRs) for each cause of death following CRC diagnosis in patients in grade I.

| Timing of Deaths After Diagnosis | | | | | | | | | | |  |  |  |  |
| --- | --- | --- | --- | --- | --- | --- | --- | --- | --- | --- | --- | --- | --- | --- |
| Cause of death | <1years | | 1-5years | | 5-10years | | >10years | | Total | |  |  |  |  |
| Selected Events | No. Observed | SMR(95%CI) | No. Observed | SMR(95%CI) | No. Observed | SMR(95%CI) | No. Observed | SMR(95%CI) | No. Observed | SMR(95%CI) |  |  |  |  |
| All cause of death | 2987 | 3.02#(2.91-3.13) | 7424 | 1.98#(1.93-2.02) | 4126 | 1.39#(1.35-1.43) | 1649 | 1.25#(1.19-1.31) | 16186 | 1.79#(1.77-1.82) |  |  |  |  |
| All malignant cancers | 1940 | 8.64#(8.26-9.04) | 4558 | 5.52#(5.36-5.68) | 1612 | 2.64#(2.51-2.77) | 435 | 1.71#(1.55-1.88) | 8545 | 4.46#(4.36-4.55) |  |  |  |  |
| Colon and Rectum | 1741 | 79.87#(76.17-83.72) | 3719 | 47.05#(45.55-48.58) | 990 | 17.45#(16.38-18.57) | 153 | 6.67#(5.66-7.82) | 6603 | 36.58#(35.7-37.47) |  |  |  |  |
| In situ, benign, or unknown behavior neoplasms | 6 | 0.98(0.36-2.14) | 31 | 1.31(0.89-1.86) | 11 | 0.57(0.28-1.01) | 11 | 1.25(0.62-2.23) | 59 | 1.02(0.77-1.31) |  |  |  |  |
| Tuberculosis | 0 | 0(0-14.11) | 3 | 3.31(0.68-9.66) | 1 | 1.63(0.04-9.07) | 0 | 0(0-15.61) | 4 | 1.98(0.54-5.07) |  |  |  |  |
| syphilis | 0 | 0(0-239.05) | 0 | 0(0-66.51) | 0 | 0(0-89.14) | 0 | 0(0-209.34) | 0 | 0(0-28.4) |  |  |  |  |
| Septicemia | 36 | 2.48#(1.74-3.44) | 76 | 1.38#(1.09-1.73) | 46 | 1.06(0.78-1.42) | 25 | 1.31(0.85-1.93) | 183 | 1.39#(1.19-1.6) |  |  |  |  |
| Other infectious diseases | 23 | 2.87#(1.82-4.31) | 41 | 1.37(0.98-1.86) | 20 | 0.88(0.54-1.36) | 10 | 1.08(0.52-1.99) | 94 | 1.35#(1.09-1.65) |  |  |  |  |
| Diabetes Mellitus | 50 | 1.67#(1.24-2.2) | 118 | 1.07(0.88-1.28) | 98 | 1.18(0.96-1.44) | 58 | 1.65#(1.25-2.13) | 324 | 1.25#(1.12-1.4) |  |  |  |  |
| Alzheimers | 21 | 0.59#(0.37-0.91) | 98 | 0.66#(0.53-0.8) | 130 | 0.93(0.77-1.1) | 88 | 1.22(0.98-1.51) | 337 | 0.85#(0.76-0.94) |  |  |  |  |
| Diseasis of hearts | 411 | 1.47#(1.33-1.62) | 1083 | 1.03(0.97-1.1) | 891 | 1.10#(1.03-1.18) | 374 | 1.06(0.96-1.18) | 2759 | 1.11#(1.07-1.15) |  |  |  |  |
| Hypertension without heart disease | 20 | 1.82#(1.11-2.81) | 48 | 1.09(0.81-1.45) | 54 | 1.42#(1.06-1.85) | 31 | 1.73#(1.17-2.45) | 153 | 1.38#(1.17-1.62) |  |  |  |  |
| Cerebrovascular diseases | 67 | 1.04(0.81-1.33) | 248 | 1.04(0.91-1.18) | 206 | 1.13(0.98-1.3) | 85 | 1.06(0.84-1.3) | 606 | 1.07(0.99-1.16) |  |  |  |  |
| Atherosclerosis | 7 | 1.53(0.62-3.15) | 16 | 0.99(0.56-1.6) | 5 | 0.45(0.15-1.05) | 5 | 1.15(0.37-2.69) | 33 | 0.91(0.63-1.28) |  |  |  |  |
| Aortic aneurysm and dissection | 5 | 0.92(0.3-2.14) | 17 | 0.88(0.51-1.41) | 11 | 0.84(0.42-1.5) | 11 | 2.17#(1.08-3.88) | 44 | 1.02(0.74-1.37) |  |  |  |  |
| Other diseases of arteries, arterioles, capillaries | 4 | 0.96(0.26-2.46) | 17 | 1.09(0.64-1.75) | 10 | 0.83(0.4-1.52) | 4 | 0.76(0.21-1.94) | 35 | 0.94(0.66-1.31) |  |  |  |  |
| Pneumonia and influenza | 33 | 1.26(0.87-1.77) | 78 | 0.79#(0.62-0.98) | 95 | 1.24(1-1.51) | 38 | 1.14(0.81-1.57) | 244 | 1.04(0.91-1.18) |  |  |  |  |
| Chronic obstructive pulmonary disease and allied cond | 68 | 1.15(0.9-1.46) | 249 | 1.1(0.97-1.25) | 197 | 1.09(0.94-1.25) | 81 | 1(0.8-1.25) | 595 | 1.09#(1-1.18) |  |  |  |  |
| Stomach and duodenal ulcers | 3 | 2.04(0.42-5.97) | 4 | 0.77(0.21-1.96) | 2 | 0.54(0.07-1.96) | 2 | 1.31(0.16-4.72) | 11 | 0.92(0.46-1.65) |  |  |  |  |
| Chronic liver disease and cirrhosis | 16 | 1.82#(1.04-2.96) | 37 | 1.21(0.85-1.67) | 27 | 1.31(0.86-1.91) | 13 | 1.61(0.86-2.75) | 93 | 1.37#(1.1-1.67) |  |  |  |  |
| Nephritis, Nephrotic Syndrome and Nephrosis | 33 | 1.66#(1.14-2.33) | 78 | 1.01(0.8-1.26) | 83 | 1.31#(1.05-1.63) | 32 | 1.14(0.78-1.62) | 226 | 1.20#(1.05-1.37) |  |  |  |  |
| Complications of pregnancy, childbirth, puerperium | 0 | 0(0-145.94) | 0 | 0(0-56.27) | 0 | 0(0-149.78) | 0 | 0(0-573.93) | 0 | 0(0-30.26) |  |  |  |  |
| Congenital anomalies | 1 | 1.19(0.03-6.61) | 4 | 1.37(0.37-3.51) | 2 | 1.01(0.12-3.67) | 1 | 1.26(0.03-7.05) | 8 | 1.23(0.53-2.42) |  |  |  |  |
| Certain conditions originating in perinatal period | 0 | 0(0-1108.78) | 0 | 0(0-332.83) | 0 | 0(0-511.92) | 0 | 0(0-1325.73) | 0 | 0(0-151.19) |  |  |  |  |
| Symptoms, Signs and Ill-Defined Conditions | 16 | 1.4(0.8-2.28) | 41 | 0.89(0.64-1.2) | 37 | 0.9(0.64-1.25) | 16 | 0.89(0.51-1.45) | 110 | 0.94(0.78-1.14) |  |  |  |  |
| Accidents and Adverse Effects | 26 | 1(0.65-1.46) | 78 | 0.80#(0.63-1) | 71 | 0.92(0.72-1.16) | 46 | 1.32(0.96-1.76) | 221 | 0.94(0.82-1.07) |  |  |  |  |
| Suicide and Self-Inflicted Injury | 8 | 1.37(0.59-2.7) | 23 | 1.14(0.72-1.71) | 21 | 1.55(0.96-2.38) | 5 | 0.93(0.3-2.18) | 57 | 1.27(0.96-1.65) |  |  |  |  |
| Homicide and legal intervention | 1 | 0.82(0.02-4.6) | 5 | 1.34(0.43-3.13) | 2 | 0.99(0.12-3.58) | 0 | 0(0-5.48) | 8 | 1.05(0.45-2.06) |  |  |  |  |
| Other Cause of Death | 192 | 1.37#(1.18-1.57) | 473 | 0.84#(0.76-0.91) | 494 | 0.98(0.9-1.07) | 278 | 1.15#(1.02-1.3) | 1437 | 0.99(0.94-1.04) |  |  |  |  |

# P value less than .05

Supplementary Table 13. Standardized-mortality ratios (SMRs) for each cause of death following CRC diagnosis in patients in grade II.

| Timing of Deaths After Diagnosis | | | | | | | | | | |  |  |  |  |
| --- | --- | --- | --- | --- | --- | --- | --- | --- | --- | --- | --- | --- | --- | --- |
| Cause of death | <1years | | 1-5years | | 5-10years | | >10years | | Total | |  |  |  |  |
| Selected Events | No. Observed | SMR(95%CI) | No. Observed | SMR(95%CI) | No. Observed | SMR(95%CI) | No. Observed | SMR(95%CI) | No. Observed | SMR(95%CI) |  |  |  |  |
| All cause of death | 2987 | 3.02#(2.91-3.13) | 7424 | 1.98#(1.93-2.02) | 4126 | 1.39#(1.35-1.43) | 1649 | 1.25#(1.19-1.31) | 16186 | 1.79#(1.77-1.82) |  |  |  |  |
| All malignant cancers | 1940 | 8.64#(8.26-9.04) | 4558 | 5.52#(5.36-5.68) | 1612 | 2.64#(2.51-2.77) | 435 | 1.71#(1.55-1.88) | 8545 | 4.46#(4.36-4.55) |  |  |  |  |
| Colon and Rectum | 1741 | 79.87#(76.17-83.72) | 3719 | 47.05#(45.55-48.58) | 990 | 17.45#(16.38-18.57) | 153 | 6.67#(5.66-7.82) | 6603 | 36.58#(35.7-37.47) |  |  |  |  |
| In situ, benign, or unknown behavior neoplasms | 6 | 0.98(0.36-2.14) | 31 | 1.31(0.89-1.86) | 11 | 0.57(0.28-1.01) | 11 | 1.25(0.62-2.23) | 59 | 1.02(0.77-1.31) |  |  |  |  |
| Tuberculosis | 0 | 0(0-14.11) | 3 | 3.31(0.68-9.66) | 1 | 1.63(0.04-9.07) | 0 | 0(0-15.61) | 4 | 1.98(0.54-5.07) |  |  |  |  |
| syphilis | 0 | 0(0-239.05) | 0 | 0(0-66.51) | 0 | 0(0-89.14) | 0 | 0(0-209.34) | 0 | 0(0-28.4) |  |  |  |  |
| Septicemia | 36 | 2.48#(1.74-3.44) | 76 | 1.38#(1.09-1.73) | 46 | 1.06(0.78-1.42) | 25 | 1.31(0.85-1.93) | 183 | 1.39#(1.19-1.6) |  |  |  |  |
| Other infectious diseases | 23 | 2.87#(1.82-4.31) | 41 | 1.37(0.98-1.86) | 20 | 0.88(0.54-1.36) | 10 | 1.08(0.52-1.99) | 94 | 1.35#(1.09-1.65) |  |  |  |  |
| Diabetes Mellitus | 50 | 1.67#(1.24-2.2) | 118 | 1.07(0.88-1.28) | 98 | 1.18(0.96-1.44) | 58 | 1.65#(1.25-2.13) | 324 | 1.25#(1.12-1.4) |  |  |  |  |
| Alzheimers | 21 | 0.59#(0.37-0.91) | 98 | 0.66#(0.53-0.8) | 130 | 0.93(0.77-1.1) | 88 | 1.22(0.98-1.51) | 337 | 0.85#(0.76-0.94) |  |  |  |  |
| Diseasis of hearts | 411 | 1.47#(1.33-1.62) | 1083 | 1.03(0.97-1.1) | 891 | 1.10#(1.03-1.18) | 374 | 1.06(0.96-1.18) | 2759 | 1.11#(1.07-1.15) |  |  |  |  |
| Hypertension without heart disease | 20 | 1.82#(1.11-2.81) | 48 | 1.09(0.81-1.45) | 54 | 1.42#(1.06-1.85) | 31 | 1.73#(1.17-2.45) | 153 | 1.38#(1.17-1.62) |  |  |  |  |
| Cerebrovascular diseases | 67 | 1.04(0.81-1.33) | 248 | 1.04(0.91-1.18) | 206 | 1.13(0.98-1.3) | 85 | 1.06(0.84-1.3) | 606 | 1.07(0.99-1.16) |  |  |  |  |
| Atherosclerosis | 7 | 1.53(0.62-3.15) | 16 | 0.99(0.56-1.6) | 5 | 0.45(0.15-1.05) | 5 | 1.15(0.37-2.69) | 33 | 0.91(0.63-1.28) |  |  |  |  |
| Aortic aneurysm and dissection | 5 | 0.92(0.3-2.14) | 17 | 0.88(0.51-1.41) | 11 | 0.84(0.42-1.5) | 11 | 2.17#(1.08-3.88) | 44 | 1.02(0.74-1.37) |  |  |  |  |
| Other diseases of arteries, arterioles, capillaries | 4 | 0.96(0.26-2.46) | 17 | 1.09(0.64-1.75) | 10 | 0.83(0.4-1.52) | 4 | 0.76(0.21-1.94) | 35 | 0.94(0.66-1.31) |  |  |  |  |
| Pneumonia and influenza | 33 | 1.26(0.87-1.77) | 78 | 0.79#(0.62-0.98) | 95 | 1.24(1-1.51) | 38 | 1.14(0.81-1.57) | 244 | 1.04(0.91-1.18) |  |  |  |  |
| Chronic obstructive pulmonary disease and allied cond | 68 | 1.15(0.9-1.46) | 249 | 1.1(0.97-1.25) | 197 | 1.09(0.94-1.25) | 81 | 1(0.8-1.25) | 595 | 1.09#(1-1.18) |  |  |  |  |
| Stomach and duodenal ulcers | 3 | 2.04(0.42-5.97) | 4 | 0.77(0.21-1.96) | 2 | 0.54(0.07-1.96) | 2 | 1.31(0.16-4.72) | 11 | 0.92(0.46-1.65) |  |  |  |  |
| Chronic liver disease and cirrhosis | 16 | 1.82#(1.04-2.96) | 37 | 1.21(0.85-1.67) | 27 | 1.31(0.86-1.91) | 13 | 1.61(0.86-2.75) | 93 | 1.37#(1.1-1.67) |  |  |  |  |
| Nephritis, Nephrotic Syndrome and Nephrosis | 33 | 1.66#(1.14-2.33) | 78 | 1.01(0.8-1.26) | 83 | 1.31#(1.05-1.63) | 32 | 1.14(0.78-1.62) | 226 | 1.20#(1.05-1.37) |  |  |  |  |
| Complications of pregnancy, childbirth, puerperium | 0 | 0(0-145.94) | 0 | 0(0-56.27) | 0 | 0(0-149.78) | 0 | 0(0-573.93) | 0 | 0(0-30.26) |  |  |  |  |
| Congenital anomalies | 1 | 1.19(0.03-6.61) | 4 | 1.37(0.37-3.51) | 2 | 1.01(0.12-3.67) | 1 | 1.26(0.03-7.05) | 8 | 1.23(0.53-2.42) |  |  |  |  |
| Certain conditions originating in perinatal period | 0 | 0(0-1108.78) | 0 | 0(0-332.83) | 0 | 0(0-511.92) | 0 | 0(0-1325.73) | 0 | 0(0-151.19) |  |  |  |  |
| Symptoms, Signs and Ill-Defined Conditions | 16 | 1.4(0.8-2.28) | 41 | 0.89(0.64-1.2) | 37 | 0.9(0.64-1.25) | 16 | 0.89(0.51-1.45) | 110 | 0.94(0.78-1.14) |  |  |  |  |
| Accidents and Adverse Effects | 26 | 1(0.65-1.46) | 78 | 0.80#(0.63-1) | 71 | 0.92(0.72-1.16) | 46 | 1.32(0.96-1.76) | 221 | 0.94(0.82-1.07) |  |  |  |  |
| Suicide and Self-Inflicted Injury | 8 | 1.37(0.59-2.7) | 23 | 1.14(0.72-1.71) | 21 | 1.55(0.96-2.38) | 5 | 0.93(0.3-2.18) | 57 | 1.27(0.96-1.65) |  |  |  |  |
| Homicide and legal intervention | 1 | 0.82(0.02-4.6) | 5 | 1.34(0.43-3.13) | 2 | 0.99(0.12-3.58) | 0 | 0(0-5.48) | 8 | 1.05(0.45-2.06) |  |  |  |  |
| Other Cause of Death | 192 | 1.37#(1.18-1.57) | 473 | 0.84#(0.76-0.91) | 494 | 0.98(0.9-1.07) | 278 | 1.15#(1.02-1.3) | 1437 | 0.99(0.94-1.04) |  |  |  |  |

# P value less than .05

Supplementary Table 14. Standardized-mortality ratios (SMRs) for each cause of death following CRC diagnosis in patients in grade III.

| Timing of Deaths After Diagnosis | | | | | | | | | | |  |  |  |  |
| --- | --- | --- | --- | --- | --- | --- | --- | --- | --- | --- | --- | --- | --- | --- |
| Cause of death | <1years | | 1-5years | | 5-10years | | >10years | | Total | |  |  |  |  |
| Selected Events | No. Observed | SMR(95%CI) | No. Observed | SMR(95%CI) | No. Observed | SMR(95%CI) | No. Observed | SMR(95%CI) | No. Observed | SMR(95%CI) |  |  |  |  |
| All cause of death | 14558 | 8.79#(8.65-8.94) | 20093 | 3.92#(3.87-3.97) | 5764 | 1.61#(1.57-1.65) | 1942 | 1.32#(1.26-1.38) | 42357 | 3.58#(3.55-3.62) |  |  |  |  |
| All malignant cancers | 12460 | 35.74#(35.12-36.38) | 16272 | 15.68#(15.44-15.92) | 2624 | 3.86#(3.71-4.01) | 537 | 2.01#(1.84-2.19) | 31893 | 13.66#(13.51-13.81) |  |  |  |  |
| Colon and Rectum | 11570 | 333.37#(327.32-339.5) | 14852 | 145.29#(142.96-147.64) | 1927 | 29.64#(28.33-31) | 234 | 9.46#(8.28-10.75) | 28583 | 126.09#(124.63-127.56) |  |  |  |  |
| In situ, benign, or unknown behavior neoplasms | 32 | 3.13#(2.14-4.42) | 54 | 1.68#(1.26-2.19) | 26 | 1.13(0.74-1.66) | 11 | 1.15(0.57-2.06) | 123 | 1.64#(1.37-1.96) |  |  |  |  |
| Tuberculosis | 1 | 2.43(0.06-13.53) | 0 | 0(0-3.18) | 0 | 0(0-5.24) | 2 | 7.66(0.93-27.68) | 3 | 1.18(0.24-3.46) |  |  |  |  |
| syphilis | 0 | 0(0-167.43) | 0 | 0(0-57.46) | 0 | 0(0-85.79) | 0 | 0(0-199.6) | 0 | 0(0-24.97) |  |  |  |  |
| Septicemia | 92 | 3.88#(3.13-4.76) | 109 | 1.49#(1.22-1.8) | 47 | 0.93(0.68-1.23) | 26 | 1.25(0.81-1.83) | 274 | 1.63#(1.44-1.83) |  |  |  |  |
| Other infectious diseases | 40 | 3.24#(2.32-4.41) | 39 | 1.02(0.73-1.4) | 26 | 0.98(0.64-1.44) | 10 | 0.98(0.47-1.8) | 115 | 1.32#(1.09-1.59) |  |  |  |  |
| Diabetes Mellitus | 77 | 1.62#(1.28-2.03) | 154 | 1.08(0.92-1.27) | 107 | 1.14(0.93-1.37) | 53 | 1.41#(1.06-1.85) | 391 | 1.22#(1.1-1.34) |  |  |  |  |
| Alzheimers | 35 | 0.51#(0.35-0.71) | 153 | 0.65#(0.55-0.76) | 208 | 1.09(0.95-1.25) | 125 | 1.43#(1.19-1.7) | 521 | 0.89#(0.82-0.97) |  |  |  |  |
| Diseasis of hearts | 805 | 1.69#(1.58-1.81) | 1384 | 0.95(0.9-1) | 1087 | 1.11#(1.04-1.18) | 458 | 1.16#(1.06-1.27) | 3734 | 1.13#(1.09-1.17) |  |  |  |  |
| Hypertension without heart disease | 28 | 1.46(0.97-2.12) | 58 | 0.93(0.7-1.2) | 61 | 1.27(0.97-1.63) | 23 | 1.11(0.7-1.66) | 170 | 1.13(0.97-1.31) |  |  |  |  |
| Cerebrovascular diseases | 174 | 1.54#(1.32-1.78) | 284 | 0.83#(0.73-0.93) | 234 | 1.02(0.89-1.16) | 97 | 1.04(0.84-1.27) | 789 | 1.01(0.94-1.09) |  |  |  |  |
| Atherosclerosis | 11 | 1.31(0.66-2.35) | 19 | 0.78(0.47-1.22) | 17 | 1.19(0.69-1.9) | 13 | 2.55#(1.36-4.37) | 60 | 1.15(0.88-1.48) |  |  |  |  |
| Aortic aneurysm and dissection | 10 | 1.13(0.54-2.08) | 28 | 1.1(0.73-1.59) | 23 | 1.51(0.96-2.27) | 6 | 1.1(0.4-2.38) | 67 | 1.22(0.95-1.55) |  |  |  |  |
| Other diseases of arteries, arterioles, capillaries | 12 | 1.7(0.88-2.96) | 25 | 1.16(0.75-1.71) | 16 | 1.09(0.62-1.77) | 9 | 1.52(0.7-2.89) | 62 | 1.26(0.96-1.61) |  |  |  |  |
| Pneumonia and influenza | 72 | 1.56#(1.22-1.96) | 139 | 0.98(0.82-1.15) | 106 | 1.11(0.91-1.34) | 48 | 1.27(0.94-1.69) | 365 | 1.13#(1.02-1.26) |  |  |  |  |
| Chronic obstructive pulmonary disease and allied cond | 131 | 1.33#(1.12-1.58) | 313 | 1.02(0.91-1.14) | 232 | 1.08(0.95-1.23) | 107 | 1.21(0.99-1.47) | 783 | 1.11#(1.03-1.19) |  |  |  |  |
| Stomach and duodenal ulcers | 6 | 2.41(0.89-5.25) | 5 | 0.69(0.23-1.62) | 6 | 1.34(0.49-2.92) | 2 | 1.16(0.14-4.19) | 19 | 1.2(0.72-1.87) |  |  |  |  |
| Chronic liver disease and cirrhosis | 32 | 2.59#(1.77-3.66) | 29 | 0.82(0.55-1.18) | 30 | 1.38(0.93-1.97) | 7 | 0.83(0.33-1.7) | 98 | 1.26#(1.02-1.54) |  |  |  |  |
| Nephritis, Nephrotic Syndrome and Nephrosis | 53 | 1.62#(1.21-2.12) | 99 | 0.96(0.78-1.16) | 88 | 1.19(0.95-1.46) | 38 | 1.25(0.89-1.72) | 278 | 1.15#(1.02-1.3) |  |  |  |  |
| Complications of pregnancy, childbirth, puerperium | 4 | 155.94#(42.49-399.26) | 3 | 47.18#(9.73-137.88) | 1 | 35.17(0.89-195.93) | 0 | 0(0-446.47) | 8 | 63.52#(27.43-125.17) |  |  |  |  |
| Congenital anomalies | 4 | 3.13(0.85-8.01) | 1 | 0.27(0.01-1.53) | 2 | 0.89(0.11-3.22) | 1 | 1.15(0.03-6.39) | 8 | 1(0.43-1.96) |  |  |  |  |
| Certain conditions originating in perinatal period | 0 | 0(0-815.09) | 0 | 0(0-294.4) | 0 | 0(0-495.99) | 0 | 0(0-1301.84) | 0 | 0(0-134.99) |  |  |  |  |
| Symptoms, Signs and Ill-Defined Conditions | 27 | 1.3(0.86-1.9) | 72 | 1.04(0.81-1.31) | 56 | 1.04(0.79-1.36) | 22 | 1.03(0.65-1.56) | 177 | 1.07(0.92-1.24) |  |  |  |  |
| Accidents and Adverse Effects | 61 | 1.47#(1.12-1.89) | 104 | 0.80#(0.66-0.98) | 88 | 0.95(0.76-1.17) | 42 | 1.07(0.77-1.45) | 295 | 0.98(0.87-1.09) |  |  |  |  |
| Suicide and Self-Inflicted Injury | 12 | 1.5(0.78-2.62) | 38 | 1.68#(1.19-2.3) | 15 | 1.08(0.6-1.78) | 6 | 1.12(0.41-2.43) | 71 | 1.42#(1.11-1.79) |  |  |  |  |
| Homicide and legal intervention | 3 | 1.83(0.38-5.34) | 1 | 0.24(0.01-1.34) | 1 | 0.46(0.01-2.59) | 0 | 0(0-4.99) | 5 | 0.58(0.19-1.34) |  |  |  |  |
| Other Cause of Death | 376 | 1.53#(1.38-1.69) | 710 | 0.87#(0.81-0.94) | 663 | 1.05(0.97-1.13) | 299 | 1.08(0.96-1.21) | 2048 | 1.04(1-1.09) |  |  |  |  |

# P value less than .05

Supplementary Table 15. Standardized-mortality ratios (SMRs) for each cause of death following CRC diagnosis in patients in grade IV.

| Timing of Deaths After Diagnosis | | | | | | | | | | |  |  |  |  |
| --- | --- | --- | --- | --- | --- | --- | --- | --- | --- | --- | --- | --- | --- | --- |
| Cause of death | <1years | | 1-5years | | 5-10years | | >10years | | Total | |  |  |  |  |
| Selected Events | No. Observed | SMR(95%CI) | No. Observed | SMR(95%CI) | No. Observed | SMR(95%CI) | No. Observed | SMR(95%CI) | No. Observed | SMR(95%CI) |  |  |  |  |
| All cause of death | 1867 | 11.15#(10.65-11.67) | 1907 | 4.30#(4.11-4.5) | 383 | 1.73#(1.56-1.91) | 67 | 1.08(0.84-1.37) | 4224 | 4.72#(4.58-4.87) |  |  |  |  |
| All malignant cancers | 1599 | 45.75#(43.53-48.04) | 1555 | 17.34#(16.49-18.23) | 147 | 3.54#(2.99-4.16) | 19 | 1.69#(1.02-2.64) | 3320 | 18.72#(18.08-19.36) |  |  |  |  |
| Colon and Rectum | 1446 | 435.59#(413.43-458.63) | 1407 | 165.81#(157.26-174.71) | 98 | 24.97#(20.27-30.43) | 11 | 10.57#(5.28-18.92) | 2962 | 176.62#(170.32-183.1) |  |  |  |  |
| In situ, benign, or unknown behavior neoplasms | 15 | 14.33#(8.02-23.64) | 3 | 1.07(0.22-3.14) | 4 | 2.84(0.78-7.28) | 0 | 0(0-9.3) | 22 | 3.90#(2.44-5.9) |  |  |  |  |
| Tuberculosis | 0 | 0(0-107.72) | 0 | 0(0-44.09) | 0 | 0(0-101.57) | 0 | 0(0-437.09) | 0 | 0(0-22.68) |  |  |  |  |
| syphilis | 0 | 0(0-1724.21) | 0 | 0(0-667.1) | 0 | 0(0-1359.08) | 0 | 0(0-4433.6) | 0 | 0(0-328.91) |  |  |  |  |
| Septicemia | 11 | 4.54#(2.27-8.12) | 7 | 1.09(0.44-2.25) | 4 | 1.26(0.34-3.22) | 0 | 0(0-4.09) | 22 | 1.70#(1.07-2.58) |  |  |  |  |
| Other infectious diseases | 5 | 3.73#(1.21-8.7) | 6 | 1.74(0.64-3.79) | 3 | 1.85(0.38-5.41) | 1 | 2.35(0.06-13.09) | 15 | 2.19#(1.23-3.62) |  |  |  |  |
| Diabetes Mellitus | 9 | 1.92(0.88-3.64) | 12 | 0.99(0.51-1.73) | 5 | 0.87(0.28-2.02) | 1 | 0.63(0.02-3.54) | 27 | 1.12(0.74-1.62) |  |  |  |  |
| Alzheimers | 6 | 0.77(0.28-1.67) | 16 | 0.72(0.41-1.17) | 23 | 1.83#(1.16-2.75) | 8 | 2.07(0.89-4.07) | 53 | 1.14(0.86-1.5) |  |  |  |  |
| Diseasis of hearts | 81 | 1.79#(1.42-2.22) | 128 | 1.07(0.89-1.27) | 86 | 1.45#(1.16-1.79) | 16 | 0.97(0.55-1.57) | 311 | 1.29#(1.15-1.44) |  |  |  |  |
| Hypertension without heart disease | 5 | 2.4(0.78-5.6) | 4 | 0.7(0.19-1.8) | 1 | 0.33(0.01-1.82) | 0 | 0(0-4.14) | 10 | 0.85(0.41-1.57) |  |  |  |  |
| Cerebrovascular diseases | 24 | 2.24#(1.44-3.33) | 31 | 1.09(0.74-1.55) | 9 | 0.63(0.29-1.2) | 2 | 0.5(0.06-1.81) | 66 | 1.15(0.89-1.47) |  |  |  |  |
| Atherosclerosis | 1 | 1.47(0.04-8.17) | 0 | 0(0-2.09) | 1 | 1.2(0.03-6.7) | 0 | 0(0-17.35) | 2 | 0.57(0.07-2.07) |  |  |  |  |
| Aortic aneurysm and dissection | 1 | 1.3(0.03-7.26) | 1 | 0.52(0.01-2.87) | 0 | 0(0-4.22) | 2 | 8.96#(1.09-32.37) | 4 | 1.05(0.29-2.69) |  |  |  |  |
| Other diseases of arteries, arterioles, capillaries | 2 | 2.92(0.35-10.54) | 3 | 1.65(0.34-4.83) | 3 | 3.3(0.68-9.66) | 0 | 0(0-14.45) | 8 | 2.18(0.94-4.3) |  |  |  |  |
| Pneumonia and influenza | 7 | 1.63(0.66-3.37) | 10 | 0.88(0.42-1.62) | 5 | 0.88(0.29-2.06) | 1 | 0.65(0.02-3.62) | 23 | 1.01(0.64-1.51) |  |  |  |  |
| Chronic obstructive pulmonary disease and allied cond | 20 | 1.96#(1.2-3.03) | 26 | 0.96(0.63-1.41) | 20 | 1.5(0.91-2.31) | 4 | 1.07(0.29-2.73) | 70 | 1.29#(1-1.63) |  |  |  |  |
| Stomach and duodenal ulcers | 0 | 0(0-16.6) | 0 | 0(0-6.49) | 0 | 0(0-13.85) | 0 | 0(0-52.1) | 0 | 0(0-3.27) |  |  |  |  |
| Chronic liver disease and cirrhosis | 3 | 2.24(0.46-6.54) | 3 | 0.92(0.19-2.68) | 2 | 1.47(0.18-5.31) | 0 | 0(0-10.43) | 8 | 1.26(0.55-2.49) |  |  |  |  |
| Nephritis, Nephrotic Syndrome and Nephrosis | 6 | 1.8(0.66-3.92) | 11 | 1.23(0.62-2.21) | 9 | 1.98(0.91-3.77) | 2 | 1.57(0.19-5.66) | 28 | 1.55#(1.03-2.24) |  |  |  |  |
| Complications of pregnancy, childbirth, puerperium | 0 | 0(0-1024.03) | 0 | 0(0-482.64) | 0 | 0(0-1343.43) | 0 | 0(0-10589.23) | 0 | 0(0-257.25) |  |  |  |  |
| Congenital anomalies | 0 | 0(0-28.02) | 0 | 0(0-11.38) | 0 | 0(0-25.85) | 0 | 0(0-97.6) | 0 | 0(0-5.8) |  |  |  |  |
| Certain conditions originating in perinatal period | 0 | 0(0-7558.53) | 0 | 0(0-3162.47) | 0 | 0(0-8116.26) | 0 | 0(0-31364.38) | 0 | 0(0-1656.72) |  |  |  |  |
| Symptoms, Signs and Ill-Defined Conditions | 10 | 4.45#(2.13-8.18) | 7 | 1.14(0.46-2.34) | 6 | 1.81(0.66-3.94) | 1 | 1.12(0.03-6.24) | 24 | 1.90#(1.22-2.83) |  |  |  |  |
| Accidents and Adverse Effects | 9 | 1.97(0.9-3.74) | 17 | 1.43(0.83-2.29) | 7 | 1.21(0.48-2.48) | 0 | 0(0-2.26) | 33 | 1.38(0.95-1.94) |  |  |  |  |
| Suicide and Self-Inflicted Injury | 3 | 3.45(0.71-10.07) | 0 | 0(0-1.77) | 1 | 1.2(0.03-6.71) | 1 | 4.81(0.12-26.82) | 5 | 1.25(0.41-2.92) |  |  |  |  |
| Homicide and legal intervention | 0 | 0(0-20.98) | 0 | 0(0-9.71) | 0 | 0(0-26.46) | 0 | 0(0-120.72) | 0 | 0(0-5.08) |  |  |  |  |
| Other Cause of Death | 50 | 1.82#(1.35-2.4) | 67 | 0.89(0.69-1.13) | 47 | 1.16(0.86-1.55) | 9 | 0.76(0.35-1.45) | 173 | 1.12(0.96-1.3) |  |  |  |  |

# P value less than .05

Supplementary Table 16. Standardized-mortality ratios (SMRs) for each cause of death following CRC diagnosis in patients with chemotherapy.

| Timing of Deaths After Diagnosis | | | | | | | | | | |  |  |  |  |
| --- | --- | --- | --- | --- | --- | --- | --- | --- | --- | --- | --- | --- | --- | --- |
| Cause of death | <1years | | 1-5years | | 5-10years | | >10years | | Total | |  |  |  |  |
| Selected Events | No. Observed | SMR(95%CI) | No. Observed | SMR(95%CI) | No. Observed | SMR(95%CI) | No. Observed | SMR(95%CI) | No. Observed | SMR(95%CI) |  |  |  |  |
| All cause of death | 21405 | 8.40#(8.29-8.51) | 52768 | 6.47#(6.42-6.53) | 13439 | 2.23#(2.19-2.26) | 3833 | 1.30#(1.26-1.35) | 91445 | 4.65#(4.62-4.68) |  |  |  |  |
| All malignant cancers | 19086 | 27.82#(27.43-28.22) | 47654 | 22.37#(22.17-22.57) | 9166 | 6.43#(6.3-6.56) | 1529 | 2.47#(2.34-2.59) | 77435 | 15.93#(15.81-16.04) |  |  |  |  |
| Colon and Rectum | 17789 | 284.97#(280.8-289.19) | 44525 | 231.90#(229.75-234.06) | 7545 | 59.60#(58.26-60.96) | 823 | 15.11#(14.1-16.18) | 70682 | 162.30#(161.11-163.51) |  |  |  |  |
| In situ, benign, or unknown behavior neoplasms | 40 | 2.57#(1.83-3.5) | 92 | 1.80#(1.45-2.21) | 61 | 1.54#(1.18-1.98) | 17 | 0.86(0.5-1.38) | 210 | 1.67#(1.45-1.91) |  |  |  |  |
| Tuberculosis | 2 | 2.51(0.3-9.07) | 2 | 0.84(0.1-3.04) | 2 | 1.36(0.16-4.89) | 2 | 3.35(0.41-12.11) | 8 | 1.52(0.66-3) |  |  |  |  |
| syphilis | 0 | 0(0-84.27) | 0 | 0(0-27.83) | 0 | 0(0-40.47) | 0 | 0(0-88.6) | 0 | 0(0-11.93) |  |  |  |  |
| Septicemia | 140 | 3.68#(3.1-4.35) | 222 | 1.82#(1.59-2.08) | 102 | 1.13(0.92-1.37) | 43 | 0.99(0.72-1.33) | 507 | 1.73#(1.58-1.88) |  |  |  |  |
| Other infectious diseases | 66 | 2.47#(1.91-3.14) | 104 | 1.29#(1.05-1.56) | 55 | 1.04(0.79-1.36) | 23 | 1.03(0.65-1.54) | 248 | 1.36#(1.2-1.54) |  |  |  |  |
| Diabetes Mellitus | 53 | 0.62#(0.46-0.81) | 203 | 0.76#(0.66-0.87) | 215 | 1.17#(1.02-1.34) | 115 | 1.39#(1.14-1.66) | 586 | 0.95(0.87-1.03) |  |  |  |  |
| Alzheimers | 8 | 0.13#(0.05-0.25) | 71 | 0.31#(0.24-0.39) | 175 | 0.75#(0.65-0.87) | 171 | 1.18#(1.01-1.37) | 425 | 0.63#(0.57-0.7) |  |  |  |  |
| Diseasis of hearts | 842 | 1.27#(1.18-1.36) | 1799 | 0.85#(0.81-0.89) | 1410 | 0.90#(0.86-0.95) | 744 | 0.97(0.91-1.05) | 4795 | 0.94#(0.91-0.97) |  |  |  |  |
| Hypertension without heart disease | 22 | 0.87(0.54-1.31) | 68 | 0.8(0.62-1.01) | 64 | 0.89(0.69-1.14) | 44 | 1.14(0.83-1.53) | 198 | 0.9(0.78-1.03) |  |  |  |  |
| Cerebrovascular diseases | 164 | 1.16(0.99-1.35) | 334 | 0.73#(0.66-0.82) | 292 | 0.85#(0.75-0.95) | 155 | 0.89(0.76-1.04) | 945 | 0.85#(0.79-0.9) |  |  |  |  |
| Atherosclerosis | 10 | 1.21(0.58-2.22) | 20 | 0.77(0.47-1.18) | 16 | 0.84(0.48-1.37) | 14 | 1.57(0.86-2.64) | 60 | 0.96(0.74-1.24) |  |  |  |  |
| Aortic aneurysm and dissection | 14 | 0.97(0.53-1.63) | 21 | 0.48#(0.3-0.73) | 16 | 0.57#(0.33-0.93) | 16 | 1.37(0.78-2.23) | 67 | 0.68#(0.53-0.87) |  |  |  |  |
| Other diseases of arteries, arterioles, capillaries | 17 | 1.73#(1.01-2.77) | 33 | 1.05(0.72-1.47) | 19 | 0.81(0.49-1.26) | 13 | 1.13(0.6-1.93) | 82 | 1.07(0.85-1.33) |  |  |  |  |
| Pneumonia and influenza | 64 | 1.17(0.9-1.49) | 134 | 0.74#(0.62-0.88) | 132 | 0.93(0.78-1.1) | 57 | 0.8(0.61-1.04) | 387 | 0.86#(0.78-0.95) |  |  |  |  |
| Chronic obstructive pulmonary disease and allied cond | 130 | 0.83#(0.69-0.98) | 370 | 0.72#(0.65-0.8) | 360 | 0.93(0.84-1.04) | 140 | 0.75#(0.63-0.89) | 1000 | 0.81#(0.76-0.86) |  |  |  |  |
| Stomach and duodenal ulcers | 11 | 3.03#(1.51-5.41) | 10 | 0.89(0.43-1.64) | 13 | 1.71(0.91-2.93) | 3 | 0.86(0.18-2.52) | 37 | 1.43#(1.01-1.97) |  |  |  |  |
| Chronic liver disease and cirrhosis | 31 | 0.91(0.62-1.29) | 72 | 0.72#(0.56-0.9) | 61 | 1.04(0.79-1.33) | 21 | 0.92(0.57-1.4) | 185 | 0.86#(0.74-0.99) |  |  |  |  |
| Nephritis, Nephrotic Syndrome and Nephrosis | 42 | 0.85(0.62-1.15) | 131 | 0.81#(0.68-0.96) | 126 | 1.01(0.84-1.2) | 61 | 0.99(0.76-1.27) | 360 | 0.91(0.81-1) |  |  |  |  |
| Complications of pregnancy, childbirth, puerperium | 4 | 37.34#(10.17-95.59) | 11 | 41.07#(20.5-73.48) | 2 | 19.25#(2.33-69.52) | 0 | 0(0-142.29) | 17 | 33.67#(19.62-53.91) |  |  |  |  |
| Congenital anomalies | 2 | 0.69(0.08-2.49) | 2 | 0.24#(0.03-0.85) | 3 | 0.6(0.12-1.75) | 1 | 0.49(0.01-2.72) | 8 | 0.43#(0.19-0.86) |  |  |  |  |
| Certain conditions originating in perinatal period | 0 | 0(0-276.54) | 0 | 0(0-96.12) | 0 | 0(0-182.08) | 0 | 0(0-498.14) | 0 | 0(0-46.47) |  |  |  |  |
| Symptoms, Signs and Ill-Defined Conditions | 46 | 1.85#(1.35-2.46) | 128 | 1.53#(1.28-1.82) | 85 | 1.18(0.94-1.46) | 33 | 0.89(0.61-1.25) | 292 | 1.34#(1.19-1.5) |  |  |  |  |
| Accidents and Adverse Effects | 107 | 1.35#(1.11-1.63) | 191 | 0.78#(0.67-0.9) | 169 | 1(0.85-1.16) | 67 | 0.83(0.64-1.05) | 534 | 0.93(0.85-1.01) |  |  |  |  |
| Suicide and Self-Inflicted Injury | 40 | 1.71#(1.22-2.32) | 102 | 1.50#(1.22-1.82) | 51 | 1.32(0.98-1.73) | 19 | 1.29(0.78-2.01) | 212 | 1.46#(1.27-1.67) |  |  |  |  |
| Homicide and legal intervention | 3 | 0.57(0.12-1.67) | 12 | 0.88(0.46-1.54) | 7 | 1.11(0.45-2.29) | 4 | 1.95(0.53-4.99) | 26 | 0.96(0.62-1.4) |  |  |  |  |
| Other Cause of Death | 461 | 1.38#(1.26-1.51) | 982 | 0.87#(0.82-0.93) | 837 | 0.88#(0.82-0.94) | 541 | 1.05(0.96-1.14) | 2821 | 0.96#(0.93-1) |  |  |  |  |

# P value less than .05

Supplementary Table 17. Standardized-mortality ratios (SMRs) for each cause of death following CRC diagnosis in patients with radiation.

| Timing of Deaths After Diagnosis | | | | | | | | | | |  |  |  |  |
| --- | --- | --- | --- | --- | --- | --- | --- | --- | --- | --- | --- | --- | --- | --- |
| Cause of death | <1years | | 1-5years | | 5-10years | | >10years | | Total | |  |  |  |  |
| Selected Events | No. Observed | SMR(95%CI) | No. Observed | SMR(95%CI) | No. Observed | SMR(95%CI) | No. Observed | SMR(95%CI) | No. Observed | SMR(95%CI) |  |  |  |  |
| All cause of death | 6835 | 6.59#(6.43-6.75) | 17230 | 5.11#(5.04-5.19) | 5793 | 2.42#(2.35-2.48) | 1667 | 1.52#(1.45-1.59) | 31525 | 3.99#(3.94-4.03) |  |  |  |  |
| All malignant cancers | 5743 | 21.70#(21.14-22.27) | 14699 | 17.10#(16.83-17.38) | 3874 | 6.75#(6.54-6.97) | 698 | 2.87#(2.66-3.09) | 25014 | 12.89#(12.73-13.05) |  |  |  |  |
| Colon and Rectum | 5283 | 215.74#(209.96-221.64) | 13483 | 172.93#(170.02-175.87) | 3132 | 61.84#(59.69-64.05) | 378 | 18.04#(16.26-19.95) | 22276 | 127.98#(126.31-129.67) |  |  |  |  |
| In situ, benign, or unknown behavior neoplasms | 11 | 1.73(0.86-3.09) | 44 | 2.07#(1.51-2.78) | 27 | 1.71#(1.12-2.48) | 7 | 0.94(0.38-1.93) | 89 | 1.75#(1.4-2.15) |  |  |  |  |
| Tuberculosis | 2 | 6.33(0.77-22.87) | 0 | 0(0-3.81) | 1 | 1.7(0.04-9.5) | 1 | 4.35(0.11-24.23) | 4 | 1.9(0.52-4.88) |  |  |  |  |
| syphilis | 0 | 0(0-218.18) | 0 | 0(0-69.94) | 0 | 0(0-104.3) | 0 | 0(0-238.12) | 0 | 0(0-30.61) |  |  |  |  |
| Septicemia | 55 | 3.65#(2.75-4.75) | 93 | 1.89#(1.52-2.31) | 53 | 1.51#(1.13-1.97) | 24 | 1.49(0.95-2.21) | 225 | 1.95#(1.7-2.22) |  |  |  |  |
| Other infectious diseases | 32 | 3.09#(2.12-4.37) | 48 | 1.46#(1.08-1.94) | 29 | 1.36(0.91-1.95) | 8 | 0.93(0.4-1.82) | 117 | 1.60#(1.32-1.92) |  |  |  |  |
| Diabetes Mellitus | 28 | 0.84(0.56-1.21) | 110 | 1.02(0.84-1.23) | 89 | 1.22(0.98-1.5) | 50 | 1.58#(1.17-2.08) | 277 | 1.13(1-1.27) |  |  |  |  |
| Alzheimers | 7 | 0.24#(0.1-0.5) | 46 | 0.46#(0.34-0.62) | 69 | 0.79#(0.61-0.99) | 57 | 1.15(0.87-1.5) | 179 | 0.68#(0.58-0.78) |  |  |  |  |
| Diseasis of hearts | 414 | 1.49#(1.35-1.64) | 912 | 1.03(0.96-1.09) | 639 | 1.03(0.95-1.11) | 307 | 1.08(0.96-1.21) | 2272 | 1.09#(1.05-1.14) |  |  |  |  |
| Hypertension without heart disease | 10 | 0.95(0.46-1.75) | 32 | 0.91(0.62-1.29) | 33 | 1.2(0.83-1.69) | 24 | 1.76#(1.13-2.62) | 99 | 1.14(0.93-1.39) |  |  |  |  |
| Cerebrovascular diseases | 68 | 1.15(0.89-1.45) | 149 | 0.79#(0.67-0.93) | 109 | 0.82#(0.67-0.99) | 67 | 1.08(0.84-1.37) | 393 | 0.89#(0.8-0.98) |  |  |  |  |
| Atherosclerosis | 9 | 2.40#(1.1-4.56) | 8 | 0.71(0.31-1.4) | 6 | 0.81(0.3-1.76) | 6 | 1.89(0.69-4.12) | 29 | 1.13(0.76-1.63) |  |  |  |  |
| Aortic aneurysm and dissection | 8 | 1.36(0.59-2.68) | 8 | 0.44#(0.19-0.86) | 9 | 0.8(0.36-1.51) | 9 | 2(0.91-3.79) | 34 | 0.85(0.59-1.19) |  |  |  |  |
| Other diseases of arteries, arterioles, capillaries | 10 | 2.47#(1.19-4.54) | 17 | 1.31(0.76-2.1) | 11 | 1.19(0.6-2.14) | 6 | 1.42(0.52-3.09) | 44 | 1.44#(1.05-1.94) |  |  |  |  |
| Pneumonia and influenza | 36 | 1.51#(1.05-2.09) | 68 | 0.88(0.69-1.12) | 63 | 1.13(0.87-1.44) | 22 | 0.85(0.53-1.29) | 189 | 1.04(0.89-1.19) |  |  |  |  |
| Chronic obstructive pulmonary disease and allied cond | 70 | 1.12(0.87-1.42) | 200 | 0.96(0.83-1.1) | 172 | 1.13(0.97-1.31) | 65 | 0.92(0.71-1.18) | 507 | 1.03(0.94-1.12) |  |  |  |  |
| Stomach and duodenal ulcers | 5 | 3.34#(1.08-7.79) | 5 | 1.08(0.35-2.51) | 3 | 0.99(0.2-2.9) | 1 | 0.76(0.02-4.25) | 14 | 1.34(0.73-2.24) |  |  |  |  |
| Chronic liver disease and cirrhosis | 24 | 1.82#(1.17-2.71) | 31 | 0.74(0.5-1.05) | 16 | 0.63(0.36-1.03) | 7 | 0.72(0.29-1.48) | 78 | 0.87(0.69-1.08) |  |  |  |  |
| Nephritis, Nephrotic Syndrome and Nephrosis | 26 | 1.3(0.85-1.91) | 61 | 0.92(0.71-1.19) | 53 | 1.08(0.81-1.41) | 27 | 1.19(0.78-1.73) | 167 | 1.06(0.9-1.23) |  |  |  |  |
| Complications of pregnancy, childbirth, puerperium | 1 | 29.82(0.75-166.13) | 0 | 0(0-40.53) | 1 | 27.07(0.69-150.84) | 0 | 0(0-426.21) | 2 | 11.75#(1.42-42.46) |  |  |  |  |
| Congenital anomalies | 0 | 0(0-3.28) | 0 | 0(0-1.06) | 0 | 0(0-1.78) | 0 | 0(0-4.52) | 0 | 0.00#(0-0.49) |  |  |  |  |
| Certain conditions originating in perinatal period | 0 | 0(0-738.19) | 0 | 0(0-243.77) | 0 | 0(0-433.52) | 0 | 0(0-1220.93) | 0 | 0(0-116.51) |  |  |  |  |
| Symptoms, Signs and Ill-Defined Conditions | 17 | 1.57(0.92-2.52) | 72 | 2.02#(1.58-2.54) | 36 | 1.28(0.9-1.77) | 17 | 1.29(0.75-2.07) | 142 | 1.62#(1.36-1.91) |  |  |  |  |
| Accidents and Adverse Effects | 35 | 1.09(0.76-1.51) | 87 | 0.84(0.68-1.04) | 77 | 1.1(0.87-1.38) | 32 | 1.03(0.7-1.45) | 231 | 0.98(0.86-1.11) |  |  |  |  |
| Suicide and Self-Inflicted Injury | 18 | 1.90#(1.13-3) | 52 | 1.76#(1.31-2.31) | 25 | 1.44(0.93-2.12) | 13 | 1.99#(1.06-3.4) | 108 | 1.72#(1.41-2.07) |  |  |  |  |
| Homicide and legal intervention | 0 | 0(0-1.86) | 7 | 1.27(0.51-2.63) | 5 | 1.9(0.62-4.42) | 2 | 2.37(0.29-8.57) | 14 | 1.28(0.7-2.14) |  |  |  |  |
| Other Cause of Death | 206 | 1.48#(1.28-1.69) | 481 | 1.02(0.93-1.12) | 393 | 1.05(0.95-1.16) | 217 | 1.16#(1.01-1.32) | 1297 | 1.11#(1.05-1.17) |  |  |  |  |

# P value less than .05

Supplementary Table 18. Standardized-mortality ratios (SMRs) for each cause of death following CRC diagnosis in patients with surgery.

| Timing of Deaths After Diagnosis | | | | | | | | | | |  |  |  |  |
| --- | --- | --- | --- | --- | --- | --- | --- | --- | --- | --- | --- | --- | --- | --- |
| Cause of death | <1years | | 1-5years | | 5-10years | | >10years | | Total | |  |  |  |  |
| Selected Events | No. Observed | SMR(95%CI) | No. Observed | SMR(95%CI) | No. Observed | SMR(95%CI) | No. Observed | SMR(95%CI) | No. Observed | SMR(95%CI) |  |  |  |  |
| All cause of death | 36380 | 3.79#(3.75-3.83) | 90843 | 2.60#(2.58-2.61) | 40413 | 1.56#(1.54-1.57) | 14417 | 1.29#(1.27-1.31) | 182053 | 2.23#(2.22-2.24) |  |  |  |  |
| All malignant cancers | 26503 | 12.38#(12.23-12.53) | 65168 | 8.64#(8.58-8.71) | 18046 | 3.43#(3.38-3.48) | 3939 | 1.84#(1.78-1.9) | 113656 | 6.65#(6.62-6.69) |  |  |  |  |
| Colon and Rectum | 24485 | 117.31#(115.84-118.79) | 57273 | 78.94#(78.29-79.59) | 12530 | 25.51#(25.06-25.96) | 1665 | 8.58#(8.17-9) | 95953 | 59.25#(58.87-59.62) |  |  |  |  |
| In situ, benign, or unknown behavior neoplasms | 70 | 1.18(0.92-1.48) | 275 | 1.25#(1.1-1.4) | 198 | 1.17#(1.02-1.35) | 81 | 1.09(0.86-1.35) | 624 | 1.19#(1.1-1.29) |  |  |  |  |
| Tuberculosis | 5 | 1.92(0.62-4.49) | 10 | 1.15(0.55-2.11) | 5 | 0.89(0.29-2.07) | 5 | 2.33(0.76-5.45) | 25 | 1.31(0.85-1.93) |  |  |  |  |
| syphilis | 0 | 0(0-25.48) | 0 | 0(0-7.3) | 1 | 2.79(0.07-15.54) | 0 | 0(0-24.26) | 1 | 0.86(0.02-4.8) |  |  |  |  |
| Septicemia | 430 | 3.07#(2.79-3.38) | 666 | 1.31#(1.21-1.41) | 438 | 1.16#(1.06-1.28) | 175 | 1.08(0.93-1.25) | 1709 | 1.44#(1.37-1.51) |  |  |  |  |
| Other infectious diseases | 202 | 2.66#(2.31-3.06) | 277 | 1.01(0.89-1.14) | 190 | 0.96(0.83-1.1) | 71 | 0.9(0.7-1.13) | 740 | 1.18#(1.1-1.27) |  |  |  |  |
| Diabetes Mellitus | 387 | 1.34#(1.21-1.49) | 1127 | 1.11#(1.04-1.17) | 915 | 1.27#(1.19-1.36) | 424 | 1.42#(1.29-1.57) | 2853 | 1.23#(1.18-1.27) |  |  |  |  |
| Alzheimers | 149 | 0.42#(0.35-0.49) | 917 | 0.63#(0.59-0.68) | 1342 | 1.07#(1.01-1.12) | 838 | 1.35#(1.26-1.44) | 3246 | 0.88#(0.85-0.91) |  |  |  |  |
| Diseasis of hearts | 3762 | 1.38#(1.34-1.43) | 9534 | 0.97#(0.96-0.99) | 7892 | 1.12#(1.09-1.14) | 3521 | 1.18#(1.14-1.22) | 24709 | 1.10#(1.08-1.11) |  |  |  |  |
| Hypertension without heart disease | 134 | 1.24#(1.04-1.47) | 398 | 0.96(0.86-1.05) | 424 | 1.25#(1.14-1.38) | 199 | 1.29#(1.11-1.48) | 1155 | 1.13#(1.07-1.2) |  |  |  |  |
| Cerebrovascular diseases | 797 | 1.26#(1.18-1.35) | 2016 | 0.90#(0.86-0.94) | 1621 | 1.01(0.96-1.06) | 727 | 1.05(0.98-1.13) | 5161 | 1(0.97-1.02) |  |  |  |  |
| Atherosclerosis | 57 | 1.27(0.96-1.64) | 154 | 1.01(0.85-1.18) | 94 | 0.96(0.78-1.18) | 54 | 1.45#(1.09-1.9) | 359 | 1.08(0.97-1.2) |  |  |  |  |
| Aortic aneurysm and dissection | 66 | 1.26(0.97-1.6) | 151 | 0.85#(0.72-0.99) | 109 | 0.96(0.79-1.16) | 47 | 1.1(0.81-1.46) | 373 | 0.96(0.87-1.07) |  |  |  |  |
| Other diseases of arteries, arterioles, capillaries | 65 | 1.61#(1.24-2.05) | 126 | 0.87(0.72-1.03) | 125 | 1.19(0.99-1.41) | 54 | 1.21(0.91-1.57) | 370 | 1.1(0.99-1.22) |  |  |  |  |
| Pneumonia and influenza | 342 | 1.32#(1.19-1.47) | 813 | 0.87#(0.81-0.93) | 781 | 1.15#(1.07-1.24) | 329 | 1.16#(1.03-1.29) | 2265 | 1.05#(1.01-1.09) |  |  |  |  |
| Chronic obstructive pulmonary disease and allied cond | 647 | 1.13#(1.04-1.22) | 2046 | 0.97(0.93-1.02) | 1705 | 1.08#(1.03-1.14) | 708 | 1.04(0.96-1.12) | 5106 | 1.04#(1.01-1.06) |  |  |  |  |
| Stomach and duodenal ulcers | 26 | 1.82#(1.19-2.66) | 47 | 0.96(0.71-1.28) | 43 | 1.33(0.96-1.79) | 11 | 0.84(0.42-1.5) | 127 | 1.17(0.97-1.39) |  |  |  |  |
| Chronic liver disease and cirrhosis | 168 | 2.08#(1.78-2.42) | 318 | 1.17#(1.04-1.3) | 236 | 1.34#(1.17-1.52) | 88 | 1.29#(1.03-1.59) | 810 | 1.36#(1.26-1.45) |  |  |  |  |
| Nephritis, Nephrotic Syndrome and Nephrosis | 304 | 1.58#(1.4-1.76) | 751 | 1.04(0.97-1.12) | 668 | 1.21#(1.12-1.31) | 268 | 1.13#(1-1.28) | 1991 | 1.17#(1.12-1.22) |  |  |  |  |
| Complications of pregnancy, childbirth, puerperium | 7 | 39.09#(15.72-80.54) | 11 | 21.60#(10.78-38.64) | 1 | 4.46(0.11-24.86) | 1 | 16.44(0.42-91.58) | 20 | 20.55#(12.55-31.73) |  |  |  |  |
| Congenital anomalies | 12 | 1.53(0.79-2.67) | 18 | 0.68(0.4-1.08) | 15 | 0.88(0.49-1.45) | 3 | 0.44(0.09-1.3) | 48 | 0.83(0.61-1.1) |  |  |  |  |
| Certain conditions originating in perinatal period | 0 | 0(0-123.34) | 0 | 0(0-37.03) | 1 | 16.27(0.41-90.63) | 0 | 0(0-160.37) | 1 | 4.67(0.12-26.03) |  |  |  |  |
| Symptoms, Signs and Ill-Defined Conditions | 157 | 1.39#(1.18-1.63) | 426 | 0.97(0.88-1.06) | 377 | 1.04(0.94-1.15) | 145 | 0.94(0.79-1.11) | 1105 | 1.03(0.97-1.09) |  |  |  |  |
| Accidents and Adverse Effects | 251 | 1.01(0.89-1.15) | 795 | 0.88#(0.82-0.95) | 646 | 0.96(0.88-1.03) | 316 | 1.06(0.95-1.18) | 2008 | 0.95#(0.91-0.99) |  |  |  |  |
| Suicide and Self-Inflicted Injury | 70 | 1.32#(1.03-1.67) | 233 | 1.31#(1.14-1.49) | 134 | 1.17(0.98-1.38) | 48 | 1.08(0.8-1.43) | 485 | 1.24#(1.13-1.36) |  |  |  |  |
| Homicide and legal intervention | 11 | 1.02(0.51-1.83) | 35 | 1.07(0.74-1.48) | 19 | 1.07(0.65-1.67) | 8 | 1.35(0.58-2.66) | 73 | 1.09(0.85-1.37) |  |  |  |  |
| Other Cause of Death | 1758 | 1.27#(1.21-1.33) | 4531 | 0.85#(0.82-0.87) | 4387 | 0.99(0.96-1.02) | 2357 | 1.14#(1.1-1.19) | 13033 | 0.99(0.97-1) |  |  |  |  |

# P value less than .05

Supplementary Table 19. Standardized-mortality ratios (SMRs) for each cause of death following CRC diagnosis in patients without surgery.

| Timing of Deaths After Diagnosis | | | | | | | | | | |  |  |  |  |
| --- | --- | --- | --- | --- | --- | --- | --- | --- | --- | --- | --- | --- | --- | --- |
| Cause of death | <1years | | 1-5years | | 5-10years | | >10years | | Total | |  |  |  |  |
| Selected Events | No. Observed | SMR(95%CI) | No. Observed | SMR(95%CI) | No. Observed | SMR(95%CI) | No. Observed | SMR(95%CI) | No. Observed | SMR(95%CI) |  |  |  |  |
| All cause of death | 4393 | 12.81#(12.44-13.2) | 3519 | 6.09#(5.89-6.29) | 530 | 2.00#(1.83-2.18) | 185 | 1.23#(1.06-1.42) | 8627 | 6.45#(6.32-6.59) |  |  |  |  |
| All malignant cancers | 3564 | 58.23#(56.33-60.17) | 2759 | 24.49#(23.58-25.42) | 272 | 4.94#(4.37-5.56) | 55 | 1.85#(1.39-2.41) | 6650 | 25.70#(25.09-26.33) |  |  |  |  |
| Colon and Rectum | 3234 | 488.38#(471.69-505.51) | 2525 | 217.55#(209.15-226.21) | 210 | 40.12#(34.87-45.92) | 19 | 7.05#(4.24-11) | 5988 | 228.90#(223.14-234.77) |  |  |  |  |
| In situ, benign, or unknown behavior neoplasms | 17 | 8.26#(4.81-13.22) | 9 | 2.56#(1.17-4.87) | 4 | 2.37(0.64-6.06) | 0 | 0(0-3.74) | 30 | 3.64#(2.45-5.19) |  |  |  |  |
| Tuberculosis | 0 | 0(0-41.27) | 0 | 0(0-23.5) | 1 | 15.84(0.4-88.28) | 1 | 33.07(0.84-184.27) | 2 | 5.89(0.71-21.27) |  |  |  |  |
| syphilis | 0 | 0(0-601.35) | 0 | 0(0-343.33) | 0 | 0(0-833.78) | 0 | 0(0-1596.74) | 0 | 0(0-156.22) |  |  |  |  |
| Septicemia | 30 | 5.96#(4.02-8.52) | 28 | 3.25#(2.16-4.7) | 6 | 1.52(0.56-3.31) | 1 | 0.45(0.01-2.51) | 65 | 3.28#(2.53-4.18) |  |  |  |  |
| Other infectious diseases | 10 | 4.66#(2.23-8.57) | 9 | 2.20#(1-4.17) | 2 | 0.94(0.11-3.41) | 3 | 2.68(0.55-7.82) | 24 | 2.53#(1.62-3.77) |  |  |  |  |
| Diabetes Mellitus | 34 | 3.62#(2.51-5.06) | 24 | 1.44(0.93-2.15) | 9 | 1.17(0.54-2.23) | 7 | 1.69(0.68-3.48) | 74 | 1.96#(1.54-2.46) |  |  |  |  |
| Alzheimers | 23 | 1.43(0.9-2.14) | 30 | 1.17(0.79-1.68) | 17 | 1.41(0.82-2.25) | 10 | 1.26(0.6-2.32) | 80 | 1.30#(1.03-1.61) |  |  |  |  |
| Diseasis of hearts | 349 | 3.30#(2.96-3.67) | 293 | 1.71#(1.52-1.92) | 98 | 1.34#(1.09-1.64) | 51 | 1.28(0.95-1.68) | 791 | 2.03#(1.89-2.17) |  |  |  |  |
| Hypertension without heart disease | 20 | 4.48#(2.73-6.91) | 14 | 1.88#(1.03-3.15) | 3 | 0.86(0.18-2.53) | 2 | 0.96(0.12-3.48) | 39 | 2.23#(1.59-3.05) |  |  |  |  |
| Cerebrovascular diseases | 48 | 1.87#(1.38-2.48) | 58 | 1.43#(1.09-1.85) | 10 | 0.6(0.29-1.11) | 4 | 0.44(0.12-1.12) | 120 | 1.31#(1.08-1.56) |  |  |  |  |
| Atherosclerosis | 6 | 2.86#(1.05-6.22) | 11 | 3.60#(1.8-6.43) | 1 | 0.97(0.02-5.41) | 0 | 0(0-7.52) | 18 | 2.69#(1.6-4.26) |  |  |  |  |
| Aortic aneurysm and dissection | 4 | 2.32(0.63-5.93) | 3 | 1.03(0.21-3.02) | 0 | 0(0-3.05) | 1 | 1.73(0.04-9.62) | 8 | 1.25(0.54-2.45) |  |  |  |  |
| Other diseases of arteries, arterioles, capillaries | 5 | 3.15#(1.02-7.36) | 3 | 1.16(0.24-3.4) | 2 | 1.82(0.22-6.56) | 1 | 1.66(0.04-9.24) | 11 | 1.87(0.94-3.35) |  |  |  |  |
| Pneumonia and influenza | 12 | 1.08(0.56-1.89) | 23 | 1.33(0.84-2) | 6 | 0.86(0.32-1.88) | 1 | 0.27(0.01-1.48) | 42 | 1.07(0.77-1.45) |  |  |  |  |
| Chronic obstructive pulmonary disease and allied cond | 49 | 2.70#(2-3.57) | 52 | 1.64#(1.22-2.15) | 17 | 1.08(0.63-1.73) | 9 | 0.99(0.45-1.88) | 127 | 1.70#(1.42-2.02) |  |  |  |  |
| Stomach and duodenal ulcers | 2 | 3.77(0.46-13.61) | 0 | 0(0-4.34) | 0 | 0(0-10.94) | 0 | 0(0-20.86) | 2 | 1.06(0.13-3.81) |  |  |  |  |
| Chronic liver disease and cirrhosis | 8 | 4.78#(2.06-9.41) | 5 | 1.42(0.46-3.31) | 2 | 1.07(0.13-3.86) | 0 | 0(0-3.59) | 15 | 1.85#(1.04-3.06) |  |  |  |  |
| Nephritis, Nephrotic Syndrome and Nephrosis | 24 | 3.31#(2.12-4.92) | 24 | 1.94#(1.24-2.88) | 19 | 3.30#(1.98-5.15) | 4 | 1.24(0.34-3.19) | 71 | 2.48#(1.94-3.13) |  |  |  |  |
| Complications of pregnancy, childbirth, puerperium | 0 | 0(0-1718.82) | 0 | 0(0-666.62) | 0 | 0(0-1162.63) | 1 | 833.14#(21.09-4641.94) | 1 | 82.97#(2.1-462.26) |  |  |  |  |
| Congenital anomalies | 0 | 0(0-17.67) | 1 | 2.59(0.07-14.45) | 0 | 0(0-20.41) | 0 | 0(0-37.85) | 1 | 1.15(0.03-6.39) |  |  |  |  |
| Certain conditions originating in perinatal period | 0 | 0(0-5475.03) | 0 | 0(0-2646.43) | 0 | 0(0-5239.56) | 0 | 0(0-9935.49) | 0 | 0(0-1173.68) |  |  |  |  |
| Symptoms, Signs and Ill-Defined Conditions | 16 | 3.38#(1.93-5.49) | 13 | 1.68(0.89-2.87) | 3 | 0.82(0.17-2.4) | 5 | 2.42(0.79-5.64) | 37 | 2.03#(1.43-2.8) |  |  |  |  |
| Accidents and Adverse Effects | 12 | 1.54(0.8-2.69) | 22 | 1.59(1-2.41) | 10 | 1.47(0.7-2.7) | 5 | 1.23(0.4-2.88) | 49 | 1.51#(1.12-1.99) |  |  |  |  |
| Suicide and Self-Inflicted Injury | 8 | 7.23#(3.12-14.25) | 9 | 3.89#(1.78-7.38) | 1 | 0.81(0.02-4.54) | 0 | 0(0-5.55) | 18 | 3.39#(2.01-5.35) |  |  |  |  |
| Homicide and legal intervention | 0 | 0(0-15.85) | 0 | 0(0-7.55) | 0 | 0(0-16.93) | 0 | 0(0-38.19) | 0 | 0(0-3.56) |  |  |  |  |
| Other Cause of Death | 152 | 2.88#(2.44-3.38) | 129 | 1.46#(1.22-1.73) | 47 | 1.08(0.79-1.44) | 24 | 0.88(0.57-1.31) | 352 | 1.66#(1.49-1.84) |  |  |  |  |

# P value less than .05

Supplementary Table 20. Standardized-mortality ratios (SMRs) for each cause of death following CRC adenocarcinoma diagnosis in patients.

| Timing of Deaths After Diagnosis | | | | | | | | | | |  |  |  |  |
| --- | --- | --- | --- | --- | --- | --- | --- | --- | --- | --- | --- | --- | --- | --- |
| Cause of death | <1years | | 1-5years | | 5-10years | | >10years | | Total | |  |  |  |  |
| Selected Events | No. Observed | SMR(95%CI) | No. Observed | SMR(95%CI) | No. Observed | SMR(95%CI) | No. Observed | SMR(95%CI) | No. Observed | SMR(95%CI) |  |  |  |  |
| All cause of death | 50011 | 5.26#(5.21-5.31) | 94022 | 2.88#(2.86-2.89) | 37215 | 1.57#(1.55-1.58) | 13031 | 1.27#(1.25-1.30) | 50011 | 5.26#(5.21-5.31) |  |  |  |  |
| All malignant cancers | 38809 | 18.42#(18.23-18.60) | 69057 | 9.75#(9.67-9.82) | 16736 | 3.45#(3.40-3.50) | 3558 | 1.80#(1.75-1.87) | 38809 | 18.42#(18.23-18.60) |  |  |  |  |
| Colon and Rectum | 35774 | 174.08#(172.28-175.89) | 61330 | 90.17#(89.46-90.89) | 11679 | 25.84#(25.38-26.32) | 1513 | 8.49#(8.07-8.93) | 35774 | 174.08#(172.28-175.89) |  |  |  |  |
| In situ, benign, or unknown behavior neoplasms | 96 | 1.63#(1.32-1.99) | 272 | 1.32#(1.17-1.48) | 178 | 1.15(0.99-1.33) | 73 | 1.07(0.84-1.35) | 96 | 1.63#(1.32-1.99) |  |  |  |  |
| Tuberculosis | 5 | 1.92(0.62-4.48) | 8 | 0.96(0.42-1.90) | 5 | 0.95(0.31-2.22) | 5 | 2.50(0.81-5.84) | 5 | 1.92(0.62-4.48) |  |  |  |  |
| syphilis | 0 | 0(0-25.02) | 0 | 0(0-7.65) | 1 | 3.00(0.08-16.73) | 0 | 0(0-26.30) | 0 | 0(0-25.02) |  |  |  |  |
| Septicemia | 470 | 3.38#(3.08-3.70) | 660 | 1.38#(1.28-1.49) | 403 | 1.17#(1.06-1.29) | 156 | 1.05(0.89-1.23) | 470 | 3.38#(3.08-3.70) |  |  |  |  |
| Other infectious diseases | 208 | 2.75#(2.39-3.15) | 275 | 1.06(0.94-1.20) | 177 | 0.97(0.83-1.12) | 64 | 0.88(0.68-1.13) | 208 | 2.75#(2.39-3.15) |  |  |  |  |
| Diabetes Mellitus | 423 | 1.48#(1.35-1.63) | 1076 | 1.12#(1.06-1.19) | 853 | 1.29#(1.20-1.38) | 383 | 1.40#(1.26-1.55) | 423 | 1.48#(1.35-1.63) |  |  |  |  |
| Alzheimers | 191 | 0.53#(0.46-0.62) | 901 | 0.67#(0.63-0.72) | 1242 | 1.09#(1.03-1.15) | 746 | 1.32#(1.23-1.42) | 191 | 0.53#(0.46-0.62) |  |  |  |  |
| Diseasis of hearts | 4307 | 1.60#(1.55-1.65) | 9270 | 1.01(0.99-1.04) | 7210 | 1.12#(1.09-1.15) | 3227 | 1.18#(1.14-1.22) | 4307 | 1.60#(1.55-1.65) |  |  |  |  |
| Hypertension without heart disease | 170 | 1.57#(1.34-1.82) | 401 | 1.03(0.93-1.13) | 383 | 1.24#(1.12-1.37) | 178 | 1.26#(1.08-1.46) | 170 | 1.57#(1.34-1.82) |  |  |  |  |
| Cerebrovascular diseases | 869 | 1.39#(1.30-1.49) | 1935 | 0.92#(0.88-0.97) | 1475 | 1.00(0.95-1.06) | 640 | 1.02(0.94-1.10) | 869 | 1.39#(1.30-1.49) |  |  |  |  |
| Atherosclerosis | 73 | 1.65#(1.29-2.07) | 150 | 1.06(0.90-1.24) | 84 | 0.95(0.76-1.17) | 48 | 1.42#(1.05-1.89) | 73 | 1.65#(1.29-2.07) |  |  |  |  |
| Aortic aneurysm and dissection | 65 | 1.26(0.98-1.61) | 145 | 0.87(0.74-1.03) | 98 | 0.94(0.76-1.14) | 41 | 1.04(0.75-1.42) | 65 | 1.26(0.98-1.61) |  |  |  |  |
| Other diseases of arteries, arterioles, capillaries | 68 | 1.70#(1.32-2.15) | 127 | 0.94(0.78-1.11) | 111 | 1.15(0.95-1.39) | 49 | 1.20(0.89-1.58) | 68 | 1.70#(1.32-2.15) |  |  |  |  |
| Pneumonia and influenza | 367 | 1.43#(1.29-1.58) | 787 | 0.90#(0.84-0.97) | 698 | 1.13#(1.05-1.22) | 286 | 1.10(0.98-1.24) | 367 | 1.43#(1.29-1.58) |  |  |  |  |
| Chronic obstructive pulmonary disease and allied cond | 760 | 1.35#(1.26-1.45) | 1970 | 1.00(0.96-1.05) | 1571 | 1.09#(1.04-1.14) | 643 | 1.03(0.95-1.11) | 760 | 1.35#(1.26-1.45) |  |  |  |  |
| Stomach and duodenal ulcers | 32 | 2.27(1.56-3.21) | 45 | 0.99(0.72-1.32) | 34 | 1.14(0.79-1.60) | 11 | 0.92(0.46-1.64) | 32 | 2.27#(1.56-3.21) |  |  |  |  |
| Chronic liver disease and cirrhosis | 205 | 2.57#(2.23-2.95) | 319 | 1.23#(1.10-1.38) | 222 | 1.35#(1.18-1.54) | 83 | 1.31#(1.05-1.63) | 205 | 2.57#(2.23-2.95) |  |  |  |  |
| Nephritis, Nephrotic Syndrome and Nephrosis | 323 | 1.68#(1.50-1.87) | 738 | 1.09#(1.02-1.17) | 629 | 1.25#(1.15-1.35) | 246 | 1.13(1.00-1.29) | 323 | 1.68#(1.50-1.87) |  |  |  |  |
| Complications of pregnancy, childbirth, puerperium | 4 | 22.4#(6.10-57.35) | 13 | 26.55#(14.14-45.40) | 2 | 9.62(1.17-34.77) | 2 | 36.12#(4.37-130.47) | 4 | 22.40#(6.10-57.35) |  |  |  |  |
| Congenital anomalies | 13 | 1.68(0.90-2.88) | 18 | 0.72(0.43-1.14) | 13 | 0.82(0.44-97.53) | 3 | 0.48(0.10-1.41) | 13 | 1.68(0.90-2.88) |  |  |  |  |
| Certain conditions originating in perinatal period | 0 | 0(0-125.93) | 1 | 10.75(0.27-59.89) | 1 | 17.50(0.44-1.41) | 0 | 0(0-174.24) | 0 | 0(0-125.93) |  |  |  |  |
| Symptoms, Signs and Ill-Defined Conditions | 177 | 1.57#(1.35-1.82) | 421 | 1.03(0.93-1.13) | 340 | 1.03(0.93-1.15) | 135 | 0.97(0.81-1.14) | 177 | 1.57#(1.35-1.82) |  |  |  |  |
| Accidents and Adverse Effects | 265 | 1.08(0.95-1.21) | 774 | 0.91#(0.85-0.98) | 577 | 0.93(0.86-1.01) | 295 | 1.08(0.96-1.21) | 265 | 1.08(0.95-1.21) |  |  |  |  |
| Suicide and Self-Inflicted Injury | 99 | 1.88#(1.53-2.29) | 235 | 1.38#(1.21-1.57) | 122 | 1.14(0.95-1.36) | 39 | 0.95(0.67-1.30) | 99 | 1.88#(1.53-2.29) |  |  |  |  |
| Homicide and legal intervention | 9 | 0.84(0.38-1.60) | 32 | 1.02(0.70-1.45) | 21 | 1.27(0.79-1.94) | 8 | 1.47(0.63-2.89) | 9 | 0.84(0.38-1.60) |  |  |  |  |
| Other Cause of Death | 2003 | 1.45#(1.39-1.52) | 4392 | 0.88#(0.85-0.90) | 4029 | 1.00(0.97-1.03) | 2112 | 1.33#(1.08-1.18) | 2003 | 1.45#(1.39-1.52) |  |  |  |  |

# P value less than .05
